# Patient-Reported Outcome and Experience Measures in Perinatal Care to Guide Clinical Practice: Prospective Observational Study

**DOI:** 10.2196/37725

**Published:** 2022-07-05

**Authors:** Anne Louise Depla, Marije Lamain-de Ruiter, Lyzette T Laureij, Hiske E Ernst-Smelt, Jan A Hazelzet, Arie Franx, Mireille N Bekker, BUZZ Project Team

**Affiliations:** 1 Department of Obstetrics and Gynecology Wilhemina Children’s Hospital University Medical Center Utrecht Utrecht Netherlands; 2 Department of Obstetrics and Gynecology Division of Obstetrics and Fetal Medicine Erasmus Medical Center Sophia Rotterdam Netherlands; 3 Department of Public Health Erasmus Medical Center University Medical Center Rotterdam Rotterdam Netherlands; 4 See Acknowledgments

**Keywords:** perinatal care, patient-reported outcomes, patient-reported experiences, patient-centered outcome measures, value-based health care, shared decision-making, personalized care, integrated care

## Abstract

**Background:**

The International Consortium for Health Outcomes Measurement has published a set of patient-centered outcome measures for pregnancy and childbirth (PCB set), including patient-reported outcome measures (PROMs) and patient-reported experience measures (PREMs). To establish value-based pregnancy and childbirth care, the PCB set was implemented in the Netherlands, using the outcomes on the patient level for shared decision-making and on an aggregated level for quality improvement.

**Objective:**

This study aims to report first outcomes, experiences, and practice insights of implementing the PCB set in clinical practice.

**Methods:**

In total, 7 obstetric care networks across the Netherlands, each consisting of 1 or 2 hospitals and multiple community midwifery practices (ranging in number from 2 to 18), implemented the PROM and PREM domains of the PCB set as part of clinical routine. This observational study included all women participating in the clinical project. PROMs and PREMs were assessed with questionnaires at 5 time points: 2 during pregnancy and 3 post partum. Clinical threshold values (alerts) supported care professionals interpreting the answers, indicating possibly alarming outcomes per domain. Data collection took place from February 2020 to September 2021. Data analysis included missing (pattern) analysis, sum scores, alert rates, and sensitivity analysis.

**Results:**

In total, 1923 questionnaires were collected across the 5 time points: 816 (42.43%) at T1 (first trimester), 793 (41.23%) at T2 (early third trimester), 125 (6.5%) at T3 (maternity week), 170 (8.84%) at T4 (6 weeks post partum), and 19 (1%) at T5 (6 months post partum). Of these, 84% (1615/1923) were filled out completely. Missing items per domain ranged from 0% to 13%, with the highest missing rates for depression, pain with intercourse, and experience with pain relief at birth. No notable missing patterns were found. For the PROM domains, relatively high alert rates were found both in pregnancy and post partum for incontinence (469/1798, 26.08%), pain with intercourse (229/1005, 22.79%), breastfeeding self-efficacy (175/765, 22.88%), and mother-child bonding (122/288, 42.36%). Regarding the PREM domains, the highest alert rates were found for birth experience (37/170, 21.76%), shared decision-making (101/982, 10.29%), and discussing pain relief ante partum (310/793, 39.09%). Some domains showed very little clinical variation; for example, role of the mother and satisfaction with care.

**Conclusions:**

The PCB set is a useful tool to assess patient-reported outcomes and experiences that need to be addressed over the whole course of pregnancy and childbirth. Our results provide opportunities to improve and personalize perinatal care. Furthermore, we could propose several recommendations regarding methods and timeline of measurements based on our findings. This study supports the implementation of the PCB set in clinical practice, thereby advancing the transformation toward patient-centered, value-based health care for pregnancy and childbirth.

## Introduction

### Background

Currently, health care systems are moving toward high-value care, adapted to each individual patient [1,2]. These health care systems prioritize patients’ health goals in care decisions and quality improvement, above processes and clinical parameters. The transformation into a patient-centered, value-driven system is dependent on access to data that capture what matters most to patients [3-5]. Patient-reported outcome measures (PROMs) and patient-reported experience measures (PREMs) provide standardized assessment of patients’ health status or experience with health care directly from the patient [6]. Integrated into routine care, these measures can facilitate patient-provider communication, improve patients’ experiences, and enhance detection and management of their health status [7-9]. When aggregated, PROMs and PREMs foster inclusion of patients’ perspective in continuous quality improvement, along with clinical measures that have already been captured for quality performance [10].

Just as in other disciplines, perinatal care may benefit from systematic PROM and PREM assessment to enhance quality of care. Moreover, patient-reported outcomes of perinatal care, such as depression or incontinence, may have serious long-term consequences for the health of the mother and child and might currently be undervalued. The interest in, and use of, PROMs and PREMs has grown in perinatal care, but most PROMs and PREMs in this field are assessed anonymously for quality improvement or research purposes only [11], whereas PROMs and PREMs, if integrated in clinical care on an individual level, could provide perinatal caregivers an opportunity to detect symptoms and adapt care appropriately, as well as encourage patients to think, and speak, about their current well-being and experiences [12]. Nevertheless, clinical integration of PROMs and PREMs has many challenges such as selecting relevant topics, valid assessment instruments, measurement moments, and threshold values that require action [3,13,14].

**Figure 1:**
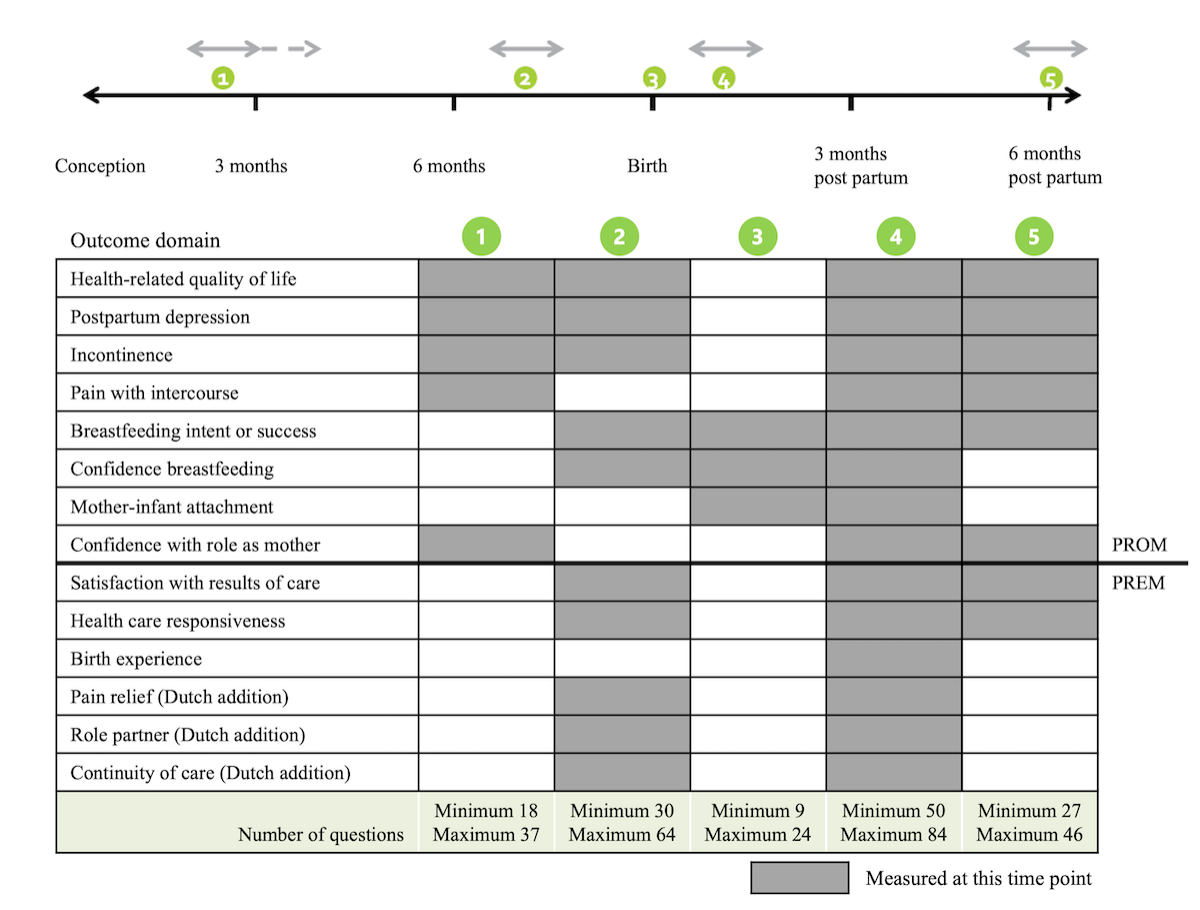
Pregnancy and childbirth outcome set: patient-reported domains and moments to measure (adapted from Nijagal et al [16]). PREM: patient-reported experience measure; PROM: patient-reported outcome measure.

The International Consortium for Health Outcomes Measurement (ICHOM) has published a core set of patient-centered outcome measures for pregnancy and childbirth (PCB set), proposing standardized measures of clinical outcomes as well as patient outcomes and experiences over the full cycle of care [15]. For its patient-reported domains, the PCB set includes measurement instruments (ie, questionnaires) and a timeline for assessment: at 5 time points throughout pregnancy and post partum until 6 months after birth (Figure 1 [16]). Recently, the feasibility and acceptability of the PCB set were studied in clinic and its patient-reported domains collected for research purposes [17-19]. In addition, some of its measurement instruments were evaluated for validity and reliability in a maternity population [20-22]. However, little is known regarding compliance with the PROM and PREM questionnaires of the PCB set and the clinical performance of threshold values that require action throughout pregnancy and the postpartum period.

### Study Rationale

In an implementation project across the Netherlands, 7 regions incorporated the PCB set in clinic over the full cycle of perinatal care with all care professionals involved. In the journey toward value-based perinatal care, the primary goal was to discuss individual PROMs and PREMs as part of regular care and use them for shared decision-making to personalize care accordingly (level 1 of value-based health care). Furthermore, aggregated PROM and PREM results could be used for patient-centered quality improvement (level 2 of value-based health care). During the project, we closely monitored first experiences and practice insights of the regions’ incorporation of patient-reported measures into routine perinatal care at an individual level. This study aimed to report compliance with the PROM and PREM questionnaires, the outcomes per domain throughout pregnancy and post partum, and the clinical use of threshold values. Our findings can support clinical implementation of value-based health care with the PCB set, accelerate the transformation toward personalized care, and contribute to governance of the PCB set to retain its international comparability.

## Methods

### Study Design

An observational study was conducted to report and gain insight into PROMs and PREMs as part of clinical routine for personalized perinatal care. This paper is written following the Strengthening the Reporting of Observational Studies in Epidemiology checklist [23].

### Setting

This study was carried out as part of a project involving the implementation of the PCB set in Dutch perinatal care called the Dutch abbreviation of Discuss Outcomes of Pregnancy with the Pregnant Woman (BUZZ) project. In total, 7 regions across the Netherlands joined forces to implement the PROM and PREM domains of the PCB set in routine clinical practice. The implementation was supported by Zorginstituut Nederland and coincided with a nationwide ministry program to enhance value-based health care and shared decision-making [24]. Each participating region consisted of 1 or 2 hospitals and 2 to 18 community midwifery practices (Table 1) collaborating in local obstetric care networks (OCNs; refer to Textbox 1 for an explanation of Dutch perinatal care organization). Data were collected from February 2020 to September 2021.

**Table 1:** Implementation strategy per obstetric care network.

	Site 1	Site 2	Site 3	Site 4	Site 5	Site 6	Site 7
Time point 1: first trimester			✓	✓	✓	✓	
Time point 2: early third trimester	✓	✓	✓	✓	✓	✓	
Time point 3: maternity week			✓	✓	✓	✓	✓
Time point 4: 6 weeks post partum		✓	✓	✓	✓	✓	✓
Time point 5: 6 months post partum			^a^		✓		✓
Collection	Stand-alone data capture tool	EHR^b^	EHR	Stand-alone data capture tool	Stand-alone data capture tool	Stand-alone data capture tool	Paper
Hospitals	1	1	1	1	1	1	2
Community midwifery practices	3	2	13	2	2	9	18
Patient group	All	All	All	Women in vulnerable situations	Diabetes or history of CS^c^	GBS+^d^	Induction with AROM^e^ by CM^f^

^a^Planned to implement at the end of the project period.

^b^EHR: electronic health record.

^c^CS: cesarean section.

^d^GBS+: urine sample positive for Group B streptococcus in pregnancy.

^e^AROM: artificial rupture of membranes.

^f^CM: community midwife.

**Organization of Dutch perinatal care.**

**Organization of Dutch perinatal care**

- Dutch perinatal care is organized in a 2-tier system.
- Community midwives provide primary care for low-risk pregnancies and act as gatekeepers to specialist care. These midwives have their own professional autonomy, responsibilities, and financial arrangements.
- For medium- to high-risk pregnancies, hospital-employed obstetric care professionals provide secondary or tertiary specialist care.
- Of all women receiving perinatal care, up to 70% visit both health care tiers [25].
- Over the last decade, a more integrated obstetric care system has been advised by the ministry of health, which is partly being realized by collaboration of both tiers in obstetric care networks.

### Participants

Women receiving perinatal care at a participating organization were invited to complete PROM and PREM questionnaires as part of usual care. Women who additionally gave informed consent to use their answers for research were included in this study. Informed consent was obtained in the PROM and PREM questionnaire itself. As this study aimed to report outcomes of the PCB set as is, we report the results of all PROM and PREM questionnaires collected within the project period; no target size was predetermined.

### Implementation in Clinical Practice

The primary purpose of the BUZZ project was to use PROM and PREM questionnaires to guide individual perinatal care. Pregnant and postpartum women were invited to fill out questionnaires as part of routine care and their obstetric care professional discussed the answers in their next regular visit. The BUZZ project was explicitly organized within OCNs to ensure continuity of care over the full cycle of care for pregnancy and childbirth. The project team of each OCN made local decisions to enhance implementation in their practice on several key points (Table 1):

- Mode of administering questionnaires: some sites could capture questionnaires through their electronic health record (EHR), others used a stand-alone data capture tool, and 1 site used paper questionnaires (whatever at that moment was considered the most optimal to use the responses in their clinical setting).
- Population and time points: most sites chose to start small by either selecting a few time points for PROM and PREM assessment or a specific patient group.
- Site-specific adaptations: some sites made minor adaptations to the questionnaire content. For example, 1 site dismissed the screening questions for depression and used the full questionnaire in all women.

### Outcome Measures

The PCB set’s PROM and PREM domains were captured as proposed by ICHOM with questionnaires at 5 time points during pregnancy and post partum (Figure 1) [16]. Each domain is assessed with its own measurement instrument, consisting of one or more questions (Multimedia Appendix 1). At every time point only relevant domains are assessed. In some domains, one or more screening questions can either rule in or rule out further questions for that domain. To fit Dutch perinatal care, a few domains have been added to the original PCB set (Figure 1) [17]. Before implementation, the translated Dutch questionnaires were tested among 4 women with low health literacy by the Dutch center of expertise on health disparities (Pharos). Minor adaptations were carried out where possible; questionnaires already validated in Dutch were not adapted. For each measurement instrument a clinical threshold value (alert) was defined according to existing literature or, if not available, determined by the multidisciplinary national BUZZ project team, informed by expert opinion (Multimedia Appendix 1). The alerts supported care professionals interpreting the answers, indicating worrisome outcomes through a color-coded dashboard (or calculated by hand in case of paper questionnaires). As clinical data could not yet be merged (digitally), a few casemix variables were collected through the questionnaires: age, gravidity, parity, postal code, and ethnicity.

### Data Analysis

Only the data of women who gave informed consent were uploaded by project leaders to a central and highly secure digital research environment. Data merging and analysis was performed on this secured server using R software (version 4.0.2; The R Foundation for Statistical Computing) [26]. Duplicate and blank questionnaires resulting from technical problems were removed. In addition, questionnaires with only the first item filled out, requesting informed consent or social support, were excluded because we could not determine whether this resulted from a technical problem. A new option to answer a question was added by 1 site (ie, *not applicable*): these answers have been considered missing in analysis because they were not included in the national (validated) scoring systems. Secondary analysis of these data was considered, but the numbers were too small. Questions that were answered unintentionally, for example, a full depression questionnaire filled out despite having scored a negative screening, were removed. The casemix variables gravidity and parity are reported as state in current pregnancy: if parity and gravidity were equal, parity was corrected to gravidity–1. Completion rates were calculated per question and per measurement instrument. If applicable, sum scores were calculated according to a predefined scoring system. Missing items were excluded from this calculation; therefore, sum scores with one or more missing items are lower by definition. Alerts were calculated according to the thresholds provided in Multimedia Appendix 1. In an additional sensitivity analysis of domains with multiple questions, results with >25% missing items were removed, and their mean sum scores and alert rates were compared with the complete analysis.

### Ethics Approval

The Medical Ethics Review Committee of the Erasmus Medical Center (MEC-2020-0129) declared that the Medical Research Involving Human Subjects Act (WMO) does not apply to this study. Therefore, it was exempt from formal medical ethics assessment. For each site, local approval was obtained from the regional ethics board.

## Results

### Overall

In total, 1923 unique questionnaires were collected, most of them during pregnancy (Table 2). The median moments of completion corresponded well with the proposed time points (Figure 1). Some T2 and T4 questionnaires were completed earlier than the proposed window, whereas a few T1 questionnaires were filled out too late. The questionnaires were filled out by 1318 individual women, of whom 838 (63.58%) completed 1 questionnaire, and the remaining 480 (36.41%) completed up to 4 questionnaires. Their baseline characteristics are presented in Table 3. Sum scores and alerts per domain and time point are presented in Tables 4 and 5. Multimedia Appendix 2 contains figures that show each domain’s scores and alerts.

**Table 2:** Moments of questionnaire completion (N=1923).

Time point	Value, n (%)	Moment of questionnaire completion, median (range)
First trimester (T1)	816 (42.43)	15 (9-27)^a^
Early third trimester (T2)	793 (41.24)	28 (23-37)^a^
Maternity week^b^ (T3)	125 (6.5)	5 (4-5)^c^
Post partum, 6 weeks^b^ (T4)	170 (8.84)	3 (0-12)^d^
Post partum, 6 months (T5)	19 (1)	27 (22-30)^d^

^a^Moment occurred in weeks of pregnancy.

^b^The exact moment of completion was missing for *maternity week* and *6 weeks post partum* for 123 and 127 questionnaires, respectively. Because of the information technology system setup, we do know that *maternity week* questionnaires were completed mostly between 1 and 3 weeks post partum and *6 weeks post partum* questionnaires between 3 and 5 weeks post partum.

^c^Moment occurred in days post partum.

^d^Moment occurred in weeks post partum.

**Table 3:** Participant characteristics (N=1318).

Characteristics			Values
Age (years), median (range); missing: n=77			32 (17-46)
**Parity, n (%); missing: n=330**			
	Nulliparous	360 (36.43)	
	Multiparous	628 (63.56)	
**Ethnicity,** **n (%); missing: n=143**			
	Western	1057 (89.96)	
	Other	118 (10.04)	

**Table 4:** Outcomes per patient-reported outcome measure domain.

Domain and subdomain		Time point	Value, n (%)	Score, median (range)	Alerts, n (%)	Missing^a^, n (%)
Social support		All	1092 (56.79)	3 (0-3)	44 (4.06)	7 (0.64)
**Quality of life**						
		All	1798 (93.5)	37 (7-50)	21 (1.17)	1 (0.06)
		T1^b^	816 (45.38)	38 (7-50)	6 (0.74)	0 (0)
		T2^c^	793 (44.1)	37 (7-50)	12 (1.52)	1 (0.13)
		T4^d^	170 (9.45)	38 (14-49)	2 (1.18)	0 (0)
		T5^e^	19 (1.06)	37 (19-46)	1 (5.26)	0 (0)
**Mental health**						
	**Screen depression**					
		All	1756 (91.32)	0 (0-6)	61 (3.52)	25 (1.42)
		T1	798 (45.44)	0 (0-6)	33 (4.19)	10 (1.25)
		T2	776 (44.19)	0 (0-5)	22 (2.85)	5 (0.64)
		T4	163 (9.28)	0 (0-5)	5 (3.27)	10 (6.13)
		T5	19 (1.08)	0 (0-4)	1 (5.26)	0 (0)
	**Full depression^f^**					
		All	103 (5.36)	10 (0-25)	47 (52.22)	13 (12.62)
		T1	51 (49.51)	11 (0-23)	27 (52.94)	0 (0)
		T2	39 (37.86)	7 (0-25)	13 (44.83)	10 (25.64)
		T4	12 (11.65)	12 (3-25)	6 (66.67)	3 (25)
		T5	1 (0.97)	N/A^g^	1 (100)	0 (0)
**Incontinence and dyspareunia**						
	**Screen, urine**					
		All	1798 (93.5)	—^h^	469 (26.91)	55 (3.06)
		T1	816 (45.38)	—	150 (20.15)	22 (2.7)
		T2	793 (44.1)	—	266 (34.64)	25 (3.15)
		T4	170 (9.45)	—	45 (27.78)	8 (4.7)
		T5	19 (1.06)	—	8 (42.1)	0 (0)
	**Screen, stool**					
		All	1798 (93.5)	—	15 (0.86)	57 (3.17)
		T1	816 (45.38)	—	3 (0.38)	23 (2.82)
		T2	793 (44.1)	—	6 (0.78)	26 (3.28)
		T4	170 (9.45)	—	6 (3.70)	8 (4.71)
		T5	19 (1.06)	—	0 (0)	0 (0)
	**Screen, flatus**					
		All	1798 (93.5)	—	388 (22.26)	55 (3.06)
		T1	816 (45.38)	—	149 (18.77)	22 (2.7)
		T2	793 (44.1)	—	190 (24.74)	25 (3.15)
		T4	170 (9.45)	—	44 (27.16)	8 (4.71)
		T5	19 (1.06)	—	5 (26.32)	0 (0)
	**Full urine^f^**					
		All	469 (24.39)	6 (0-18)	185 (39.45)	0 (0)
		T1	150 (31.98)	6 (0-15)	62 (41.33)	0 (0)
		T2	266 (56.72)	5 (1-18)	100 (37.59)	0 (0)
		T4	45 (9.59)	6 (1-15)	19 (42.22)	0 (0)
		T5	8 (1.71)	7 (3-12)	4 (50)	0 (0)
	**Full stool and flatus^f^**					
		All	394 (20.49)	3 (0-17)	385 (97.96)	1 (0.25)
		T1	151 (38.32)	3 (0-10)	147 (98)	1 (0.66)
		T2	193 (48.98)	3 (0-14)	190 (98.45)	0 (0)
		T4	45 (11.42)	3 (0-17)	43 (95.56)	0 (0)
		T5	5 (1.27)	2 (2-3)	5 (100)	0 (0)
	**Pain with intercourse**					
		All	1005 (52.26)	0 (0-5)	229 (24.65)	76 (7.56)
		T1	816 (81.19)	0 (0-5)	161 (20.72)	39 (4.78)
		T4	170 (16.91)	1 (0-5)	59 (44.36)	37 (21.76)
		T5	19 (1.89)	0 (0-5)	9 (47.37)	0 (0)
**Breastfeeding**						
	Breastfeeding intention	All (T2)	793 (41.24)	—	172 (22.4)^i^	25 (3.15)
	**Breastfeeding success**					
		All	314 (39.6)	—	116 (39.46)^j^	20 (6.37)
		T3^k^	125 (39.81)	—	45 (36)^j^	0 (0)
		T4	170 (54.14)	—	61 (40.67)^j^	20 (11.76)
		T5	19 (6.05)	—	10 (52.63)^j^	0 (0)
	**Screen, breastfeeding confidence^f^**					
		All	765 (39.78)	4 (1-5)	175 (23)	4 (0.52)
		T2	596 (77.91)	4 (1-5)	150 (25.25)	2 (0.34)
		T3	80 (10.46)	4 (2-5)	13 (16.46)	1 (1.25)
		T4	89 (11.63)	4 (1-5)	12 (13.64)	1 (1.12)
	**Full breastfeeding self-efficacy^f^**					
		All	175 (9.1)	40 (4-64)	124 (72.94)	5 (2.86)
		T2	150 (85.71)	41 (14-64)	104 (71.23)	4 (2.67)
		T3	13 (7.43)	36 (12-54)	11 (84.62)	0 (0)
		T4	12 (6.86)	27 (4-52)	9 (81.82)	1 (8.33)
**Role transition**						
	**Mother-child bonding**					
		All	288 (14.98)	2 (0-11)	122 (44.85)	16 (5.56)
		T3	125 (43.4)	2 (0-8)	56 (45.9)	3 (2.4)
		T4	163 (56.6)	2 (0-11)	66 (44)	13 (7.98)
	**Role as mother**					
		All	1005 (52.26)	4 (1-5)	3 (0.31)	40 (3.98)
		T1	816 (81.19)	4 (2-5)	1 (0.13)	26 (3.19)
		T4	170 (16.91)	5 (2-5)	1 (0.64)	14 (8.24)
		T5	19 (1.89)	5 (1-5)	1 (5.26)	0 (0)

^a^Completely missing.

^b^T1: first trimester.

^c^T2: early third trimester.

^d^T4: 6 weeks post partum.

^e^T5: 6 months post partum.

^f^Optional subdomain, dependent on screening question or questions.

^g^N/A: not applicable.

^h^Answer options were yes or no; therefore, there are no median and range values.

^i^Alert means no intention to breastfeed.

^j^Alert means feeding baby only formula.

^k^T3: maternity week.

**Table 5:** Outcomes per patient-reported experience measure domain.

Domain and subdomains		Time point		Value, n (%)	Score, median (range)	Alerts, n (%)	Missing^a^, n (%)
**Satisfaction with care**							
		All		982 (51.07)	3 (1-4)	4 (0.43)	58 (5.91)
		T2^b^		793 (80.75)	3 (1-4)	4 (0.53)	45 (5.67)
		T4^c^		170 (17.31)	4 (2-4)	0 (0)	13 (7.64)
		T5^d^		19 (1.93)	3 (2-4)	0 (0)	0 (0)
**Health care responsiveness and shared decision-making**							
		All		982 (51.07)	16 (2-16)	101 (10.67)	35 (3.56)
		T2		793 (80.75)	16 (2-16)	82 (10.72)	28 (3.53)
		T4		170 (17.31)	16 (2-16)	17 (10.43)	7 (4.12)
		T5		19 (1.93)	14 (4-16)	2 (10.53)	0 (0)
Birth experience		All (T4)		170 (8.84)	30 (8-40)	37 (23.27)	11 (6.47)
**Pain relief**							
	Information ante partum		All (T2)	793 (41.24)	1 (0-2)	310 (41.33)	43 (5.42)
	Experience at birth		All (T4)	170 (8.84)	3 (1-4)	4 (2.65)	19 (11.18)
**Partner role**							
	During pregnancy		All (T2)	793 (41.24)	3 (0-5)	56 (7.35)	31 (3.91)
	At birth		All (T4)	170 (8.84)	4 (0-5)	1 (0.66)	18 (10.59)
**Continuity of care**							
			All	963 (50.08)	11 (4-12)	55 (6.08)	58 (6.02)
			T2	793 (82.35)	11 (4-12)	49 (6.54)	44 (5.55)
			T4	170 (17.65)	11 (4-12)	6 (3.85)	14 (8.24)

^a^Completely missing.

^b^T2: early third trimester.

^c^T4: 6 weeks post partum.

^d^T5: 6 months post partum

### PROM per Domain

#### Social Support

Of the 1092 women who were asked the social support question, administered at the first time point in pregnancy that each site had implemented, 44 (4.03%) scored an alert, meaning that they had 1 or no person near them to count on in time of difficulty. A comparison of T1 and T2 showed a slightly higher alert rate at T2 (17/25, 6.8%) than at T1 (26/815, 3.19%).

#### Quality of Life

The quality-of-health domain, assessed with the Patient-Reported Outcomes Measurement Information System–Global Health Short Form, had few alerts at all time points. The alerts were based on the sum score; no alerts came from a high pain score. In additional analysis, calculation of subscores for mental and physical health showed no variation across time points.

#### Mental Health

In 3.52% (61/1731) of the women completing the 2-item depression screening (Patient Health Questionnaire-2 [PHQ-2]) an alert was scored, without variations over time. Women with an alert on the PHQ-2 filled out the full depression questionnaire (ie, Edinburgh Postnatal Depression Scale-10 [EPDS-10]). As 1 region dismissed the PHQ-2 screening questions, 29 women filled out the EPDS-10 directly. The EPDS-10 exceeded the clinical threshold in 52% (47/90) of the women, meaning that 2.67% (47/1760) of the women in the whole population screened positive for depression. The numbers with regard to the EPDS-10 results were too small to allow for interpreting variations over time.

#### Incontinence and Dyspareunia

The screening question for urine and flatus incontinence was positive in 1 of 4 women. This proportion was lower at T1 than at the other time points. Screening for stool incontinence was positive in 0.86% (15/1741) of the cases, mostly at T4 (6/162, 3.7%). The full questionnaires in case of a positive incontinence screening resulted in alert rates of 39.4% (185/469) on urine incontinence (International Consultation on Incontinence Questionnaire, Short Form) and 97.96% (385/393) on flatus or stool incontinence or both (Wexner scale). Women who screened positive for flatus incontinence but not to stool incontinence scored lower on the Wexner scale (median 3; range 0-11) than women who screened positive for stool incontinence with or without flatus incontinence (median 6; range 1-17). In 24.7% (229/929) of the women, an alert was scored on dyspareunia, with a lower alert rate at T1 than at the other time points.

#### Breastfeeding

During pregnancy, 77.6% (596/768) of the women intended to breastfeed their baby. After giving birth, 64% (80/125) of the women indicated that they would breastfeed their baby (fully or combined with formula) in the first week post partum, which decreased over time: 59% (89/150) at 6 weeks and 47% (9/19) at 6 months post partum. Of the 761 women who were breastfeeding (T3 or T4) or intended to (T2), 175 (23%) scored an alert on the screening question for confidence in breastfeeding. This alert rate was higher during pregnancy than during the postpartum period. After a positive screening question, the full breastfeeding self-efficacy questionnaire (ie, Breastfeeding Self-Efficacy Scale-10) gave an alert in 72.9% (124/170) of the cases.

#### Role Transition

The mother-child bonding questionnaire (Mother-to-Infant Bonding Scale) had a median score of 2 (range 0-11) and 44.9% (122/272) alert values. No difference was seen over time. The single question about confidence in the role as mother scored almost no alerts, and the median score was equal to the maximum score.

### PREM per Domain

#### Individual Insight Into PREMs

Before answering PREM questionnaires at T2 (early third trimester), the women could choose whether to give their care professional direct insight into their answers because the answers could affect the dependent relationship with their care professional. The answer to this question was not reported by all participating sites. We received data of 175 women, of whom 26 (14.9%) did not agree to share the answers of their PREM questionnaire directly with their caregiver.

#### Satisfaction With Care

This single-question domain, filled out by 924 women, scored almost no alerts, and the median score was 3 out of 4 (range 1-4).

#### Health Care Responsiveness and Shared Decision-making

Total scores were high, with a median of 16 (range 2-16) without variation over time. Still, the alert rate for this domain was 10.7% (101/947), based on a negative answer to one or more questions. Of the 101 women scoring an alert, 59 (58.4%) answered in the negative to just 1 of 8 questions. The alerts per question provided insight into direction for improvement, such as information provision about care decisions.

#### Birth Experience

Assessed with the 10-item Birth Satisfaction Scale, Revised, at T4, this domain gave an alert in 23.3% (37/159) of the women and had a median total score of 30 (range 8-40). The Birth Satisfaction Scale, Revised, subscales scored a median of 11 (range 2-16) for stress, 14 (range 4-16) for quality of care, and 5 (range 0-8) for women’s attributes. Comparing women with and without an alert on the sum score, the subscales stress and women’s attributes decreased by 50%, whereas the subscale quality of care decreased by 21%.

#### Pain Relief

During pregnancy, at T2, 41.3% (310/750) of the women indicated that the options for pain relief had not been discussed with their care professional yet. Post partum, most women were satisfied with the options for pain relief that were offered during childbirth.

#### Partner Role

Women were asked whether care professionals had engaged their partner enough in their care. This was insufficient for 7.4% (56/762) of the women during pregnancy and for 0.7% (1/152) during labor.

#### Continuity of Care

In total, 6.1% (55/905) of the women answered in the negative to one or more questions about continuity of care, with a median score of 11 (range 4-12). This domain had a slightly higher alert rate in pregnancy than during the postpartum period. In 96% (53/55) of the alerts, the women scored only 1 of the 3 questions negatively. Most alerts resulted from a negative answer to the question about knowing who their principal care provider was. In 23.5% (213/905) of the cases, the women had received perinatal care from just 1 care professional. Excluding these, the overall alert rate was 7.9% (55/692) and the median score 10 (range 4-12).

### Adherence to the Questionnaires

Overall, 84% (1615/1923) of the questionnaires were filled out completely. Per domain, the percentage of completely missing answers ranged between 0% and 13%, as presented in Tables 4 and 5. Certain domains were skipped more often, such as the EPDS-10 (depression) and the Patient-Reported Outcomes Measurement Information System–Sexual Function and Satisfaction (PROMIS-SFFAC102; pain with intercourse). Missing rates per question are listed in Multimedia Appendix 3 and ranged from 0% to 16%. Evaluated per question, no remarkable missing patterns were found that could not be explained by site-specific adaptations to the questions. In Multimedia Appendix 4, missing patterns per domain are visualized. In additional sensitivity analysis of domains with multiple questions, sum scores and alert rates did not significantly change after ruling out the questionnaires with >25% missing items. Here, we chose to report the complete case analysis, best reflecting clinical use, because these results were not ruled out from individual reports to care professionals.

## Discussion

### Findings and Recommendations

This study reports the results of an innovation in perinatal care in the Netherlands: implementation of ICHOM’s PROM and PREM domains for pregnancy and childbirth to guide individual patient care in 7 OCNs. The large cohort resulting from this project showed good adherence to the questionnaires. In several domains, such as incontinence and breastfeeding, the high alert rates revealed opportunities to improve and personalize perinatal care for individual women on outcomes that matter to them. In addition, our results indicate that some measurement instruments and their timing as proposed by ICHOM are less suitable for clinical use. On the basis of these findings, we present several recommendations regarding the methods and timelines of PROM and PREM assessment in clinical practice.

Overall, adherence to the questionnaires was good, similar to PROM adherence when used for routine oncologic care [7]. High missing rates per instrument could be explained by technical issues, site-specific adaptation to the questionnaires, or questions addressing a relatively taboo subject, such as those included in the EPDS-10 and PROMIS-SFFAC102 (depression and pain with intercourse, respectively). In preimplementation tests, the PROMIS-SFFAC102 question also seemed difficult to understand despite language adjustments. Adapting the answer options might help, or an alternative instrument should be selected. Although they may be imperfect, the questions on these taboo subjects were answered by most women. Especially, these taboo subjects create more awareness at both patient and care professional levels, thereby increasing the likelihood of problems being recognized and addressed in clinic.

Median moments of completion corresponded well with the timeline of data collection as proposed by ICHOM. In contrast to the provider expectations described by Chen et al [27], the questionnaire administered shortly after childbirth (T3) resulted in a large group of respondents in this study who completed them mostly within 2 weeks post partum. At this point, there is an excellent opportunity to improve breastfeeding outcomes and mother-child bonding. As final maternal checkup with an obstetric care professional is at 6 weeks post partum in the Netherlands, the questionnaire at 6 months post partum (T5) is practically difficult to arrange for care providers. As a result, most OCNs chose to skip T5 to enhance feasibility; thus, few questionnaires were collected. Although practically challenging, patient views on this timing should be considered because this moment previously has been shown to be valuable to reflect on long-term recovery after pregnancy and childbirth [17,28].

Our findings in the mental health domain indicate that the first instrument of the 2-step screening (PHQ-2) is missing an unacceptable proportion of women at risk for depression, in line with the findings of Slavin et al [21]. The prevalence of perinatal depression has been reported at a rate of 7% to 20% during pregnancy and up to 22% in the first year post partum [29]. In our cohort, the prevalence of depressive symptoms was only 2.7% over the whole period of pregnancy and childbirth up until 6 months post partum. As the main purpose in clinical care is to identify women at high risk for depression, we strongly recommend removing the PHQ-2 and screening all women for depressive complaints with the EPDS-10, despite an increased response burden. The EPDS-10 has been thoroughly validated and has been shown to be acceptable to women in pregnancy and post partum [30,31]. Furthermore, 2 PREM domains showed striking results. Women answered almost always in the positive to the PREM *satisfaction with results of care*, despite multiple PROM alerts suggesting that their results were not as positive. This might be explained by women expecting incontinence to be a *normal* result of pregnancy and childbirth. Either way, this single question did not differentiate between women who were satisfied and those who were unsatisfied with their care and does not add value to shared decision-making or quality improvement. The PREM on information provision about pain relief options gave unexpected high alerts: 41.3% (310/750) of the care professionals had not discussed this yet with their patient. This might indicate that the timing of the assessment does not fit clinical practice because the T2 questionnaire was completed at 28 weeks of pregnancy on average and regular pathways plan to discuss pain relief later. Overall, each domain in need of adjustment based on our results is listed in Textbox 2, along with proposed adaptations to enhance their use in clinical practice.

In several domains, high alert rates revealed opportunities to adapt care accordingly and improve individual outcomes. For example, a high prevalence of incontinence and pain with intercourse was found over the course of pregnancy, as expected from previous research on these topics [32]. Breastfeeding success rates were low, which corresponds to provider-reported breastfeeding numbers in the Netherlands from 2018 [33]. Strikingly, many alerts were scored on breastfeeding confidence and self-efficacy during pregnancy. This provides important opportunities for all perinatal care professionals involved to improve breastfeeding outcomes. At the same time, threshold values for alerts on several instruments must be evaluated for clinical use to determine whether women scoring an alert want help and whether clinicians have the instruments to provide this help. For example, the threshold for the Mother-to-Infant Bonding Scale was set quite low based on the literature [34,35], resulting in many alerts on mother-child bonding. At this moment, it is unknown whether women want their care professional to address these alerts, and clinical guidelines on when and how to act are lacking [36]. However, in perinatal care too, structural PROM monitoring did create openings for dialogue between patients and care professionals to personalize and improve care on these themes [2].

Regarding experience domains, 85.1% (149/175) of the women in this study agreed to making their individual answers to PREMs visible to their care professionals, but the remaining 14.9% (26/175) disagreed. These numbers both affirm the acceptability of individual PREM use and underline the importance of providing women an opportunity to choose, considering their dependent relationship with care professionals. In general, evaluating results of all women, the sum scores of the PREM instruments often did not differentiate very much, but separate answers gave valuable information about directions for improvement. For example, most alerts in the domains continuity and health care responsiveness resulted from negative answers to specific items: about knowing their principal care professional and information provision, respectively. In birth experience, the PREM with the highest alert rate, the subscales most affected in women with an alert on the sum score were stress and women’s attributes. Until now, the literature on individual PREM use to guide clinical practice has been scarce because anonymous use is mostly advocated, for quality improvement only [17,37].

**Proposed adaptations to pregnancy and childbirth set content.**

**Mental health**

- Remove Patient Health Questionnaire-2 and use only the Edinburgh Postnatal Depression Scale-10 to screen depressive symptoms because current 2-step screening rules out too many women at risk for perinatal depression.

**Incontinence**

- Use the first question of the International Consultation on Incontinence Questionnaire, Short Form, and first 3 questions of the Wexner scale as screening questions because they ask the same questions as the current screening questions. The current screening questions create an unnecessary response burden and have led to inconsequential answers.

**Pain with intercourse**

- Adjust the answer options or replace the instrument considering its relatively high missing rate and signs that the question is hard to understand.

**Role as mother**

- Replace with another instrument because this single question does not differentiate between women who were confident and those who were insecure in their role as mother. As patients proposed this subject originally, it should be maintained in the pregnancy and childbirth set [16].

**Satisfaction with care**

- Remove or replace with another instrument because this question does not differentiate between women who were satisfied and those who were unsatisfied with their care or provide insight into the direction for improvements.

**Pain relief**

- Measurement at T2 (early third trimester) is often too early because most perinatal care professionals discuss pain relief options later in the care path. We recommend involving patients to determine the optimal timing in pregnancy to discuss options for pain relief during childbirth.

**Social support**

- Ask it at each time point because women’s social networks can change throughout pregnancy and post partum. This domain was originally designed as a casemix factor but is used in clinical practice also as an outcome to act upon.

**Before asking questions about patient experiences**

- Ask the woman whether her answers to the patient-reported experience measure questions may be made visible to her care professional individually because women are in a dependent relationship with their care professionals.

### Strengths and Limitations

To our knowledge, this project was one of the first experiences with incorporating the complete PCB set into clinical practice to guide individual perinatal care. Although it was challenging, each participating site collaborated with a multidisciplinary transmural team of care professionals (part of an OCN) for implementation to ensure continuity of care over the whole cycle of care in a patient-centered approach. For this study, we have performed thorough additional analyses such as sensitivity analysis and appraisal of the use of screening questions, leading to practice implications for several domains. The sample size was large, and our results reflect the true clinical use of all patient-reported domains in the PCB set in various settings across the Netherlands. Nevertheless, because of this practical and local approach, nonresponders were not registered; therefore, we cannot report any response rates. In addition, variation over time in our results should be interpreted with caution because of different numbers of results per time point—especially, the numbers at 6 months post partum were too small to enable drawing any conclusions. Another limitation was the absence of questionnaire translations, restricting the participants to Dutch-speaking women only. Moreover, because no resources were available to support completion of the questionnaires, women with low (digital) health literacy are likely to be underrepresented, although women with language barriers or low health literacy probably have higher prevalence of pregnancy-related issues and thus greater opportunities to improve their outcomes [38]. This reveals an important concern regarding the transformation to value-based care: it could worsen existing health inequities even further. Therefore, efforts should be made to standardize the questionnaires to facilitate translation into multiple languages. Furthermore, when implementing PROMs and PREMs as part of value-based care, all stakeholders involved should be well informed about their purpose and supported with multiple solutions to embed the PCB set structurally in clinic; for example, through group consultations [39].

### Implications for Practice

On the basis of the first efforts to incorporate the PCB set into clinical practice, we have proposed several adaptations to its content and structure to better fit routine perinatal care (Textbox 2). At the same time, international governance of the PCB set is essential to maintain comparability for care improvement purposes. In addition, although we tested their clinical usefulness, further validation is needed of all the measurement instruments and their clinical thresholds during pregnancy and post partum, which has been started successfully in another cohort [20-22]. Although the numbers per region could not be compared because of differences in pilot setup (eg, patient group selection), data capture was more feasible when PROMs could be embedded in their own EHR. When used in performance management, PROM and PREM results would preferably be merged with clinical outcomes, ideally through the EHR. Although beyond our main scope, merging patient-reported data with clinical outcomes from EHRs was explored in this project. In concordance with previous findings [40], this seemed very challenging, depending on the software systems available. This study focused on the content of the PCB set; future work should investigate other factors influencing implementation in the patient, care professional, and organization contexts [41].

### Conclusions

This study shows that the PCB set is a useful tool to capture and discuss patient-reported outcomes and experiences that need attention during pregnancy, childbirth, and post partum. These are promising findings in the journey toward patient-centered, personalized, and value-based perinatal care. In the future, merging patient-reported data with clinical outcomes and casemix factors would be even more valuable to improve quality of health care both at an individual level and an aggregated level.

## Appendix

Multimedia Appendix 1 Overview of the pregnancy and childbirth outcome set: structure, scoring, and alert values.

Multimedia Appendix 2 Outcomes per domain and time point.

Multimedia Appendix 3 Missing rates per question.

Multimedia Appendix 4 Missing patterns per domain.

## Abbreviations

BUZZ: Dutch abbreviation of Discuss Outcomes of Pregnancy with the Pregnant Woman
EHR: electronic health record
EPDS-10: Edinburgh Postnatal Depression Scale-10
ICHOM: International Consortium for Health Outcomes Measurement
OCN: obstetric care network
PCB set: set of patient-centered outcome measures for pregnancy and childbirth
PHQ-2: Patient Health Questionnaire-2
PREM: patient-reported experience measure
PROM: patient-reported outcome measure
PROMIS-SFFAC102: Patient-Reported Outcomes Measurement Information System–Sexual Function and Satisfaction
