# Supplementary material for: Patient-Reported Outcome and Experience Measures in Perinatal Care to Guide Clinical Practice: Prospective Observational Study
Source: J Med Internet Res. 2022 Jul 5;24(7):e37725. doi: 10.2196/37725 (PMC9297146; doi:10.2196/37725)
Supplement: Multimedia Appendix 2 [file jmir_v24i7e37725_app2.pdf]

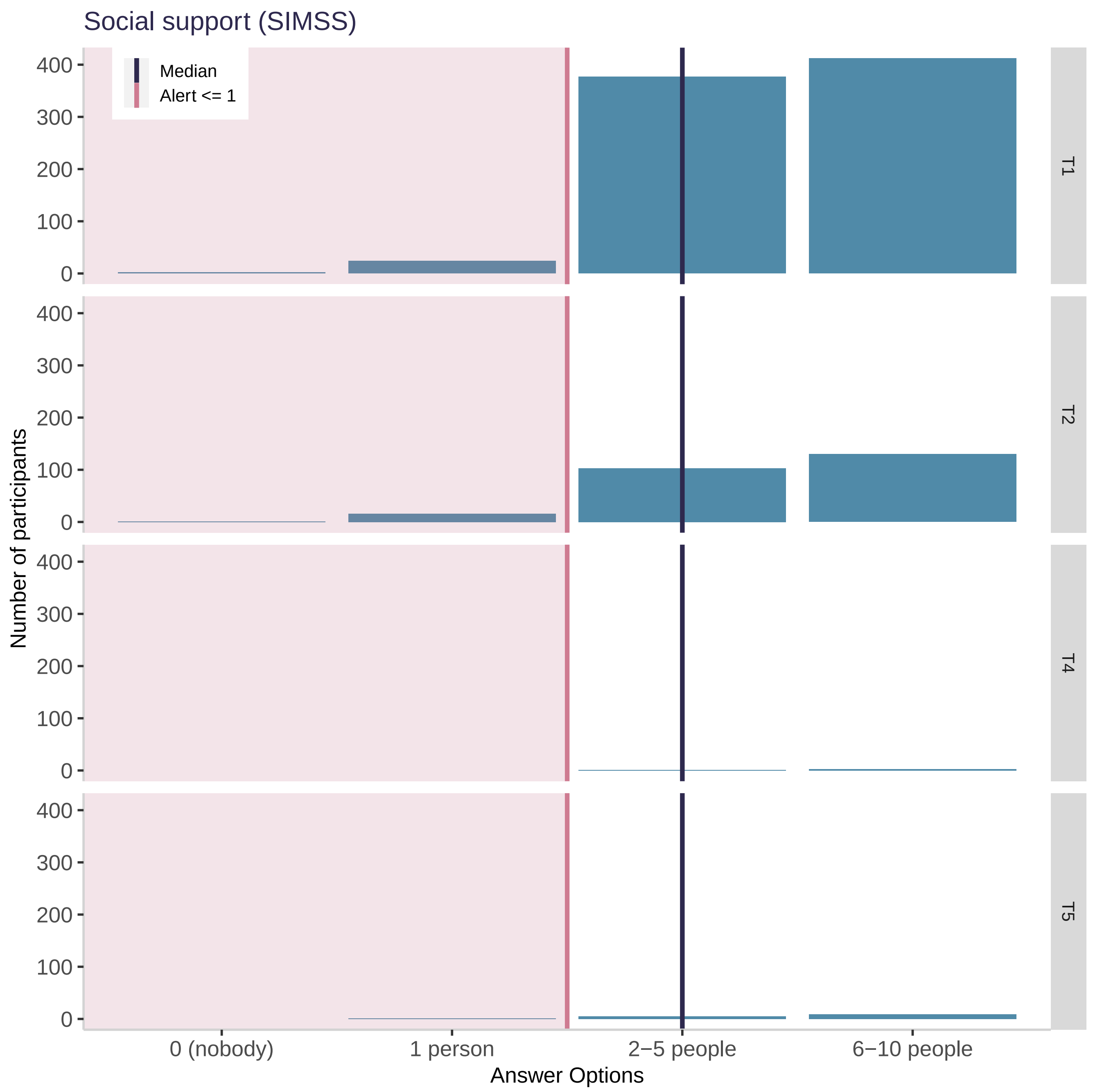

Social support (SIMSS)
Median
Alert <= 1
Number of participants
400
300
200
100
0
T1
T2
T4
T5
0 (nobody)
1 person
2−5 people
6−10 people
Answer Options

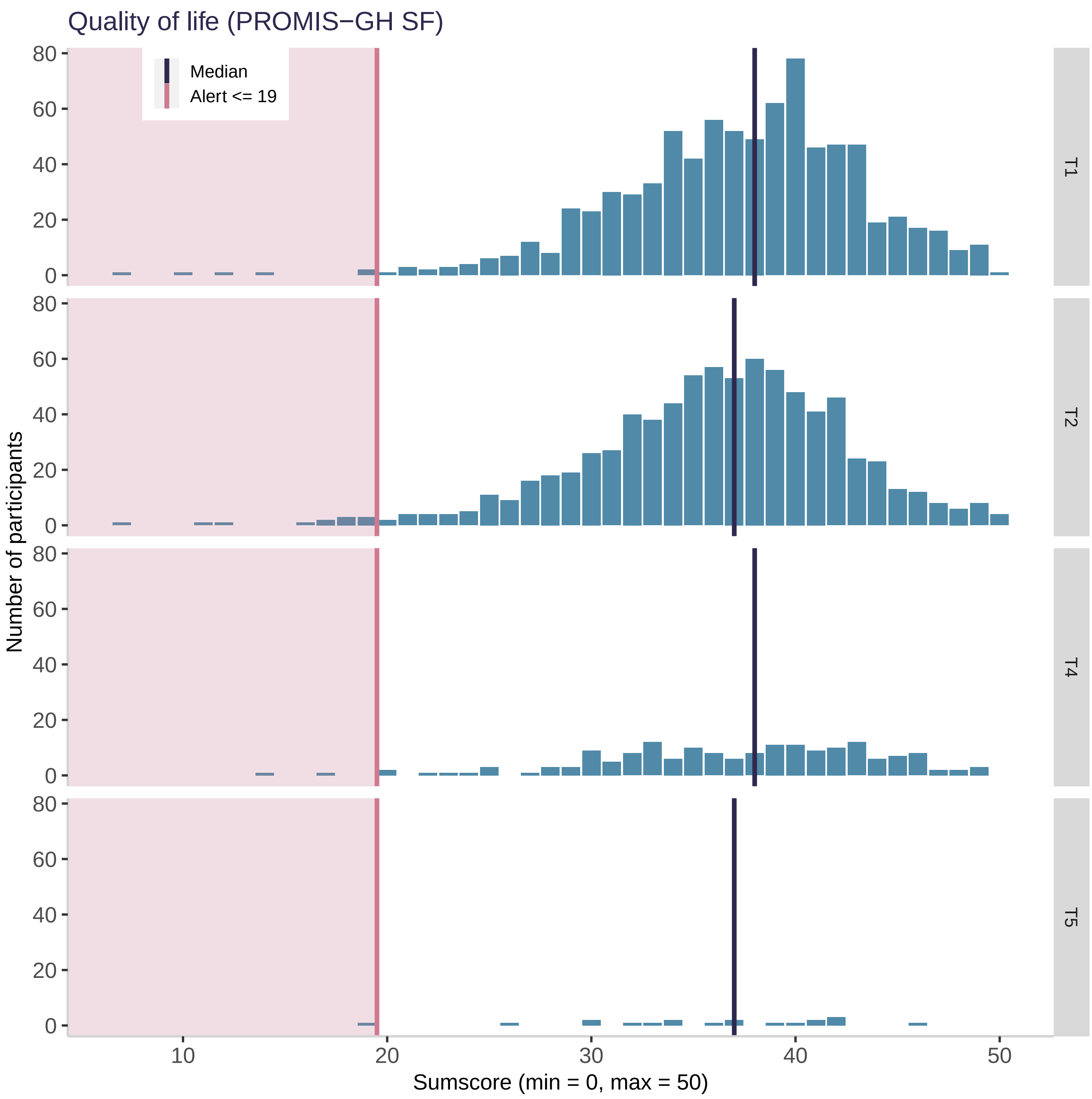

Quality of life (PROMIS−GH SF)
Median
Alert <= 19
Number of participants
Sumscore (min = 0, max = 50)
T1
T2
T4
T5
0
20
40
60
80
10
20
30
40
50

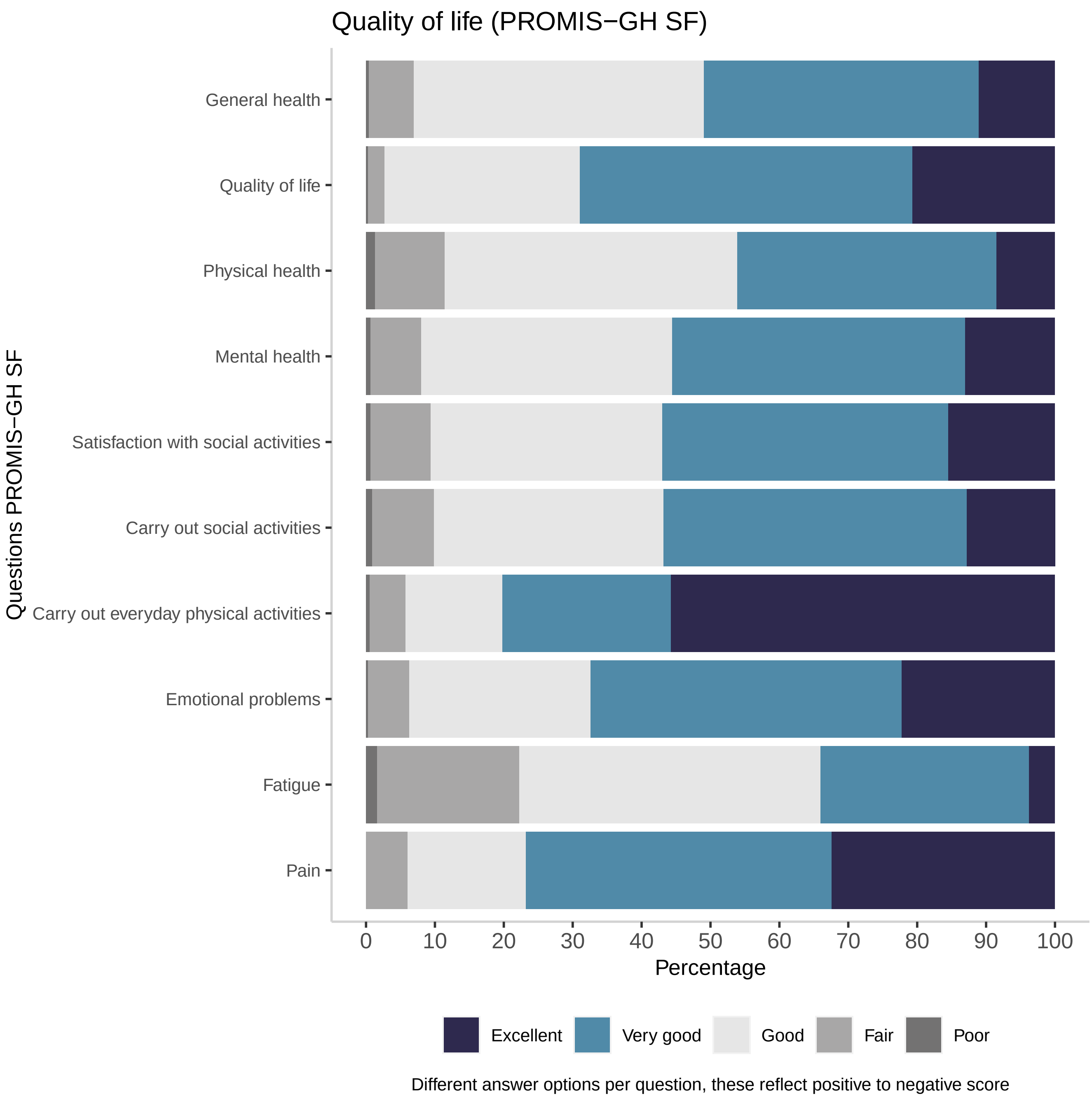
Quality of life (PROMIS−GH SF)
Questions PROMIS−GH SF
General health
Quality of life
Physical health
Mental health
Satisfaction with social activities
Carry out social activities
Carry out everyday physical activities
Emotional problems
Fatigue
Pain
0
10
20
30
40
50
60
70
80
90
100
Percentage
Excellent
Very good
Good
Fair
Poor
Different answer options per question, these reflect positive to negative score

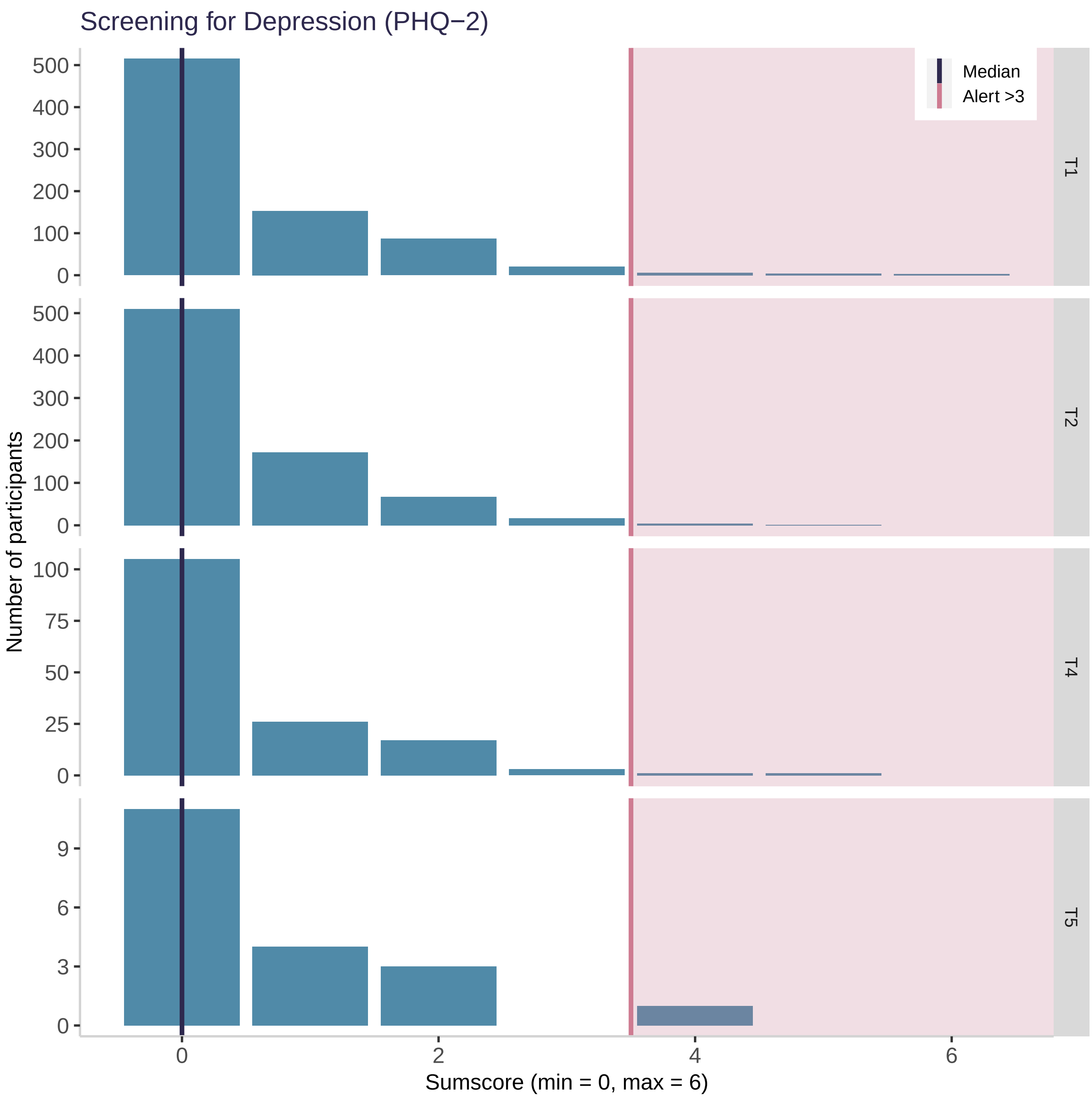

Screening for Depression (PHQ−2)
Median
Alert >3
T1
T2
T4
T5
Number of participants
Sumscore (min = 0, max = 6)
500
400
300
200
100
0
100
75
50
25
9
6
3
2
4

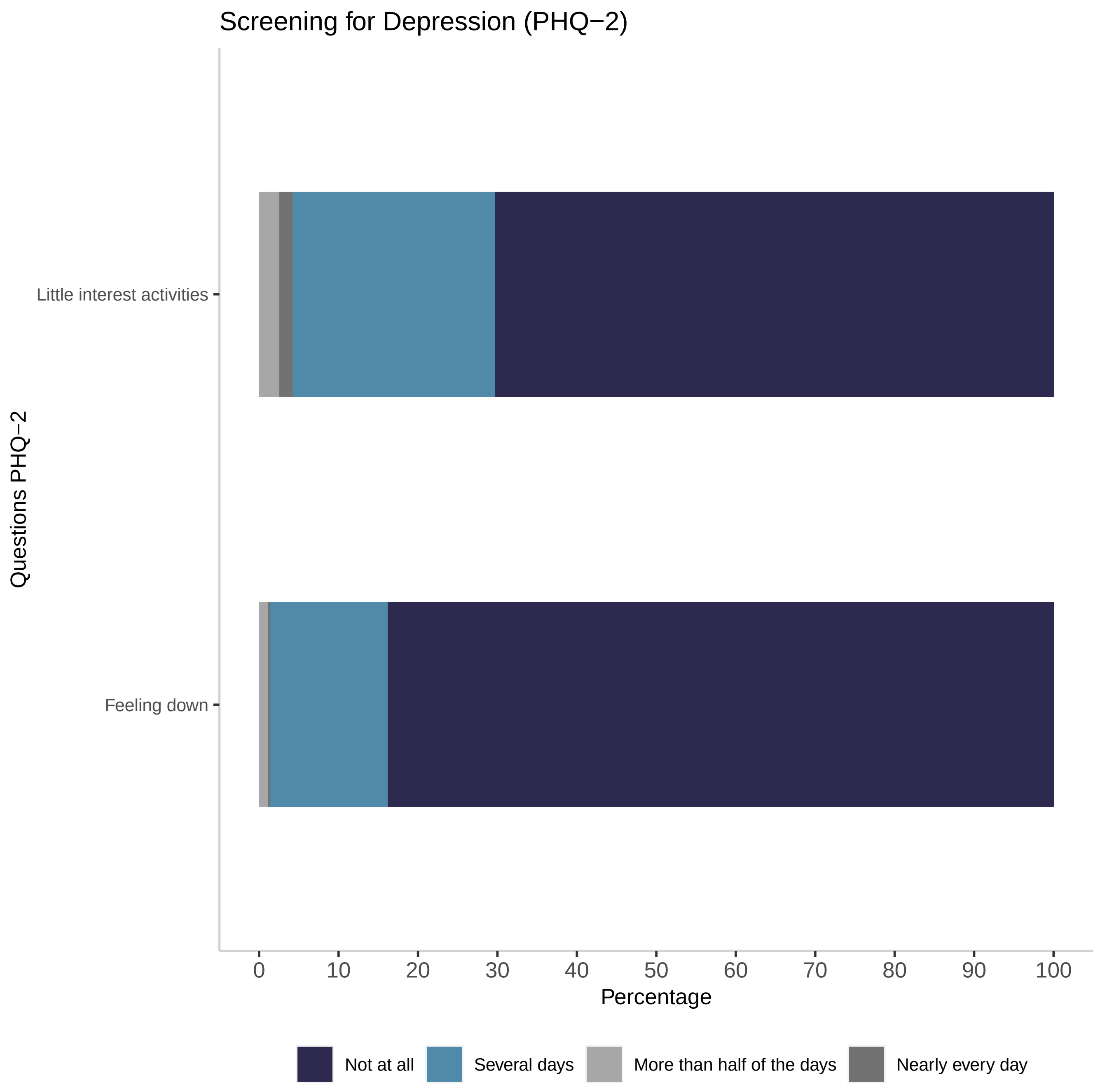

Screening for Depression (PHQ−2)
Questions PHQ−2
Little interest activities
Feeling down
0
10
20
30
40
50
60
70
80
90
100
Percentage
Not at all
Several days
More than half of the days
Nearly every day

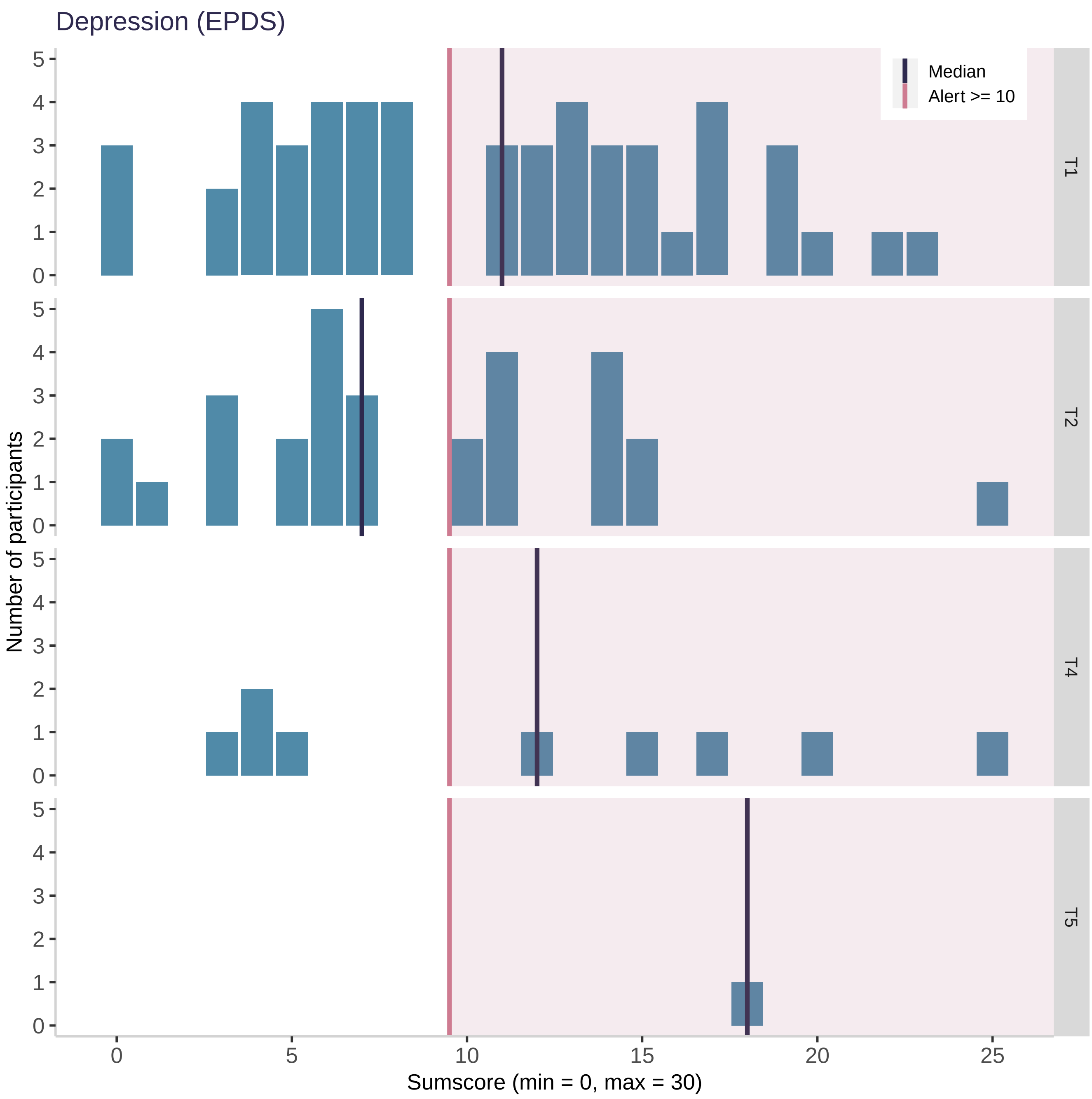
Depression (EPDS)
Median
Alert >= 10
T1
T2
T4
T5
Number of participants
Sumscore (min = 0, max = 30)
0
5
10
15
20
25
0
1
2
3
4
5

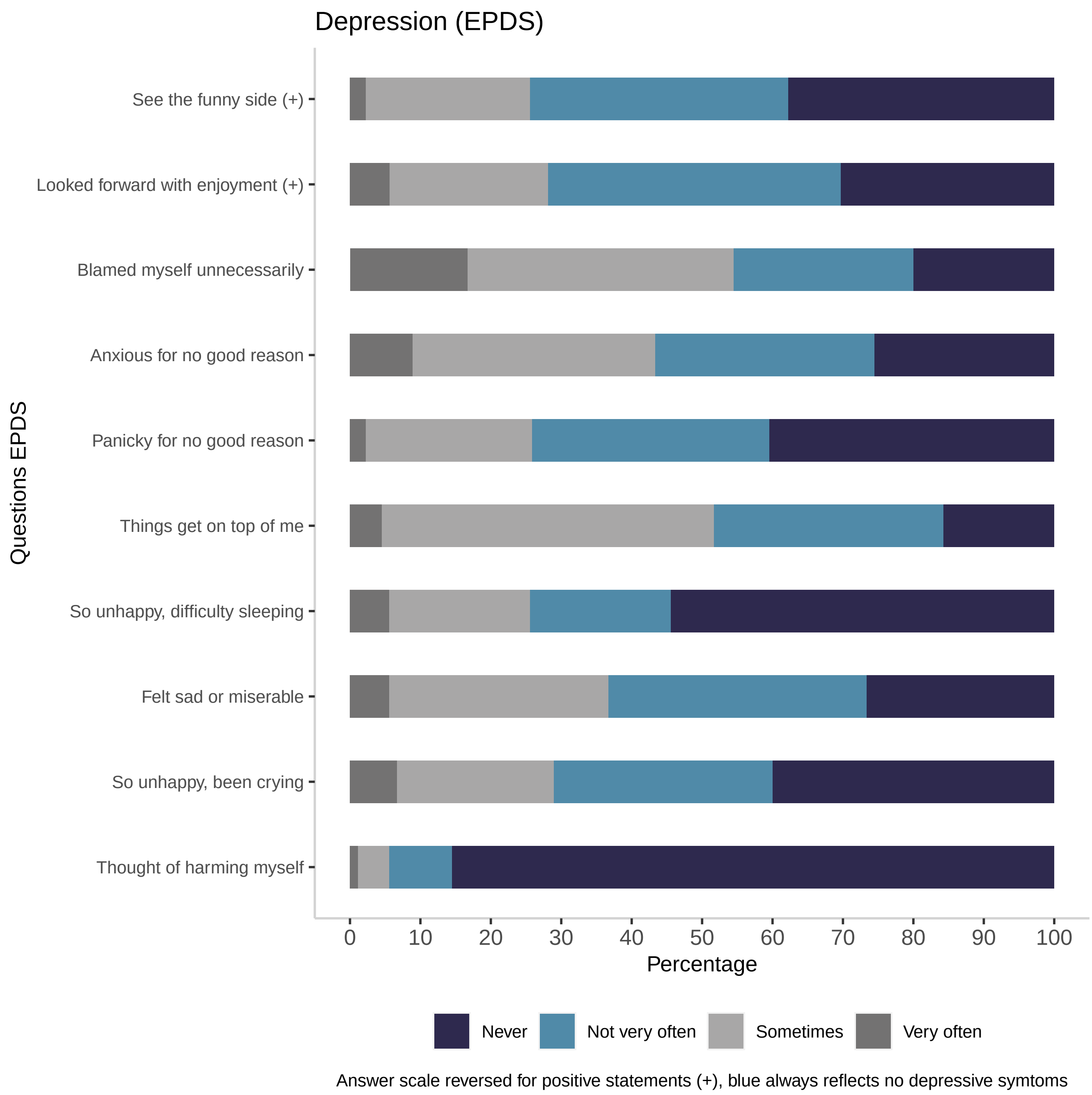

Depression (EPDS)
See the funny side (+)
Looked forward with enjoyment (+)
Blamed myself unnecessarily
Anxious for no good reason
Panicky for no good reason
Things get on top of me
So unhappy, difficulty sleeping
Felt sad or miserable
So unhappy, been crying
Thought of harming myself
Questions EPDS
0
10
20
30
40
50
60
70
80
90
100
Percentage
Never
Not very often
Sometimes
Very often
Answer scale reversed for positive statements (+), blue always reflects no depressive symtoms

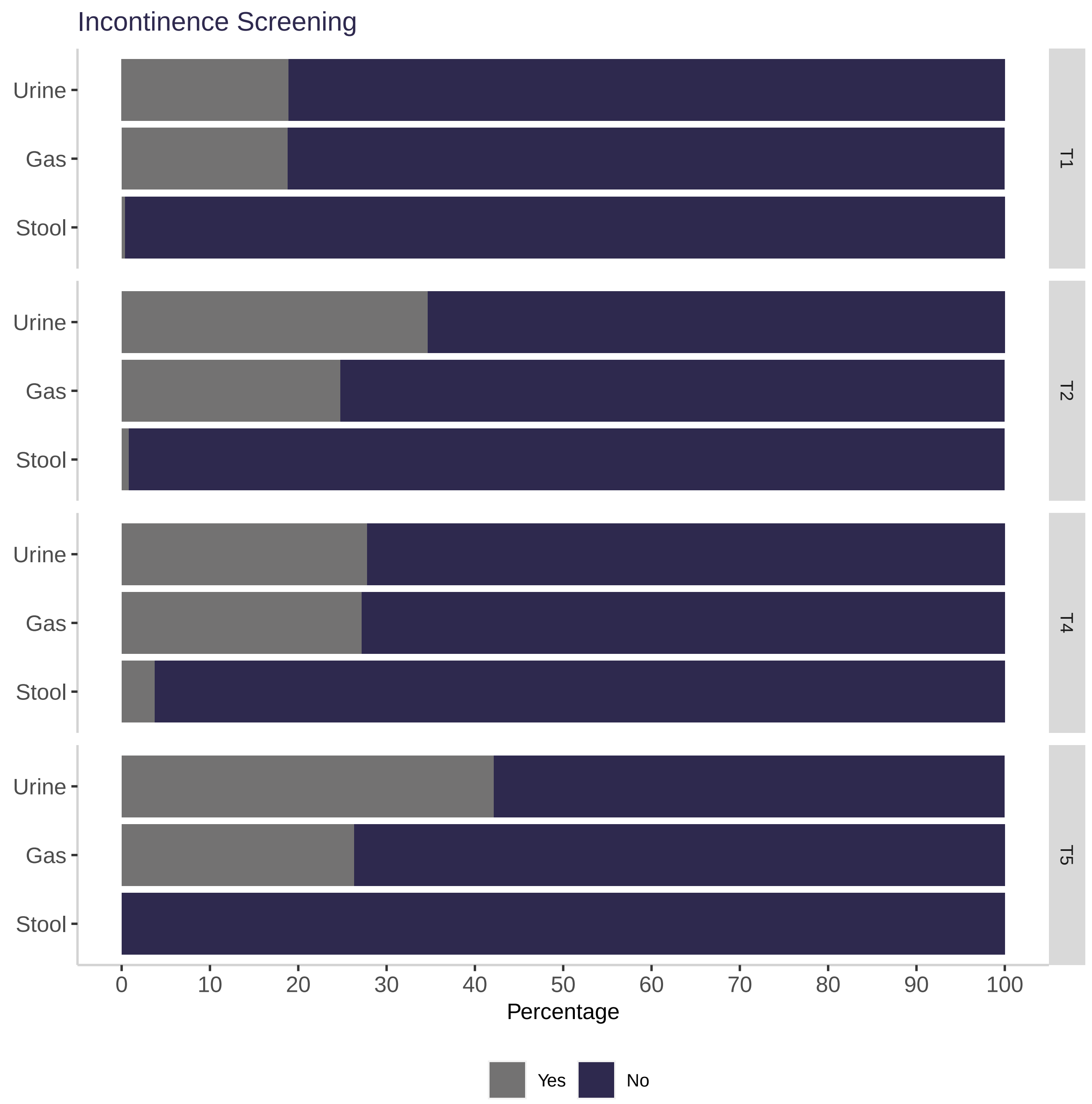

Incontinence Screening
Urine
Gas
Stool
T1
T2
T4
T5
0
10
20
30
40
50
60
70
80
90
100
Percentage
Yes
No

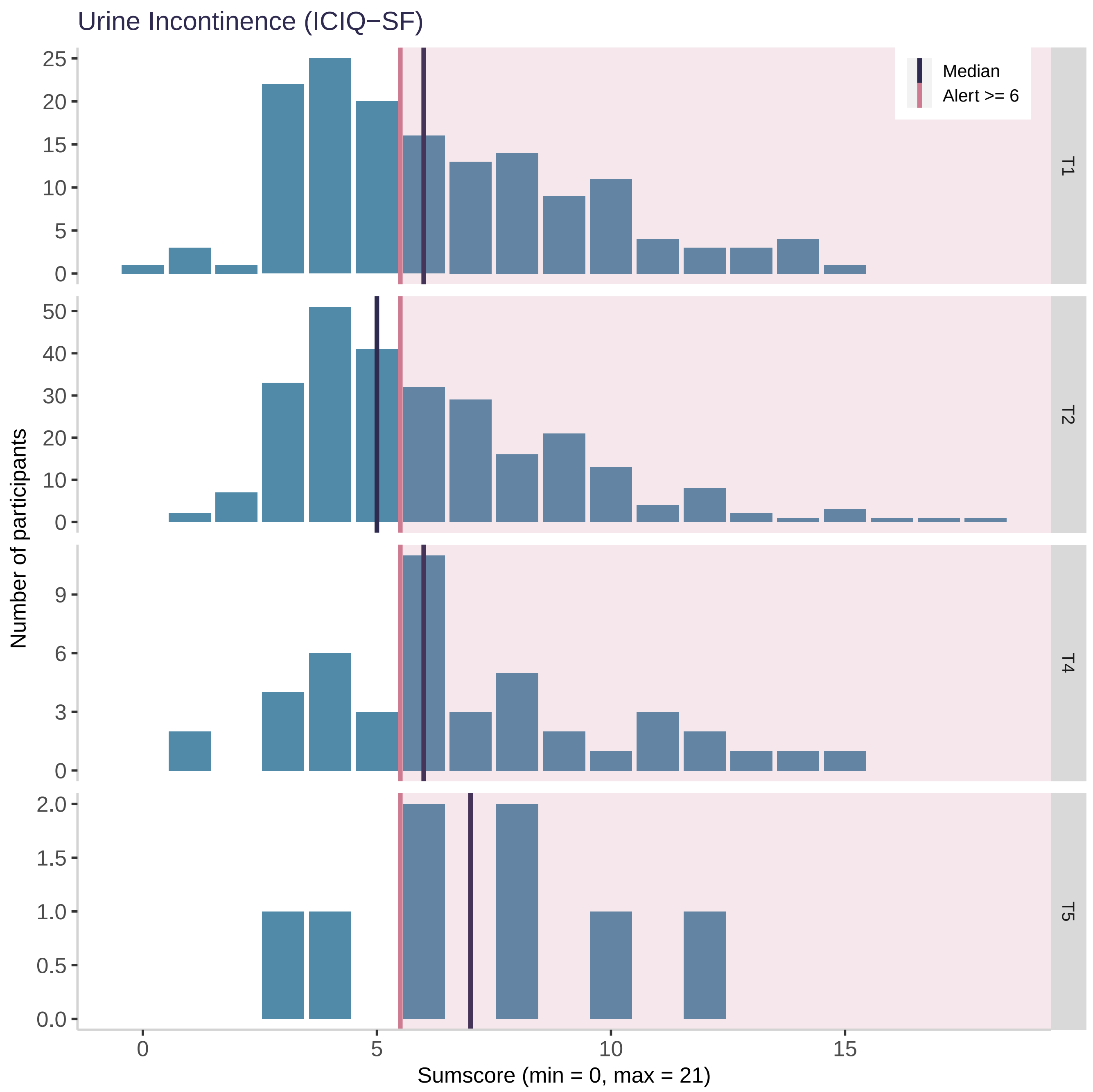

Urine Incontinence (ICIQ−SF)
Median
Alert >= 6
T1
T2
T4
T5
Number of participants
Sumscore (min = 0, max = 21)

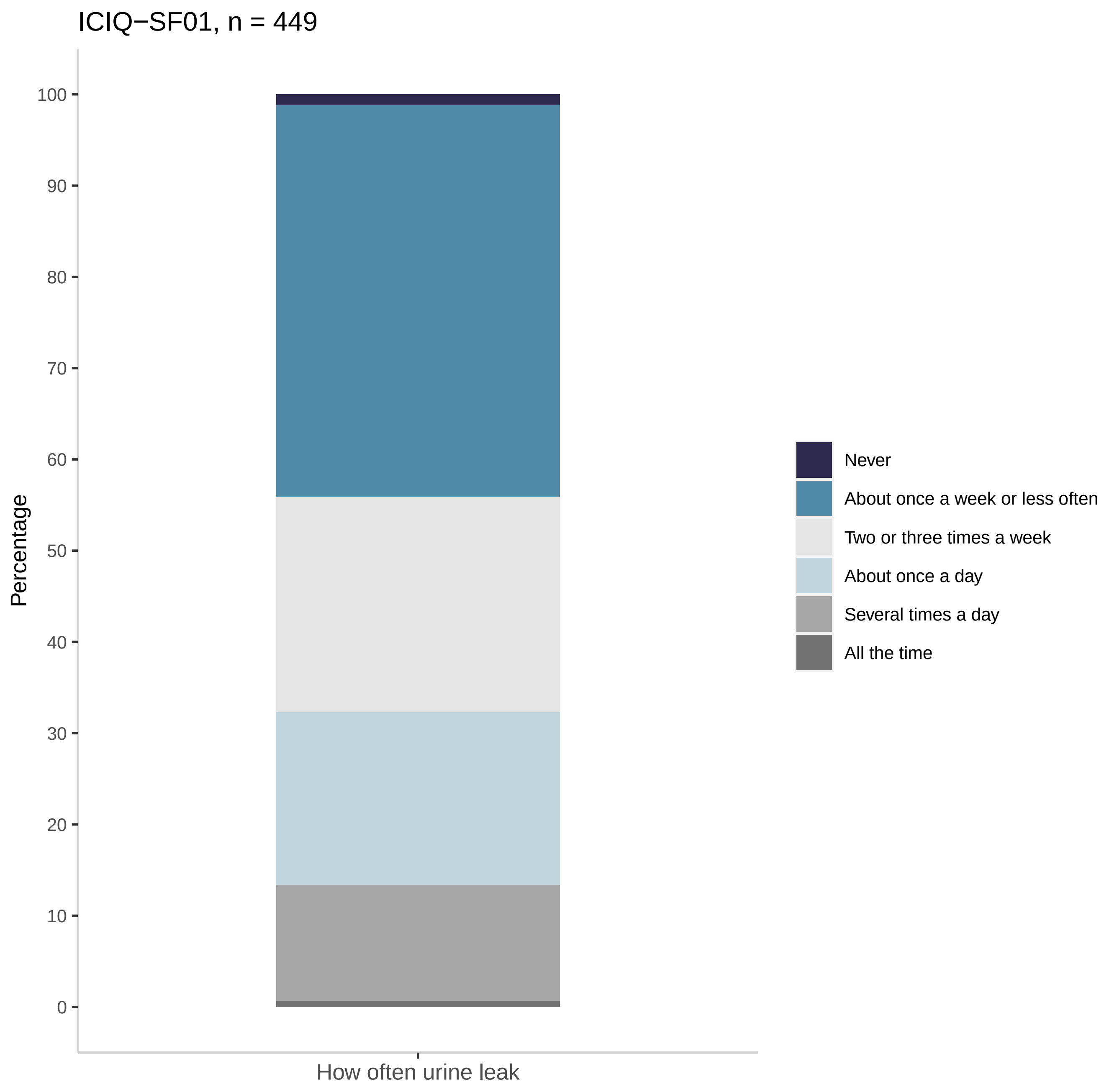
ICIQ−SF01, n = 449
Percentage
100
90
80
70
60
50
40
30
20
10
0
How often urine leak
Never
About once a week or less often
Two or three times a week
About once a day
Several times a day
All the time

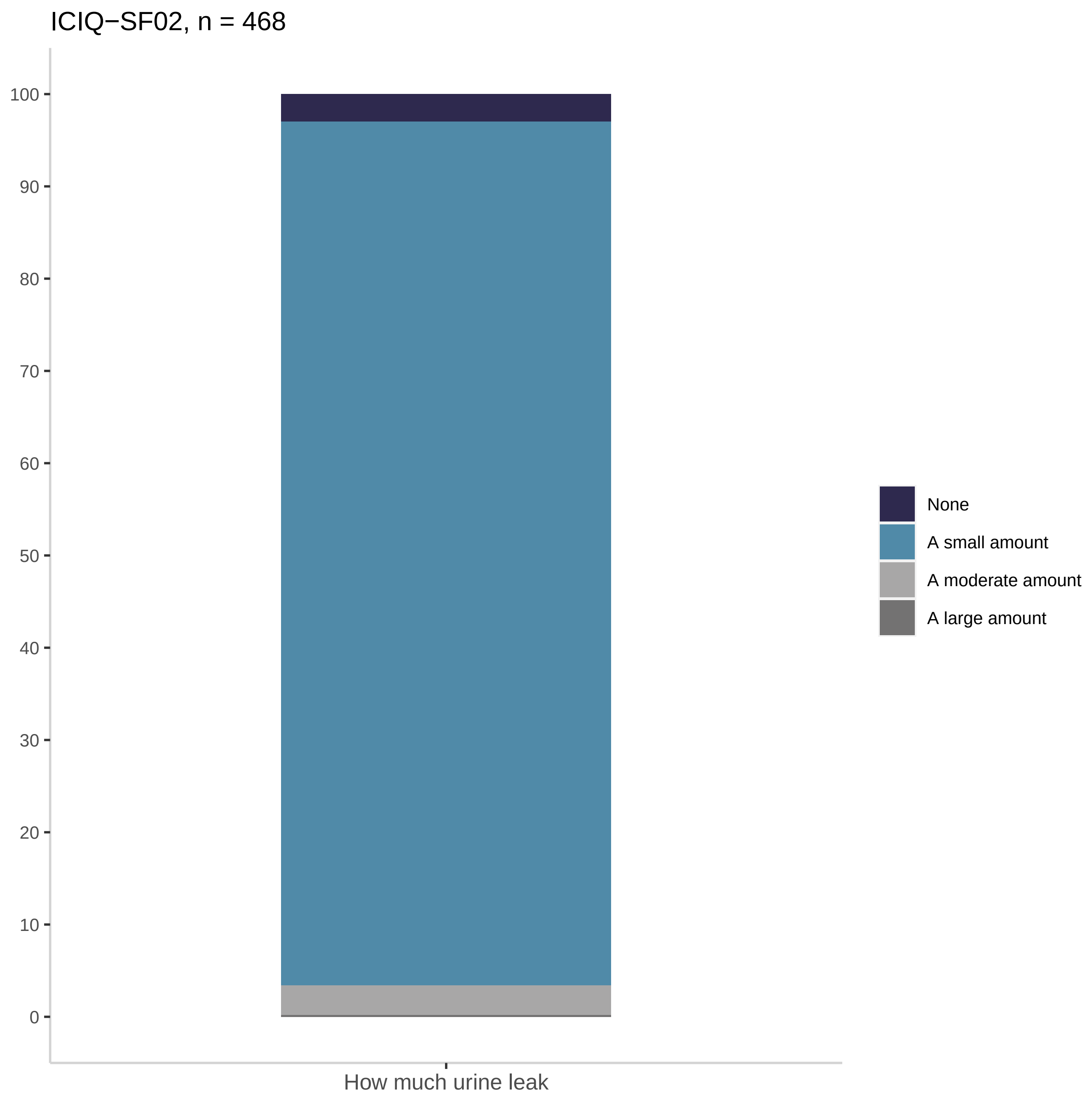
ICIQ−SF02, n = 468
100
90
80
70
60
50
40
30
20
10
0
How much urine leak
None
A small amount
A moderate amount
A large amount

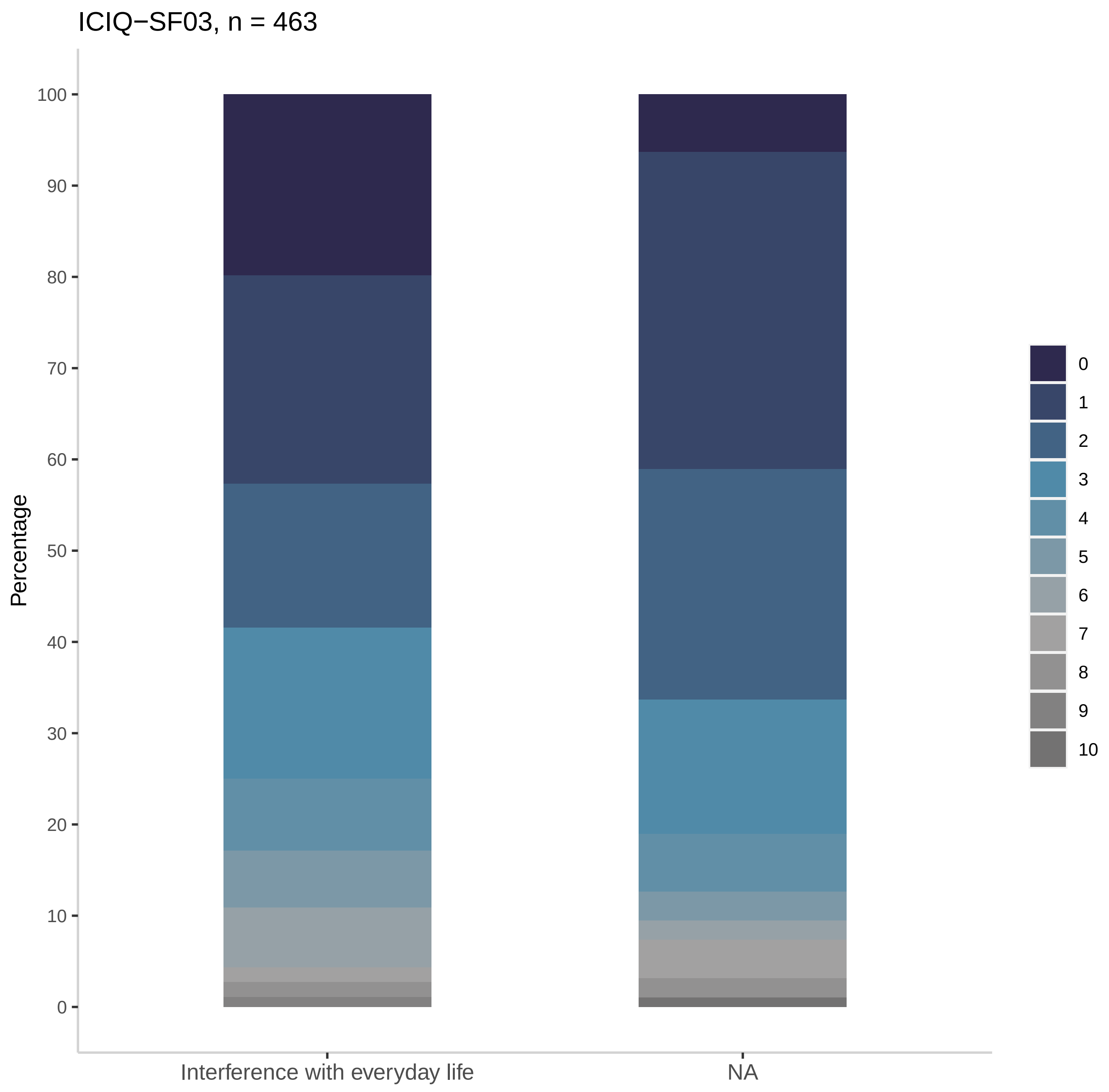

ICIQ−SF03, n = 463
Percentage
100
90
80
70
60
50
40
30
20
10
0
Interference with everyday life
NA
0
1
2
3
4
5
6
7
8
9
10

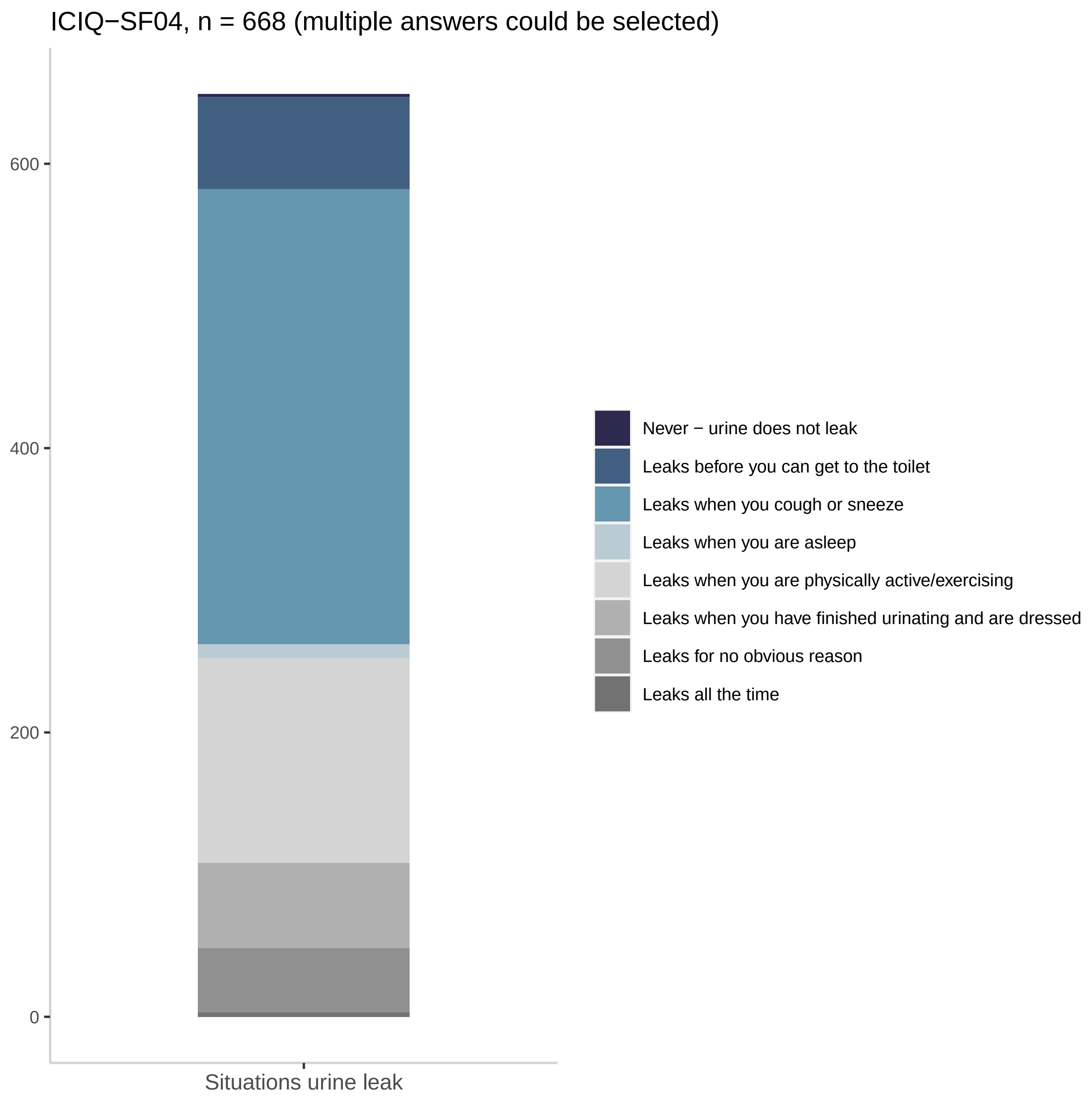

ICIQ−SF04, n = 668 (multiple answers could be selected)
600
400
200
0
Situations urine leak
Never − urine does not leak
Leaks before you can get to the toilet
Leaks when you cough or sneeze
Leaks when you are asleep
Leaks when you are physically active/exercising
Leaks when you have finished urinating and are dressed
Leaks for no obvious reason
Leaks all the time

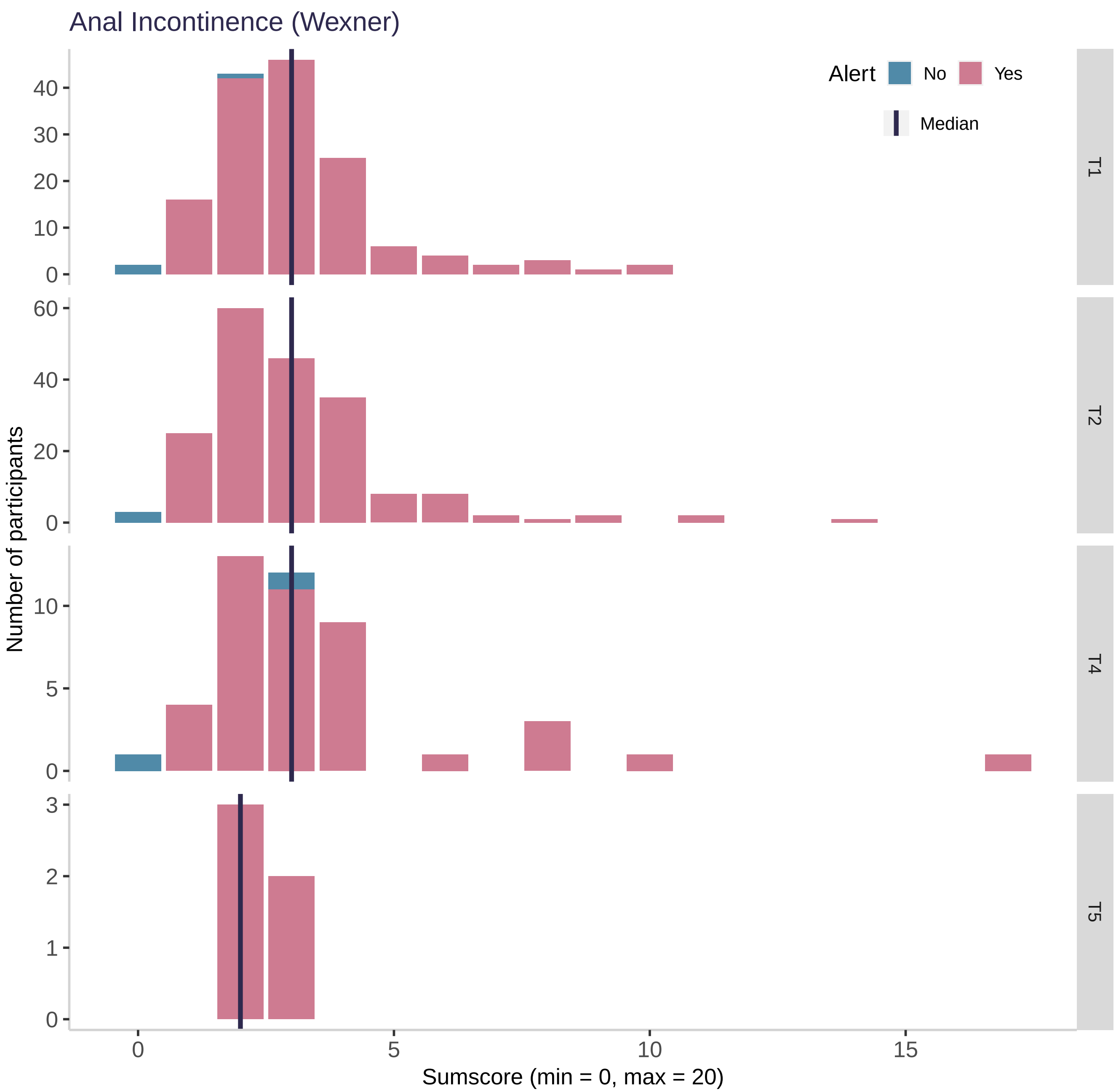

Alerts based on single questions, not on sumscore

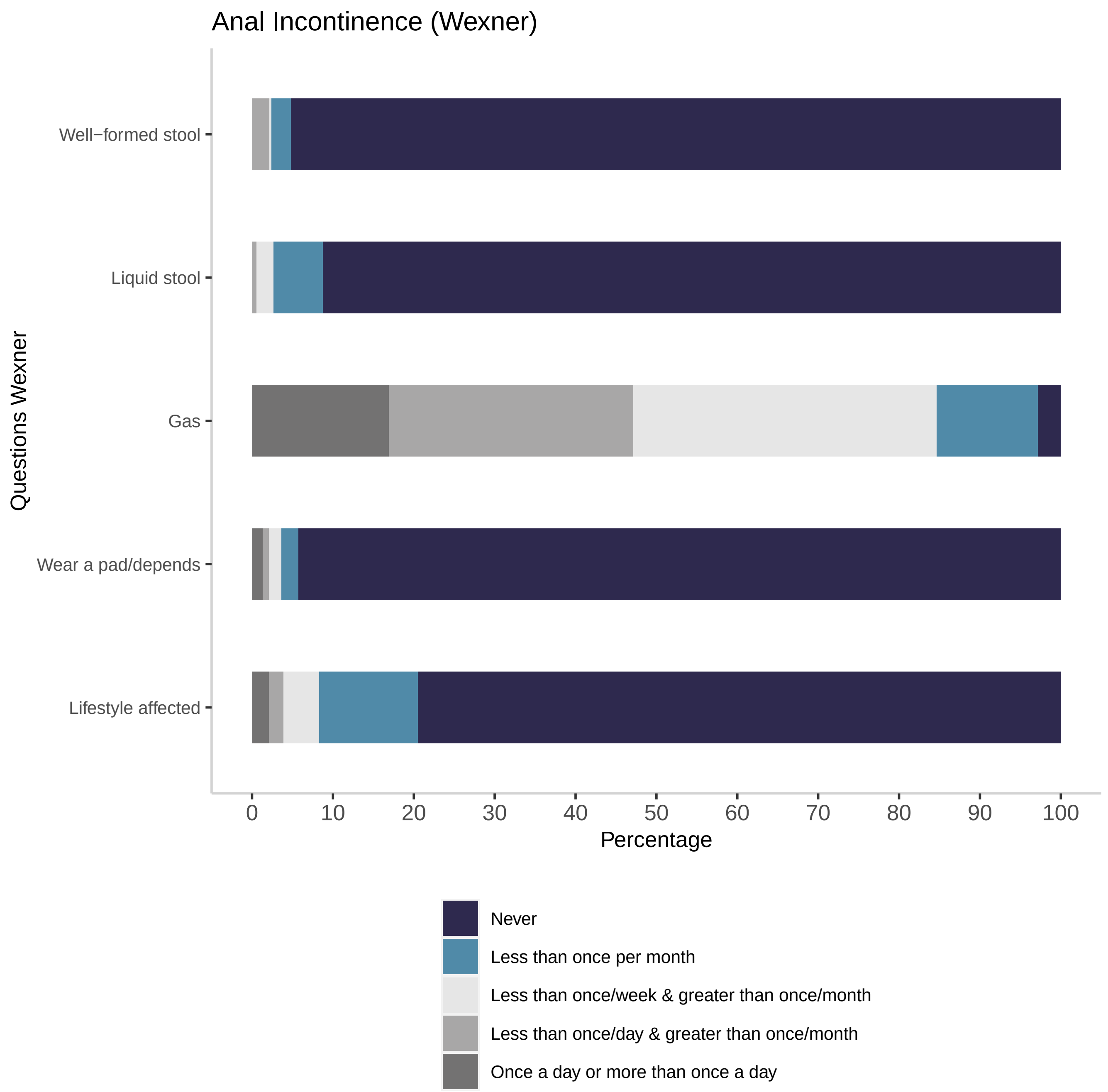

Anal Incontinence (Wexner)
Questions Wexner
Well−formed stool
Liquid stool
Gas
Wear a pad/depends
Lifestyle affected
0
10
20
30
40
50
60
70
80
90
100
Percentage
Never
Less than once per month
Less than once/week & greater than once/month
Less than once/day & greater than once/month
Once a day or more than once a day

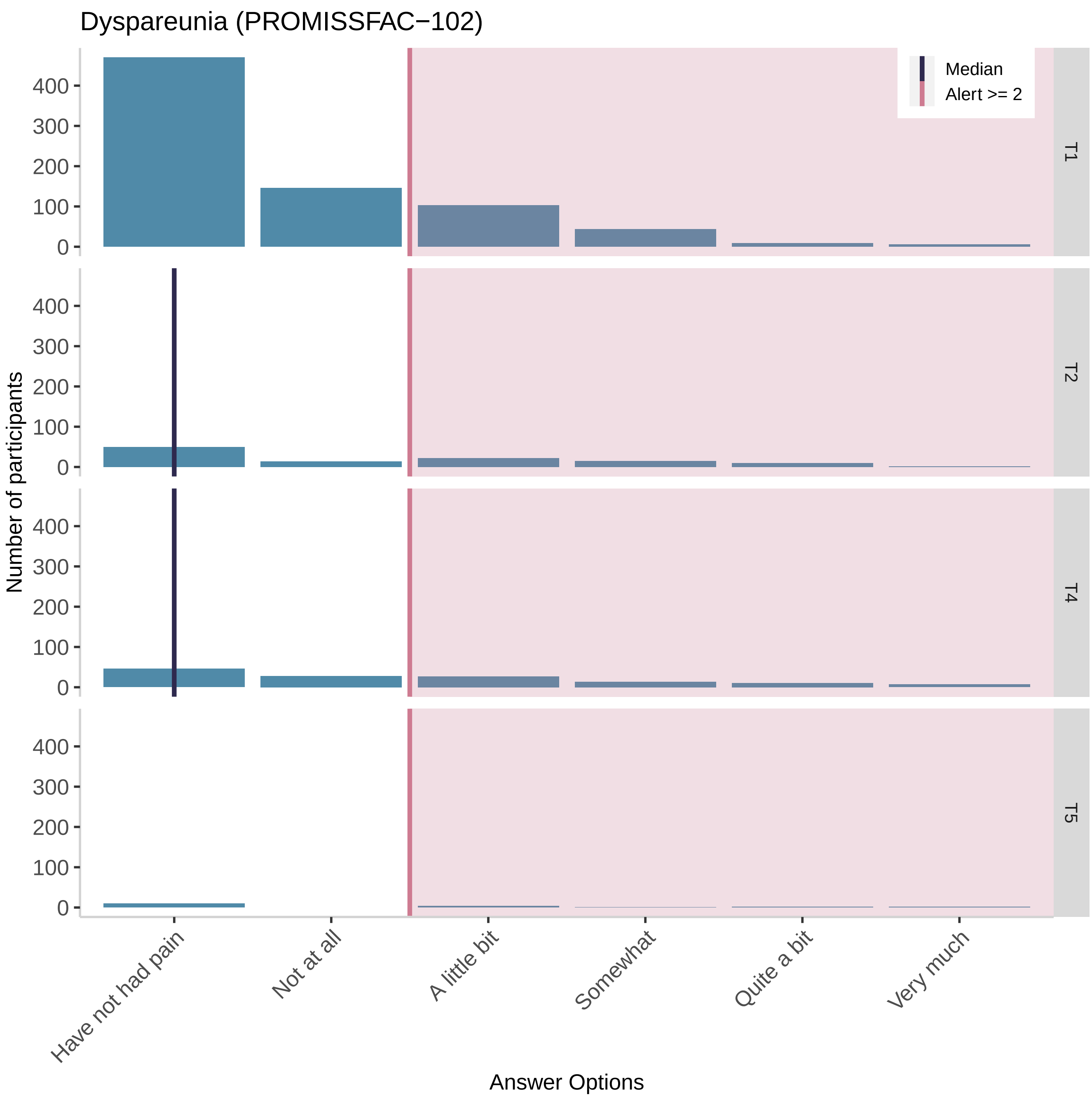

Dyspareunia (PROMISSFAC−102)
Median
Alert >= 2
Number of participants
400
300
200
100
0
T1
T2
T4
T5
Have not had pain
Not at all
A little bit
Somewhat
Quite a bit
Very much
Answer Options

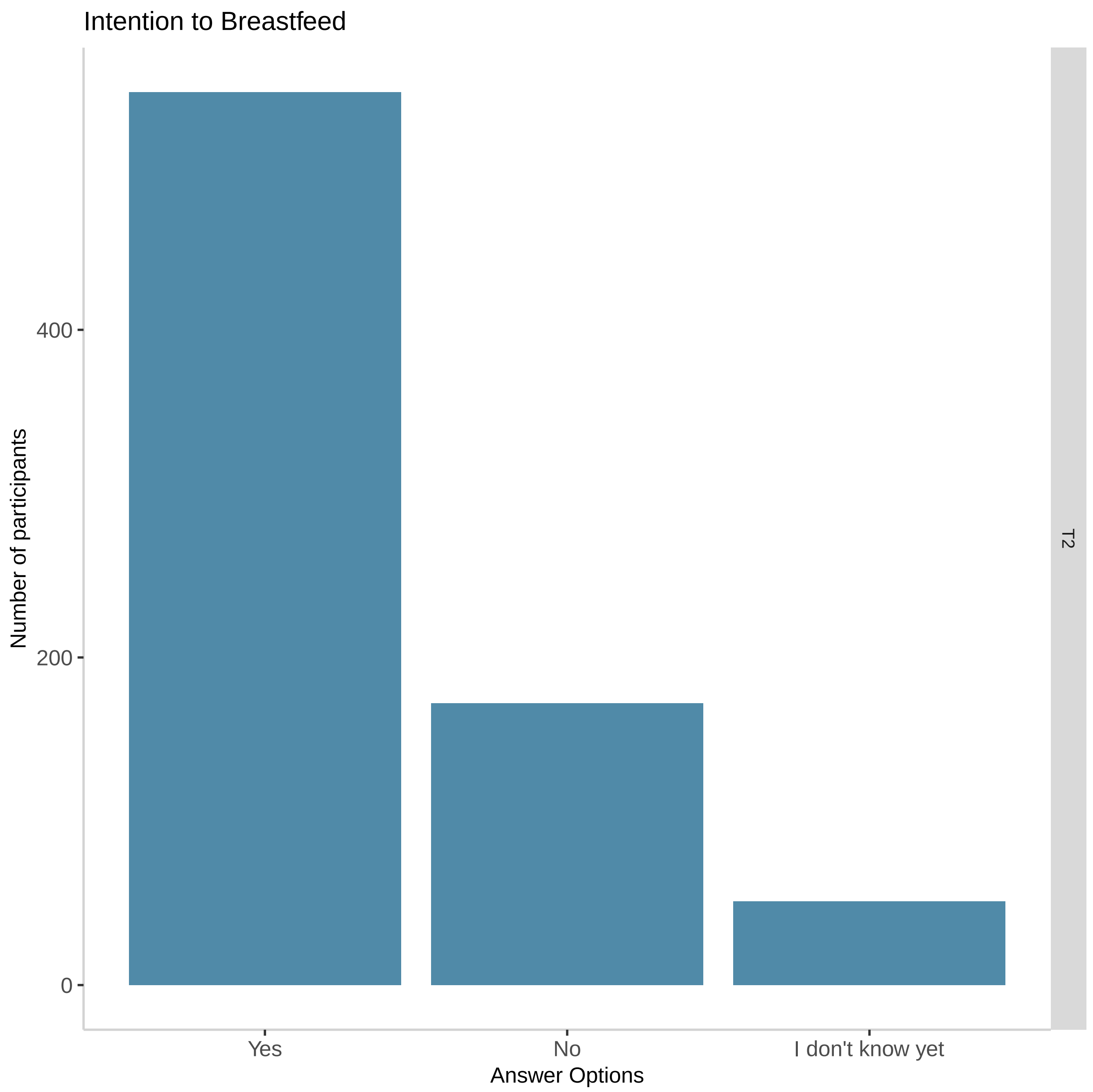
Intention to Breastfeed
Number of participants
400
200
0
Yes
No
I don't know yet
Answer Options
T2

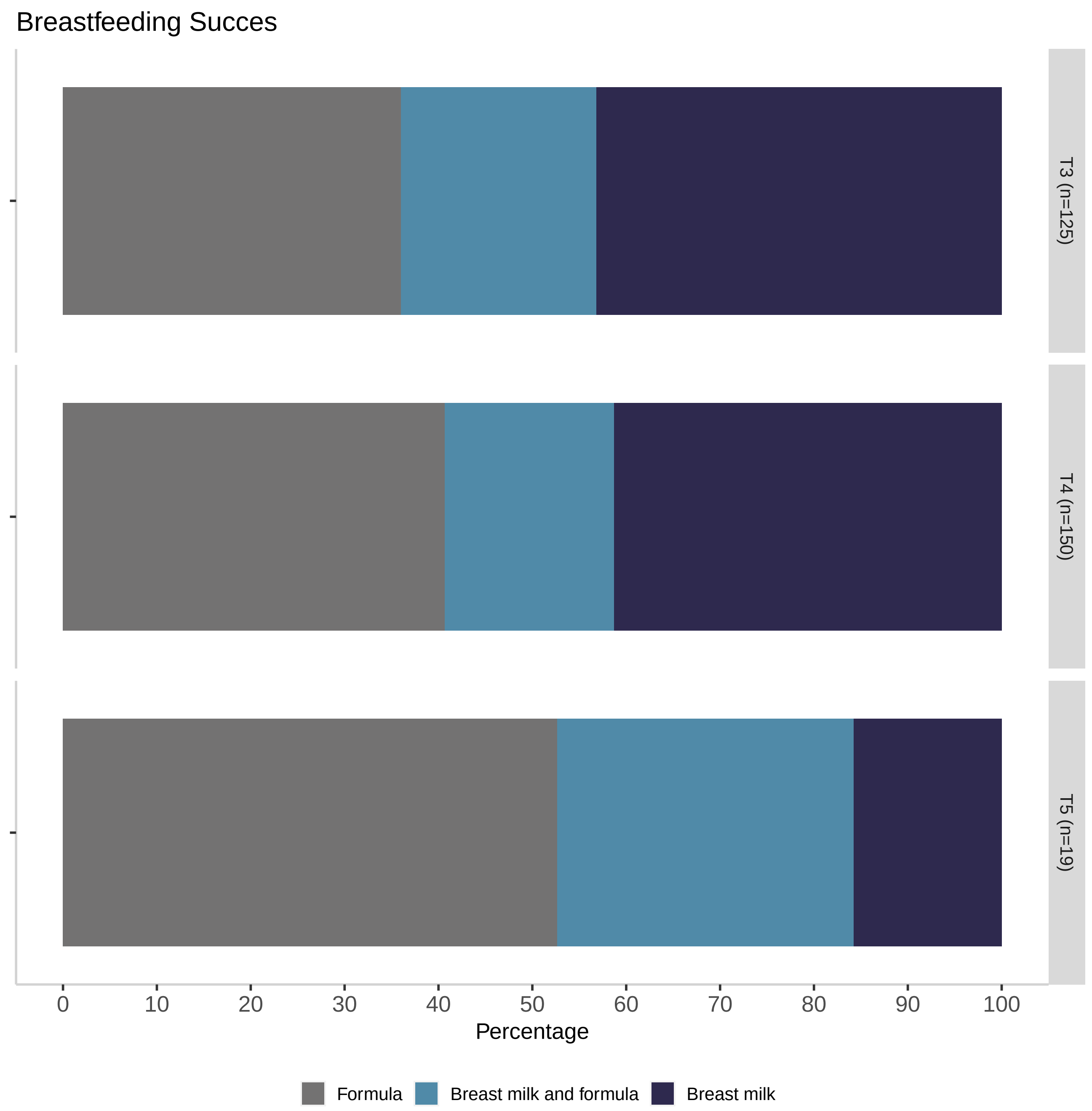
Breastfeeding Succes
T3 (n=125)
T4 (n=150)
T5 (n=19)
0
10
20
30
40
50
60
70
80
90
100
Percentage
Formula
Breast milk and formula
Breast milk

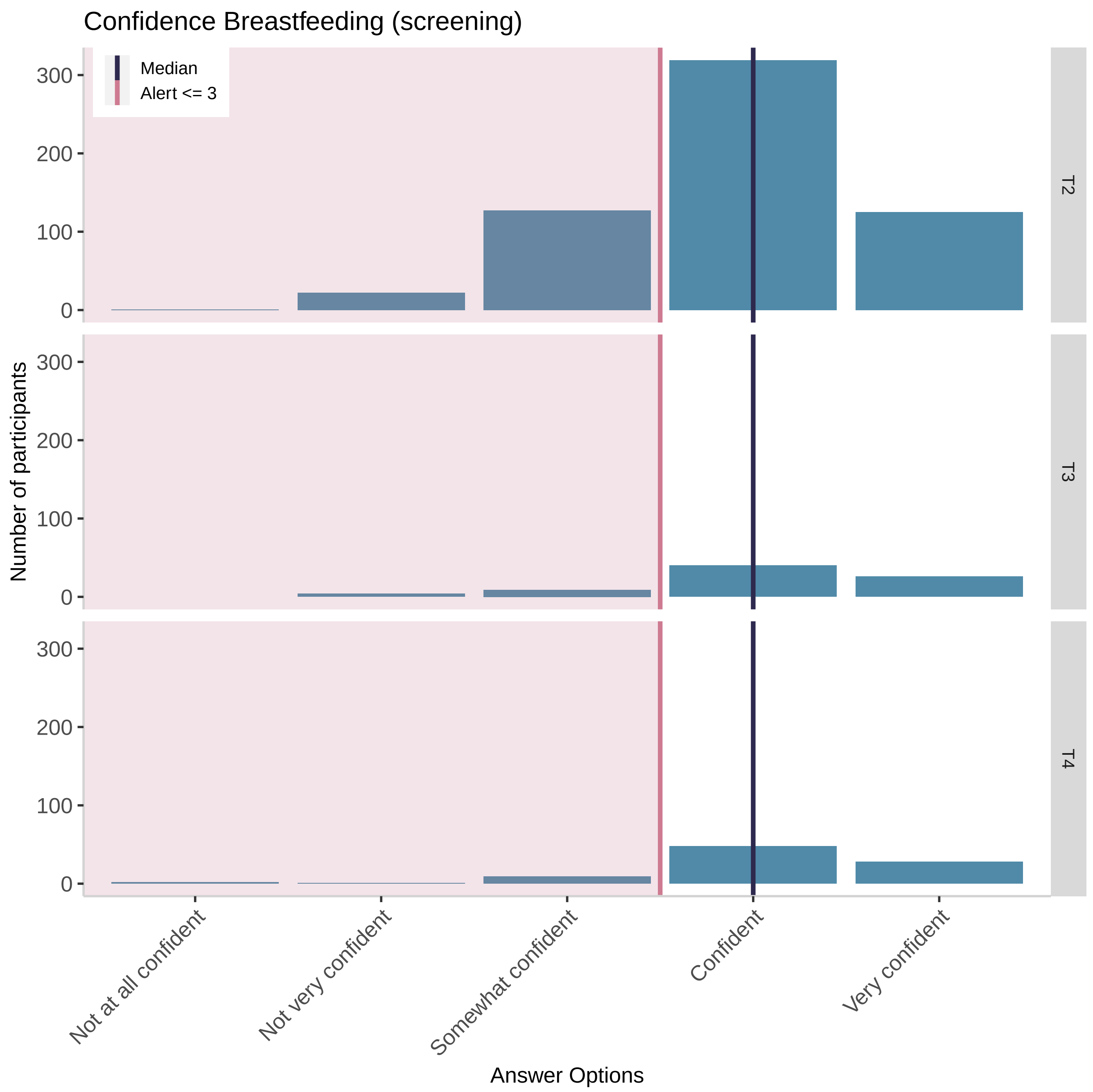

Confidence Breastfeeding (screening)
Median
Alert <= 3
Number of participants
300
200
100
0
T2
T3
T4
Not at all confident
Not very confident
Somewhat confident
Confident
Very confident
Answer Options

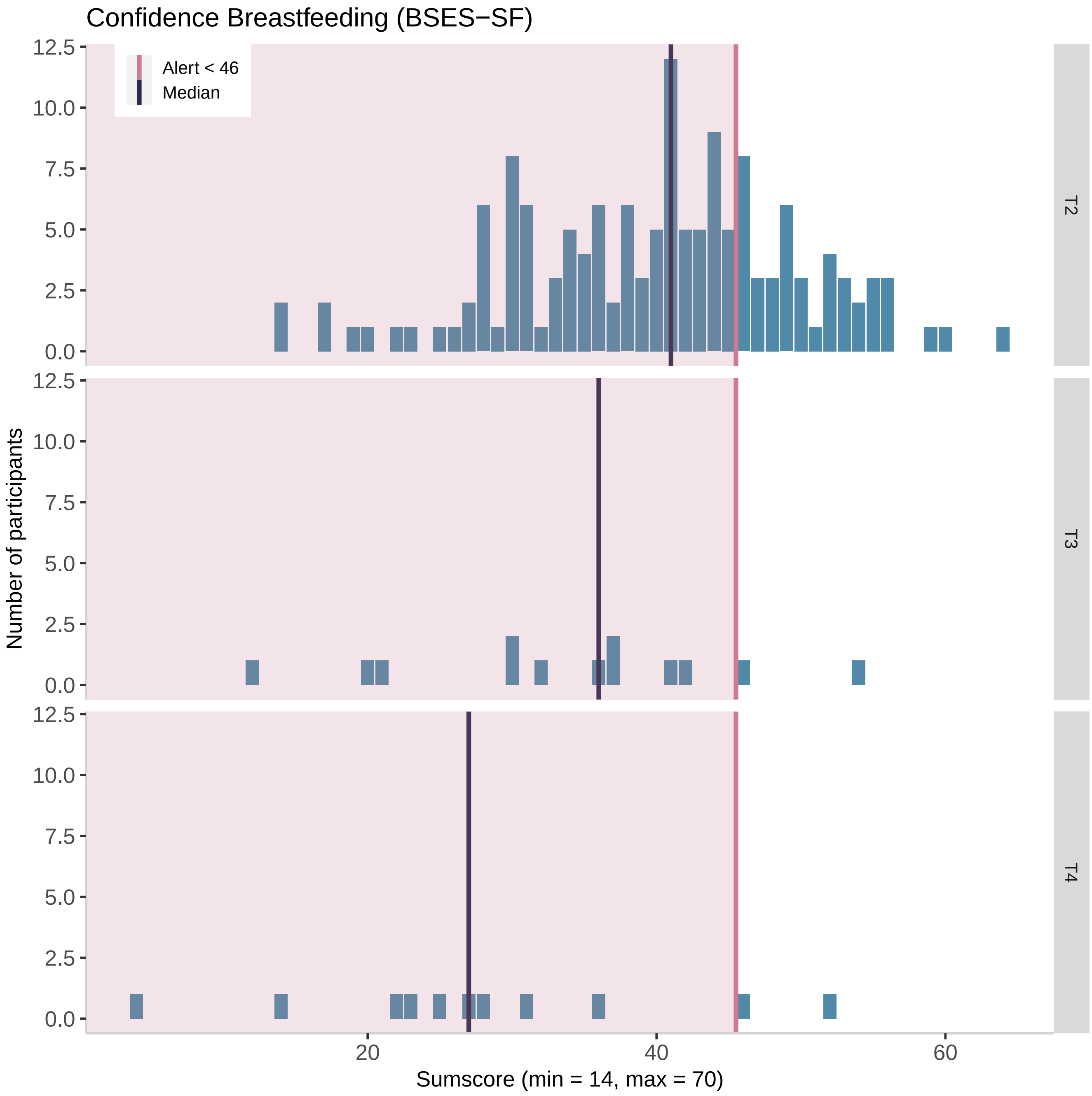
Confidence Breastfeeding (BSES−SF)
Alert < 46
Median
Number of participants
0.0
2.5
5.0
7.5
10.0
12.5
T2
T3
T4
20
40
60
Sumscore (min = 14, max = 70)

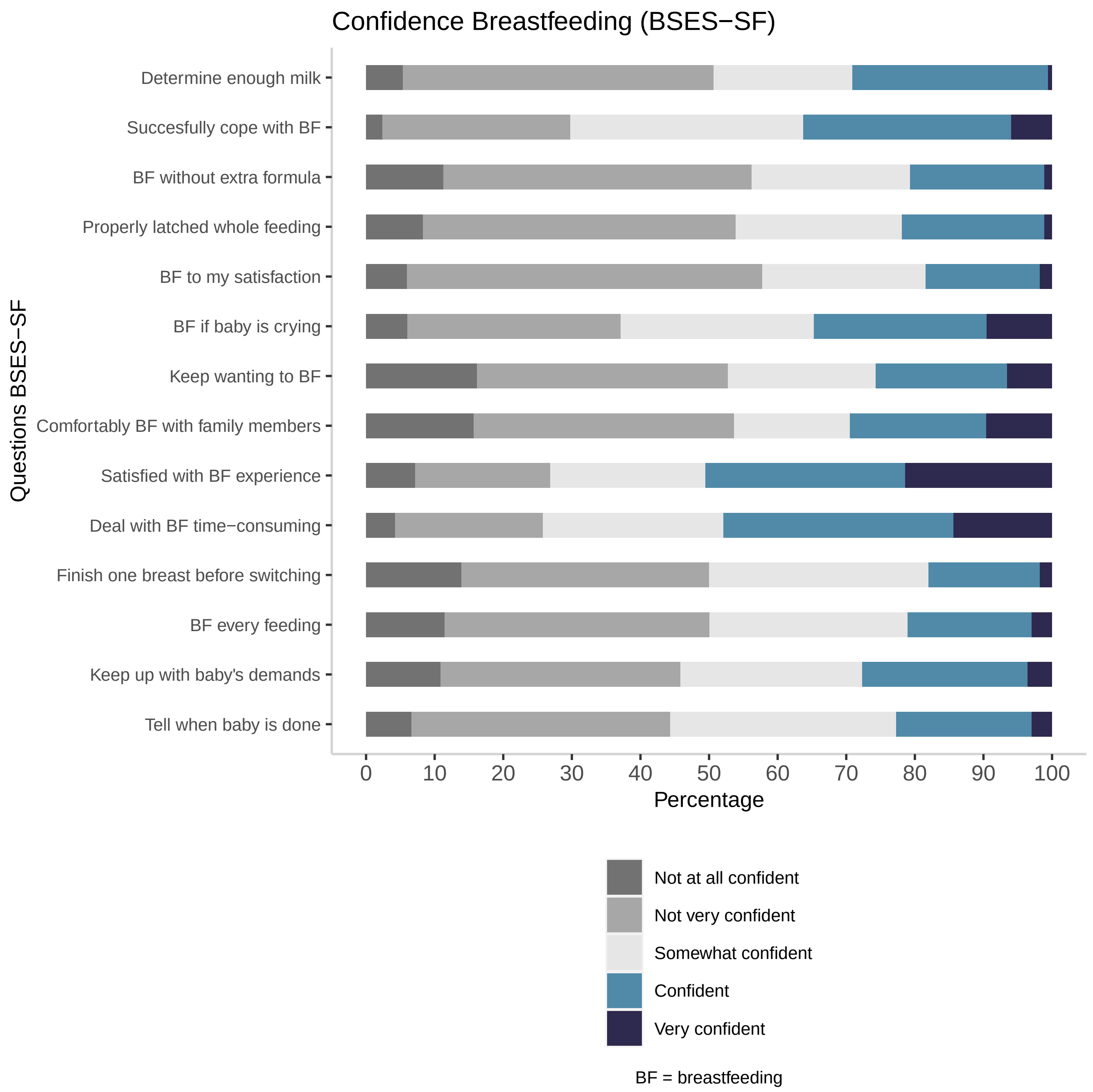
Confidence Breastfeeding (BSES−SF)
Questions BSES−SF
Determine enough milk
Succesfully cope with BF
BF without extra formula
Properly latched whole feeding
BF to my satisfaction
BF if baby is crying
Keep wanting to BF
Comfortably BF with family members
Satisfied with BF experience
Deal with BF time−consuming
Finish one breast before switching
BF every feeding
Keep up with baby's demands
Tell when baby is done
0 10 20 30 40 50 60 70 80 90 100
Percentage
Not at all confident
Not very confident
Somewhat confident
Confident
Very confident
BF = breastfeeding

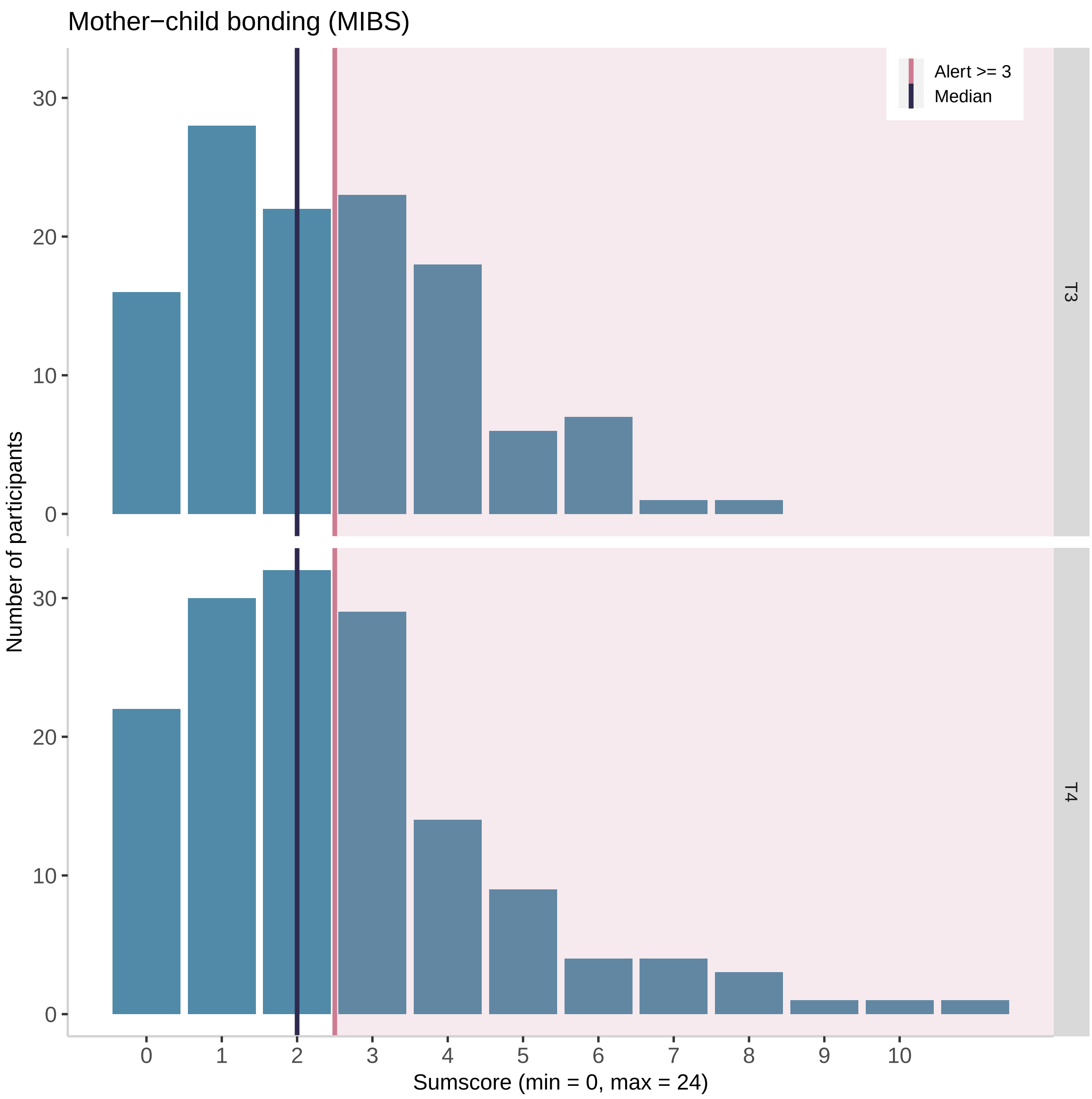
Mother−child bonding (MIBS)
Alert >= 3
Median
T3
T4
Number of participants
0
10
20
30
0
1
2
3
4
5
6
7
8
9
10
Sumscore (min = 0, max = 24)

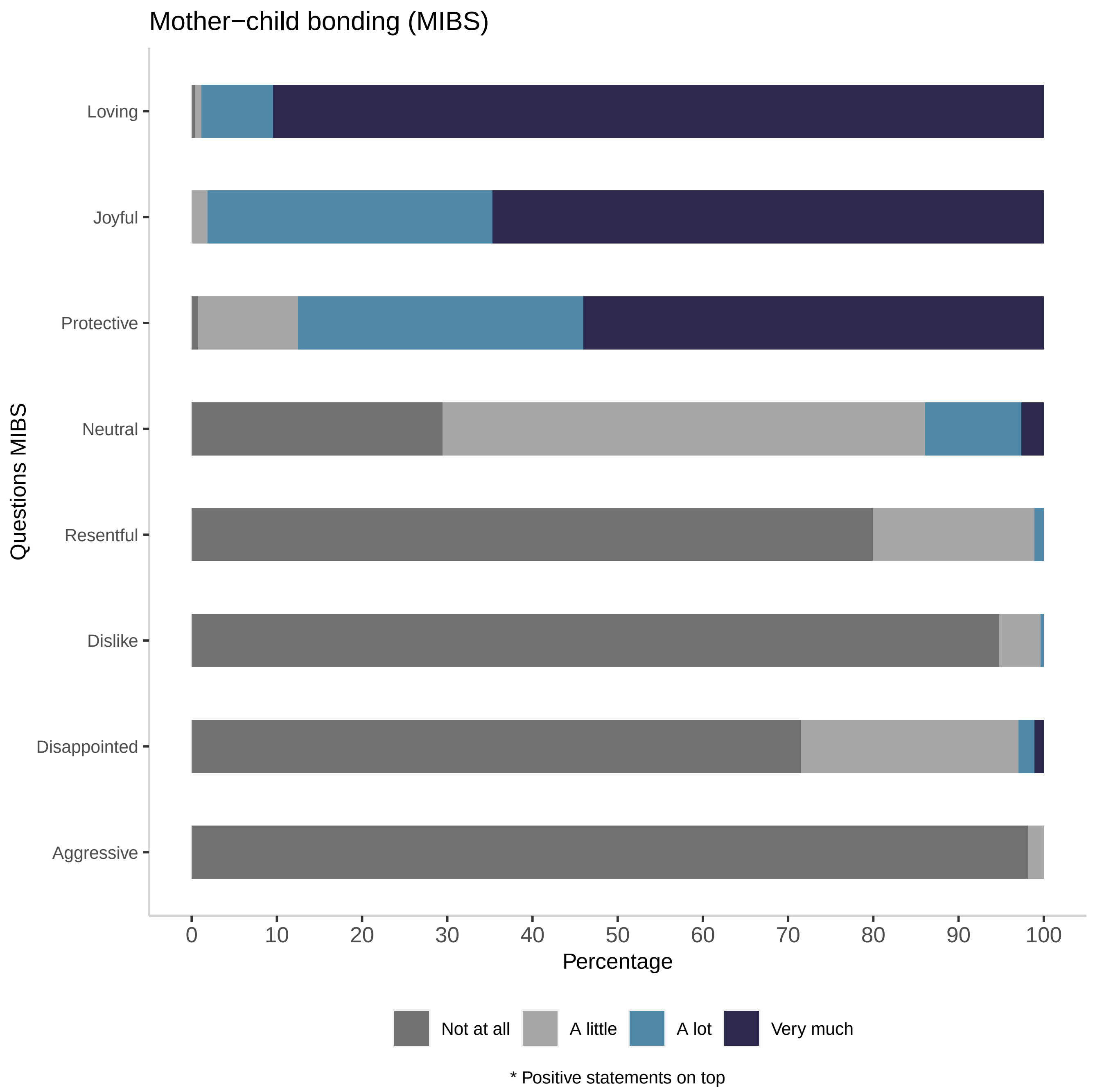

Mother−child bonding (MIBS)
Questions MIBS
Loving
Joyful
Protective
Neutral
Resentful
Dislike
Disappointed
Aggressive
0
10
20
30
40
50
60
70
80
90
100
Percentage
Not at all
A little
A lot
Very much
* Positive statements on top

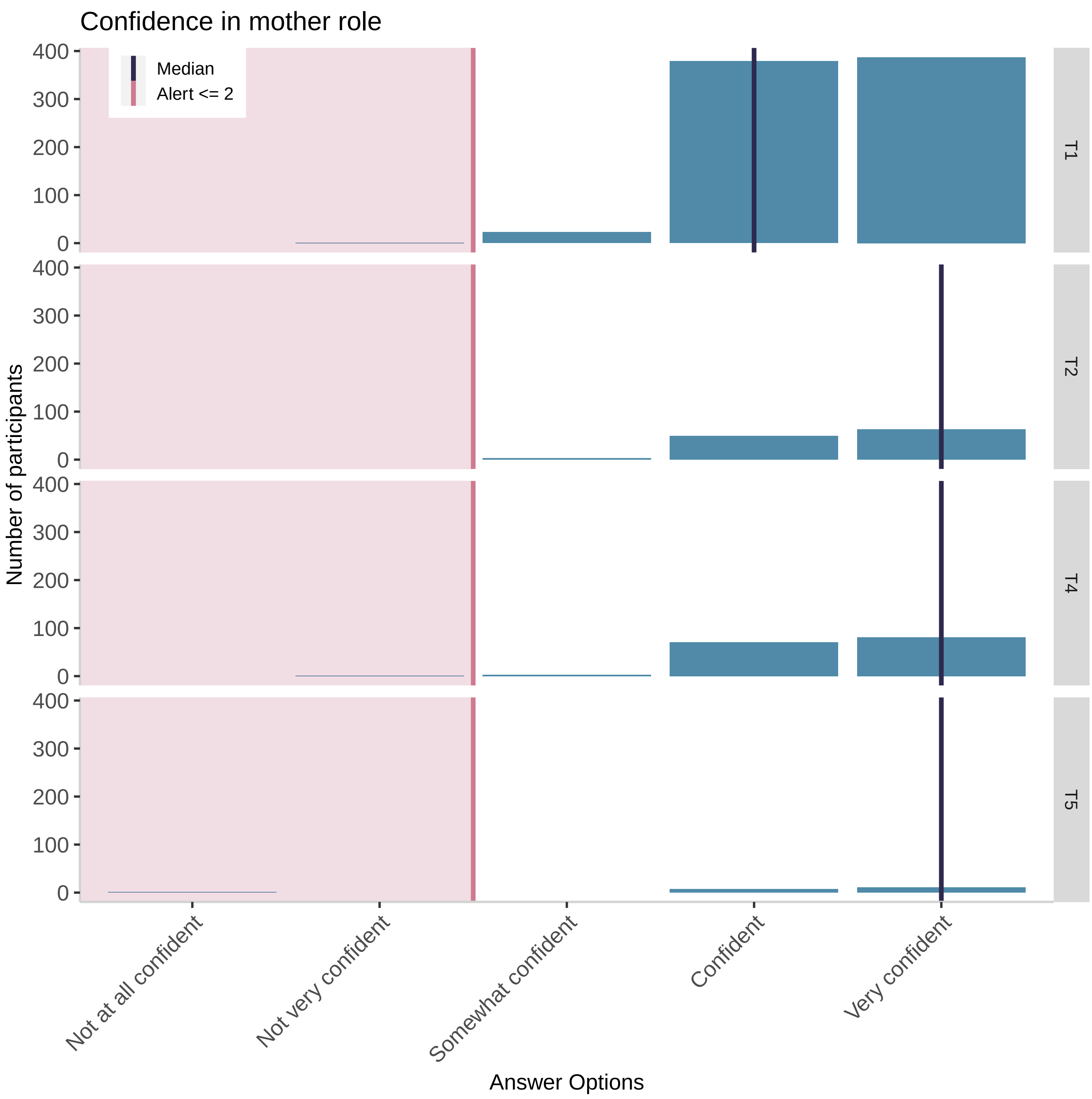

Confidence in mother role
Median
Alert <= 2
400
300
200
100
0
T1
T2
T4
T5
Number of participants
Not at all confident
Not very confident
Somewhat confident
Confident
Very confident
Answer Options

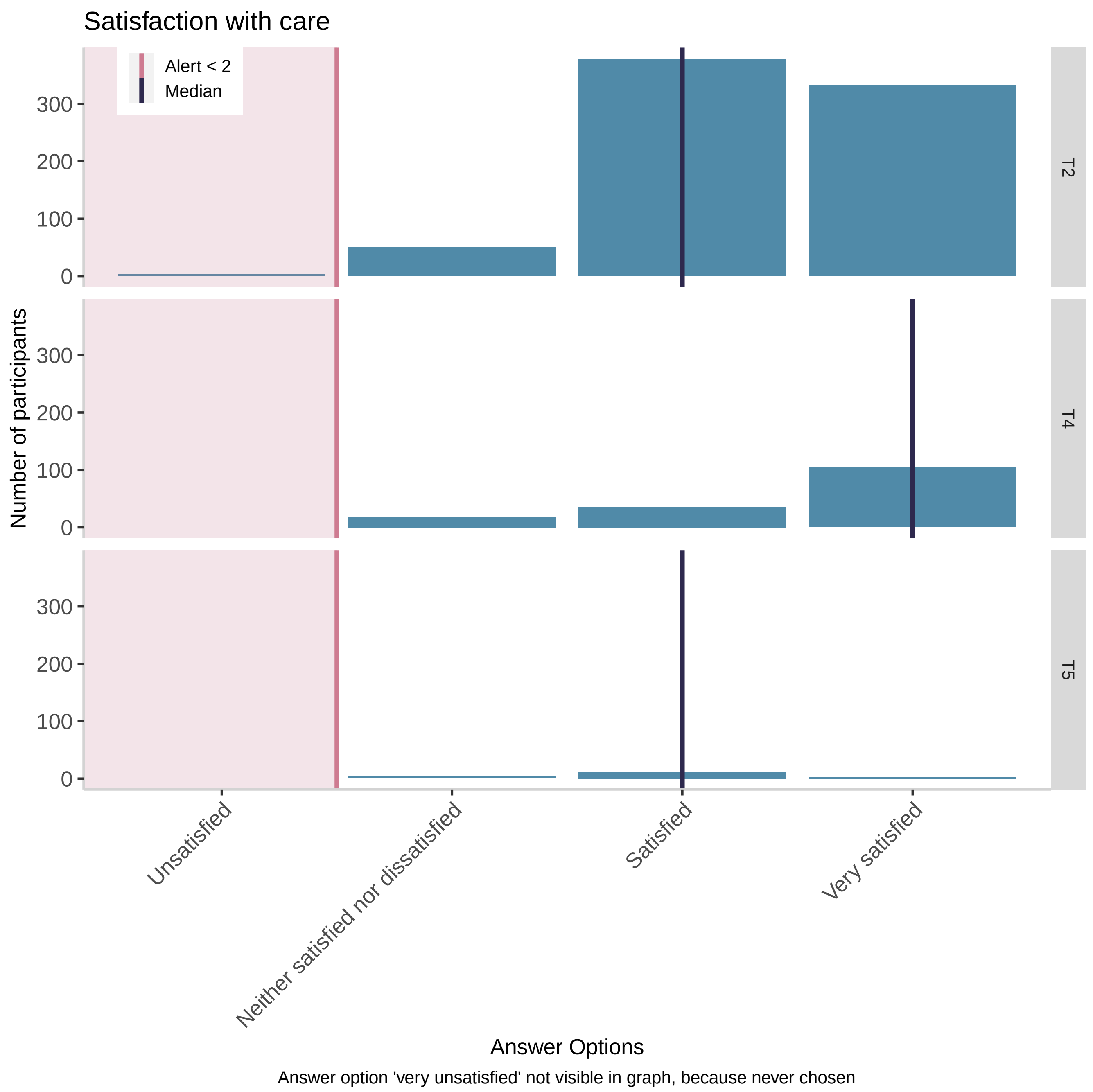
Satisfaction with care
Alert < 2
Median
Number of participants
300
200
100
0
T2
T4
T5
Unsatisfied
Neither satisfied nor dissatisfied
Satisfied
Very satisfied
Answer Options
Answer option 'very unsatisfied' not visible in graph, because never chosen

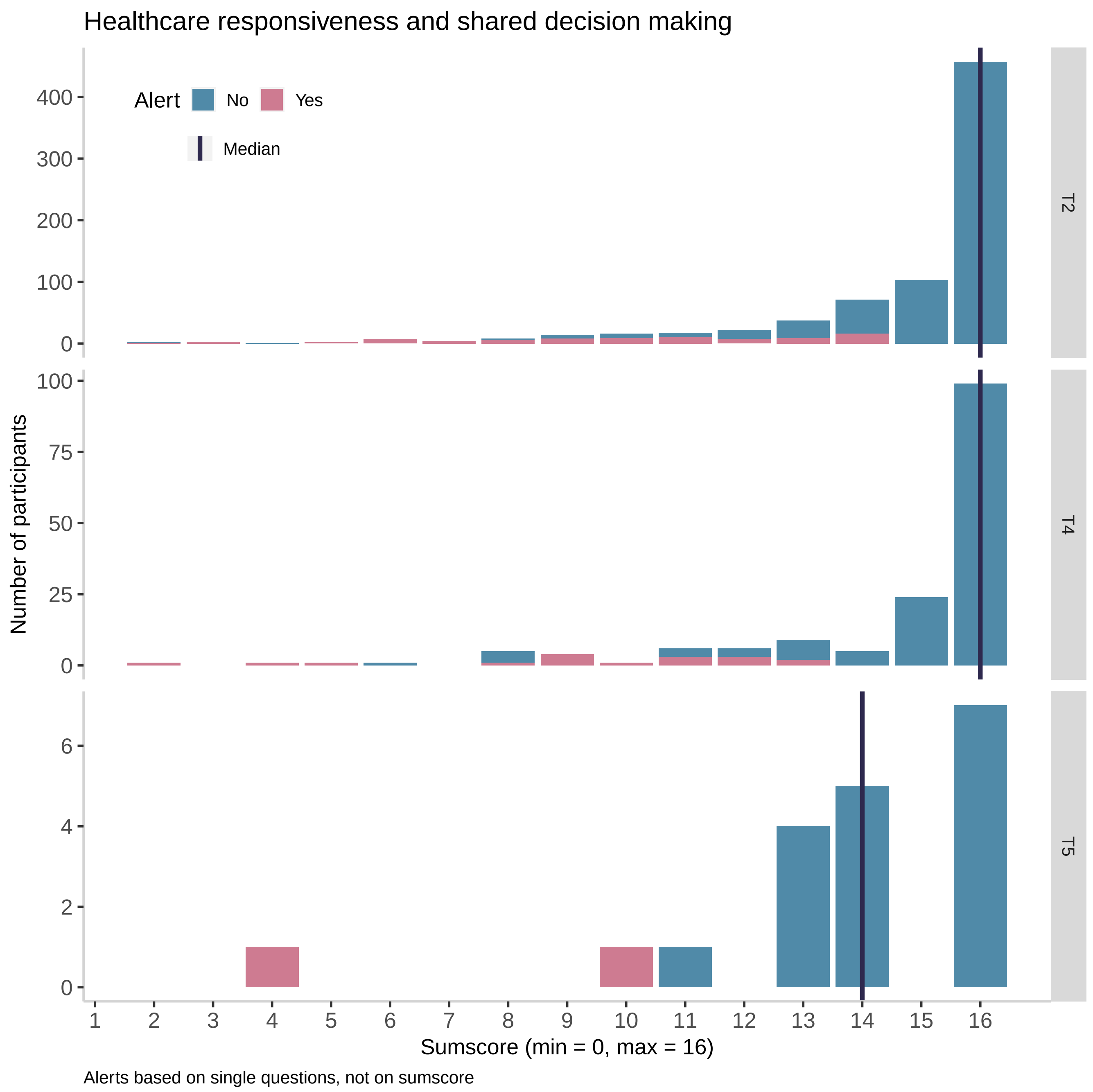

Healthcare responsiveness and shared decision making
Alert
No
Yes
Median
Number of participants
T2
T4
T5
Sumscore (min = 0, max = 16)
Alerts based on single questions, not on sumscore

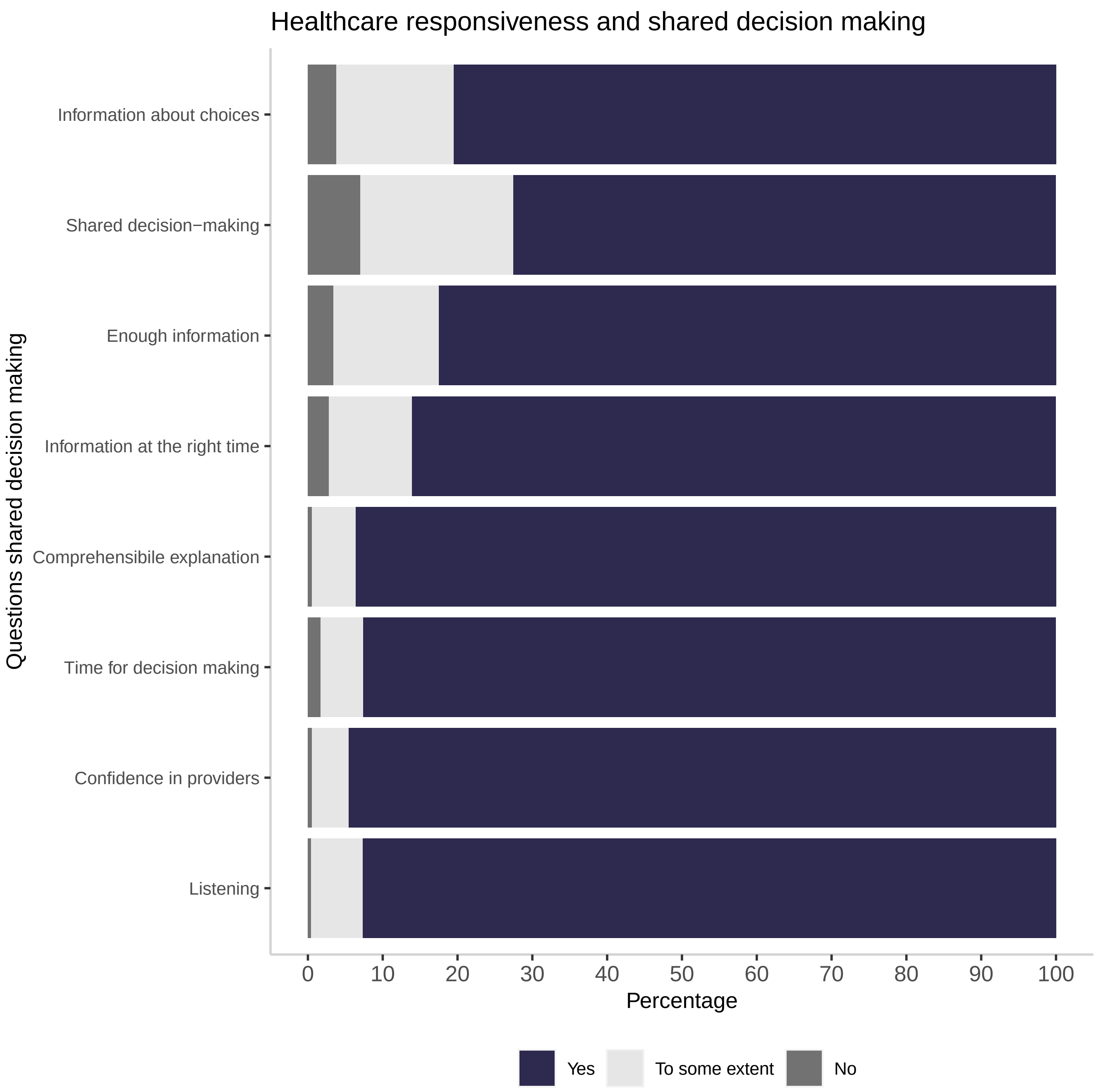
Healthcare responsiveness and shared decision making
Questions shared decision making
Information about choices
Shared decision−making
Enough information
Information at the right time
Comprehensibile explanation
Time for decision making
Confidence in providers
Listening
0
10
20
30
40
50
60
70
80
90
100
Percentage
Yes
To some extent
No

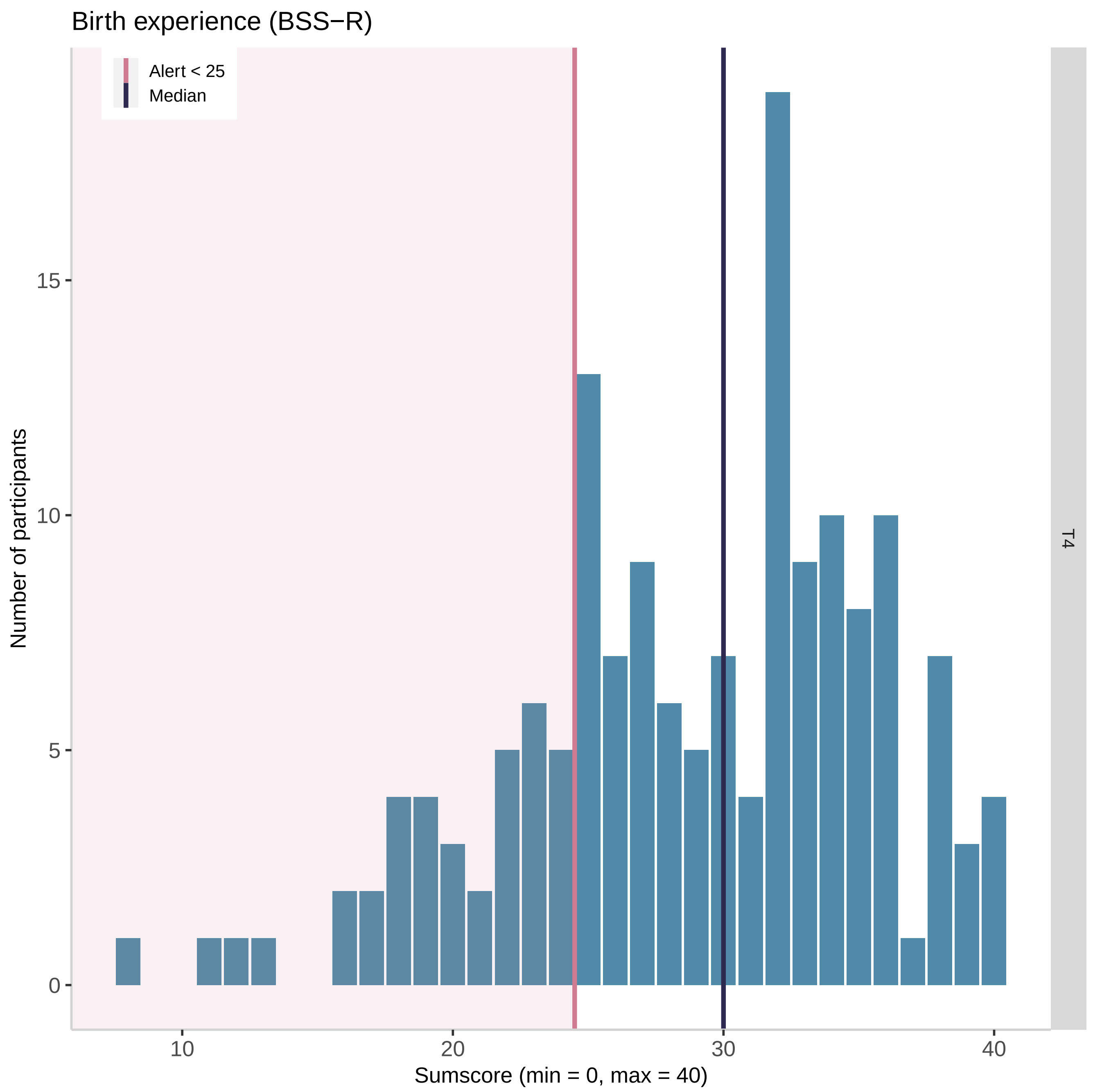

Birth experience (BSS−R)
Alert < 25
Median
Number of participants
0
5
10
15
10
20
30
40
Sumscore (min = 0, max = 40)
T4

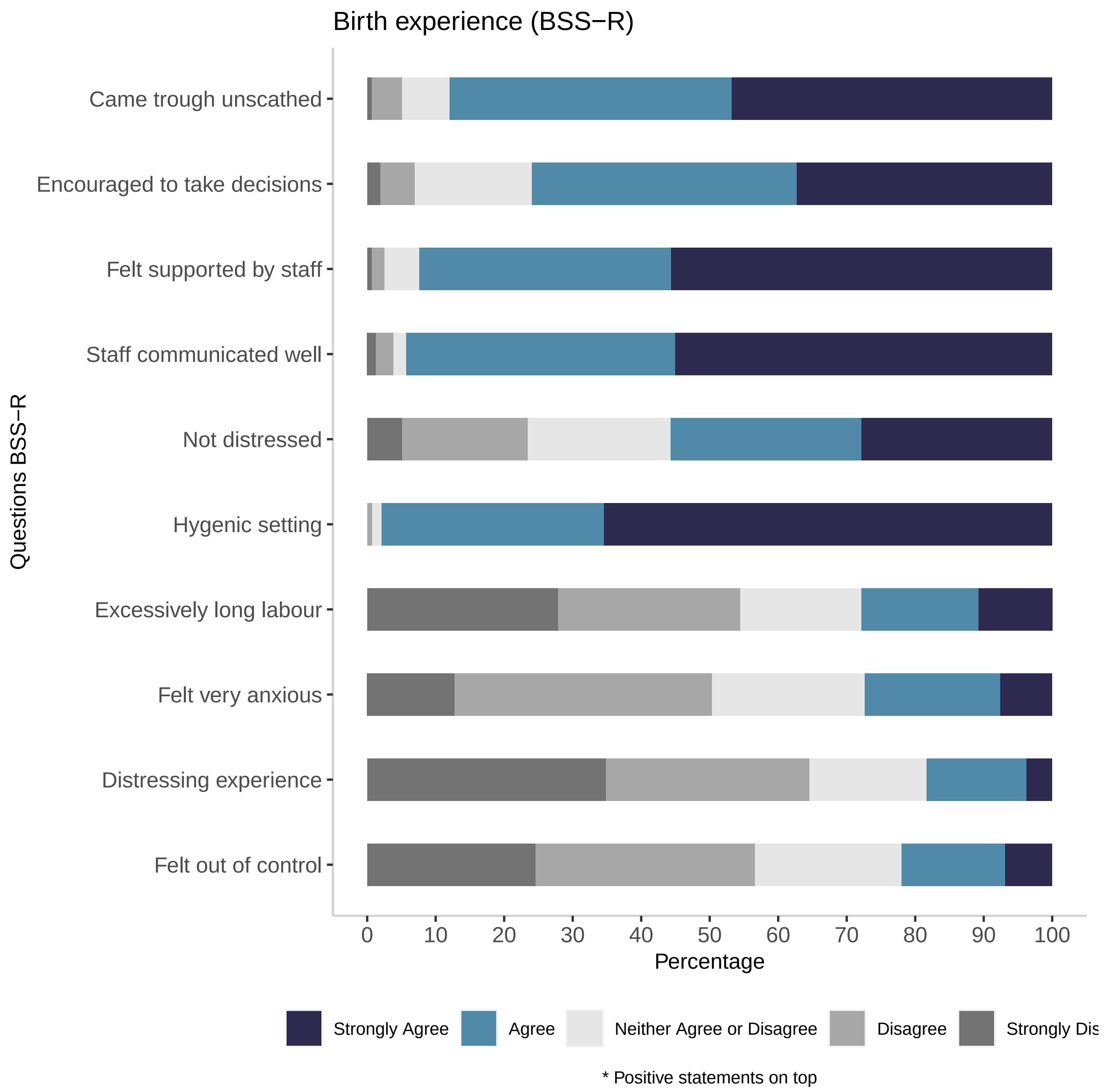
Birth experience (BSS−R)
Questions BSS−R
Came trough unscathed
Encouraged to take decisions
Felt supported by staff
Staff communicated well
Not distressed
Hygenic setting
Excessively long labour
Felt very anxious
Distressing experience
Felt out of control
0
10
20
30
40
50
60
70
80
90
100
Percentage
Strongly Agree
Agree
Neither Agree or Disagree
Disagree
Strongly Dis
* Positive statements on top

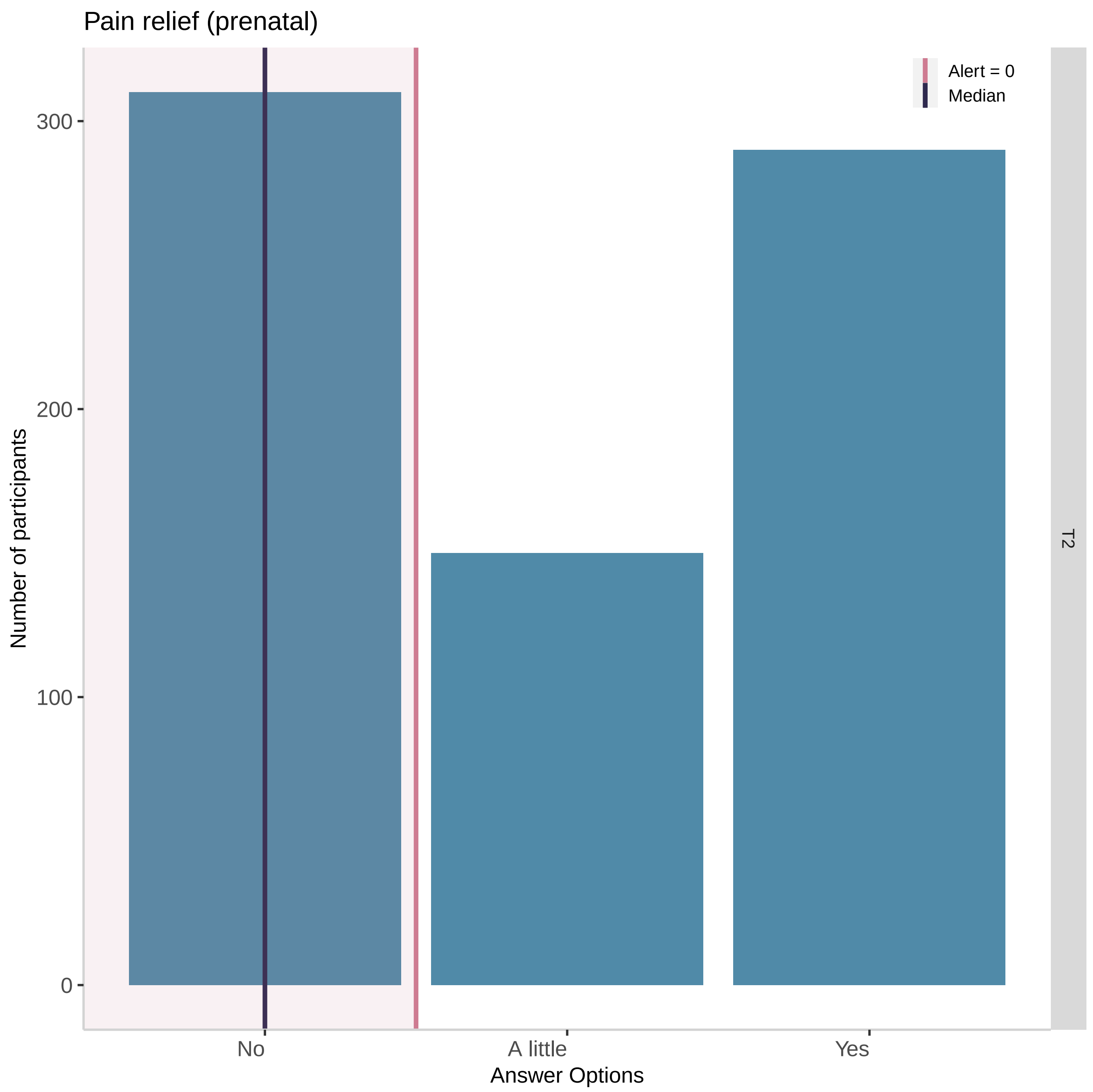

Pain relief (prenatal)
Alert = 0
Median
300
200
100
0
Number of participants
No
A little
Yes
Answer Options
T2

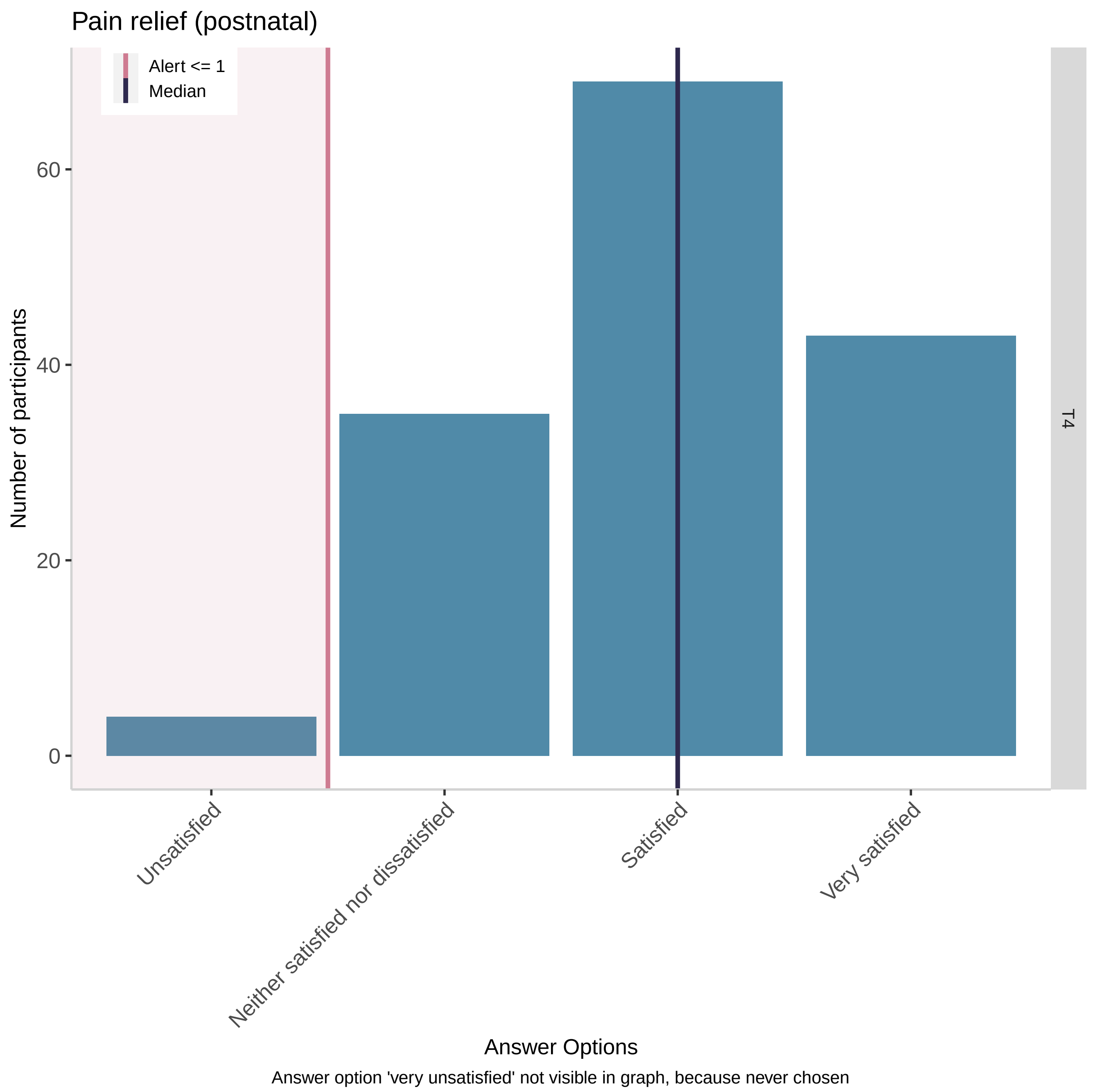
Pain relief (postnatal)
Alert <= 1
Median
Number of participants
0
20
40
60
T4
Unsatisfied
Neither satisfied nor dissatisfied
Satisfied
Very satisfied
Answer Options
Answer option 'very unsatisfied' not visible in graph, because never chosen

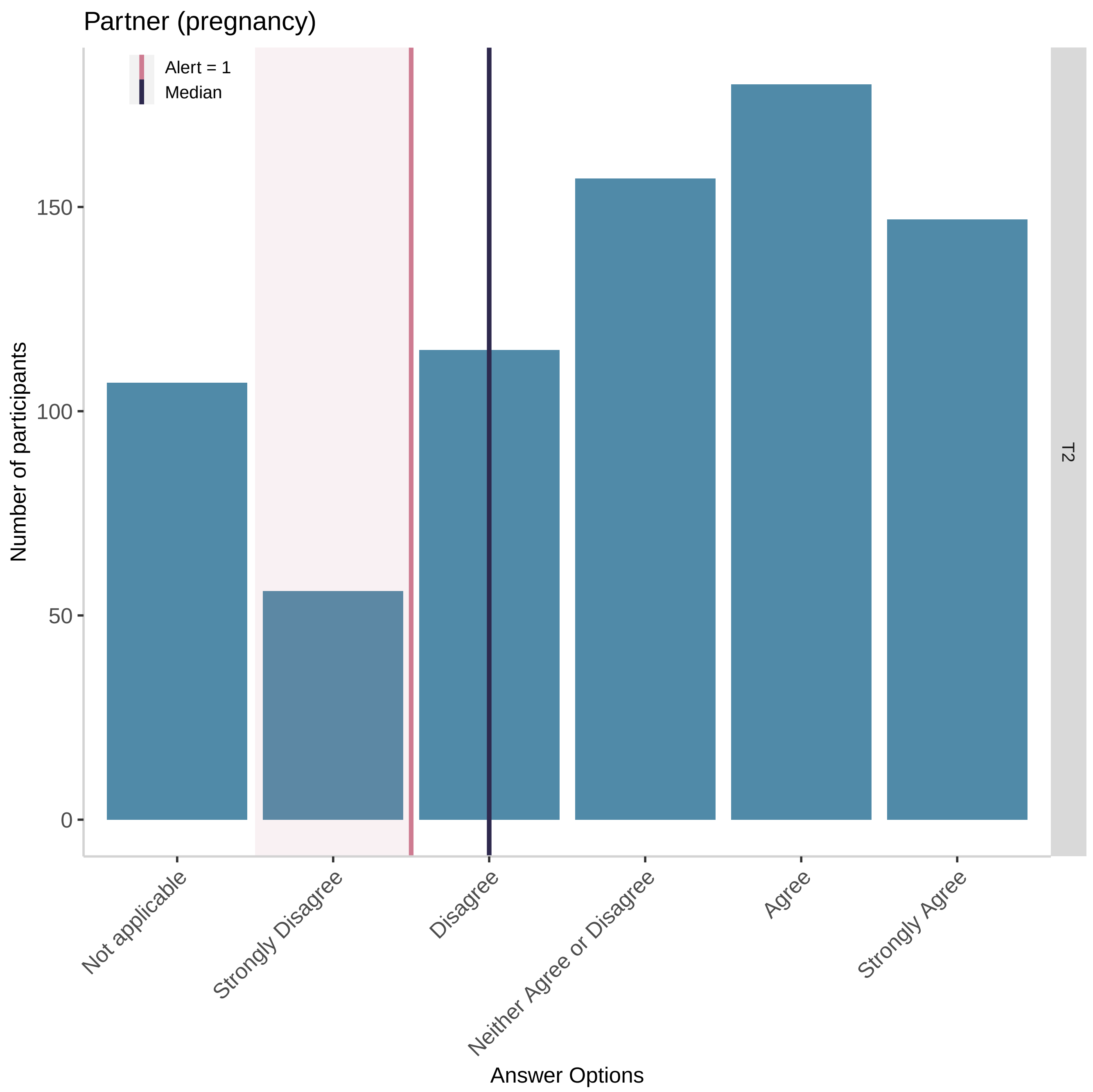
Partner (pregnancy)
Alert = 1
Median
Number of participants
150
100
50
0
T2
Not applicable
Strongly Disagree
Disagree
Neither Agree or Disagree
Agree
Strongly Agree
Answer Options

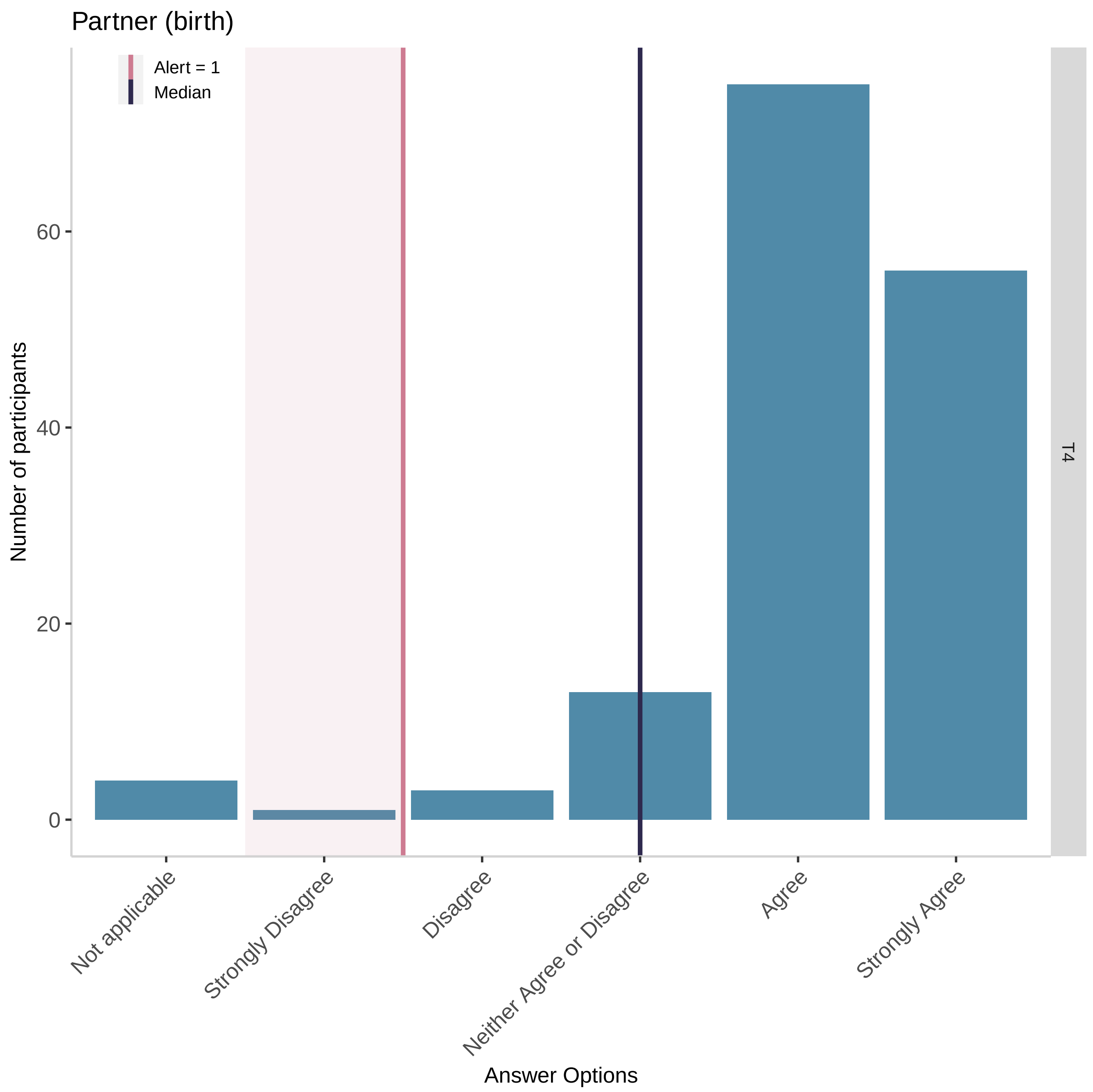

Partner (birth)
Alert = 1
Median
60
40
20
0
Number of participants
T4
Not applicable
Strongly Disagree
Disagree
Neither Agree or Disagree
Agree
Strongly Agree
Answer Options

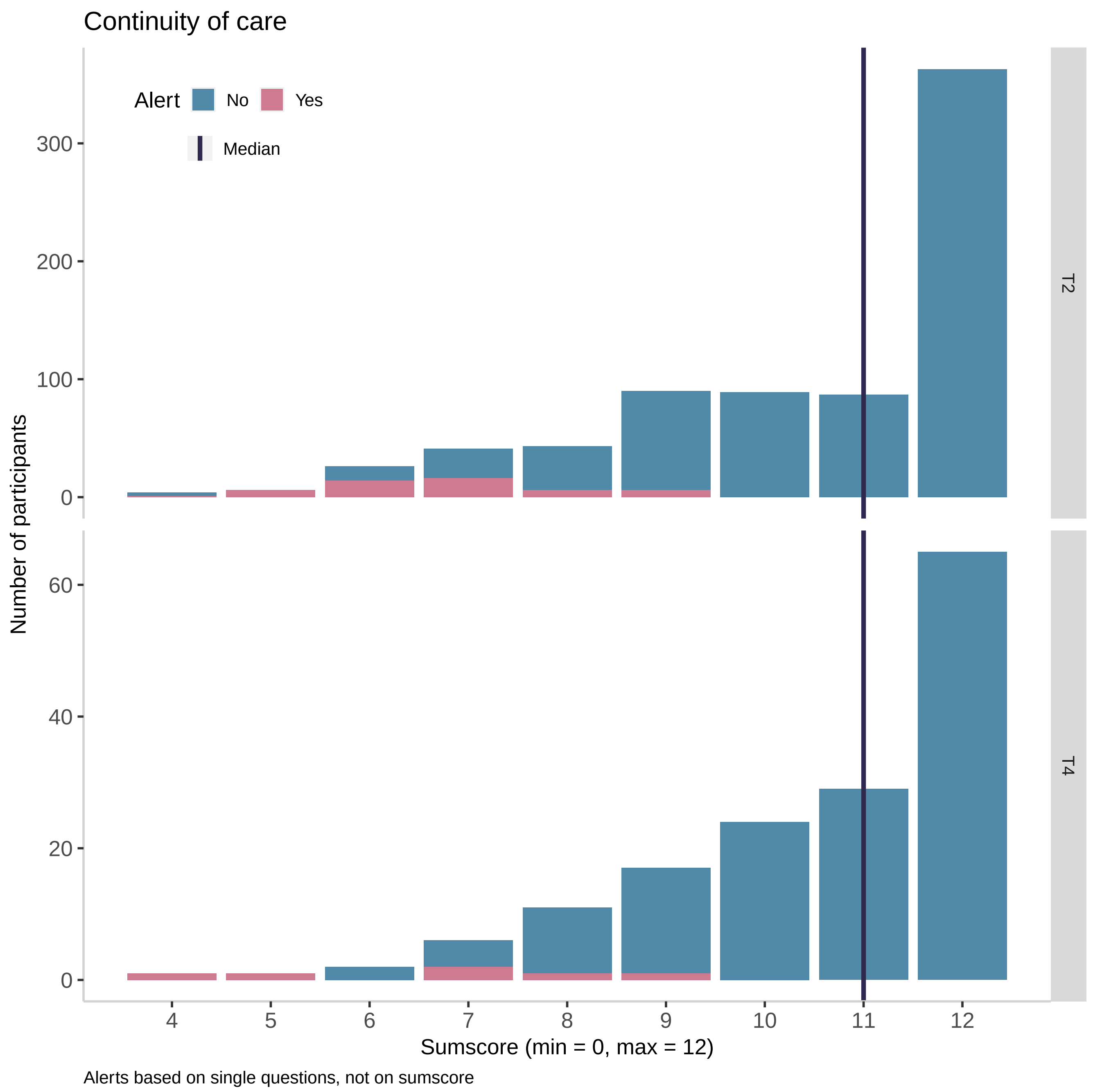
Continuity of care
Alert
No
Yes
Median
Number of participants
T2
T4
Sumscore (min = 0, max = 12)
Alerts based on single questions, not on sumscore

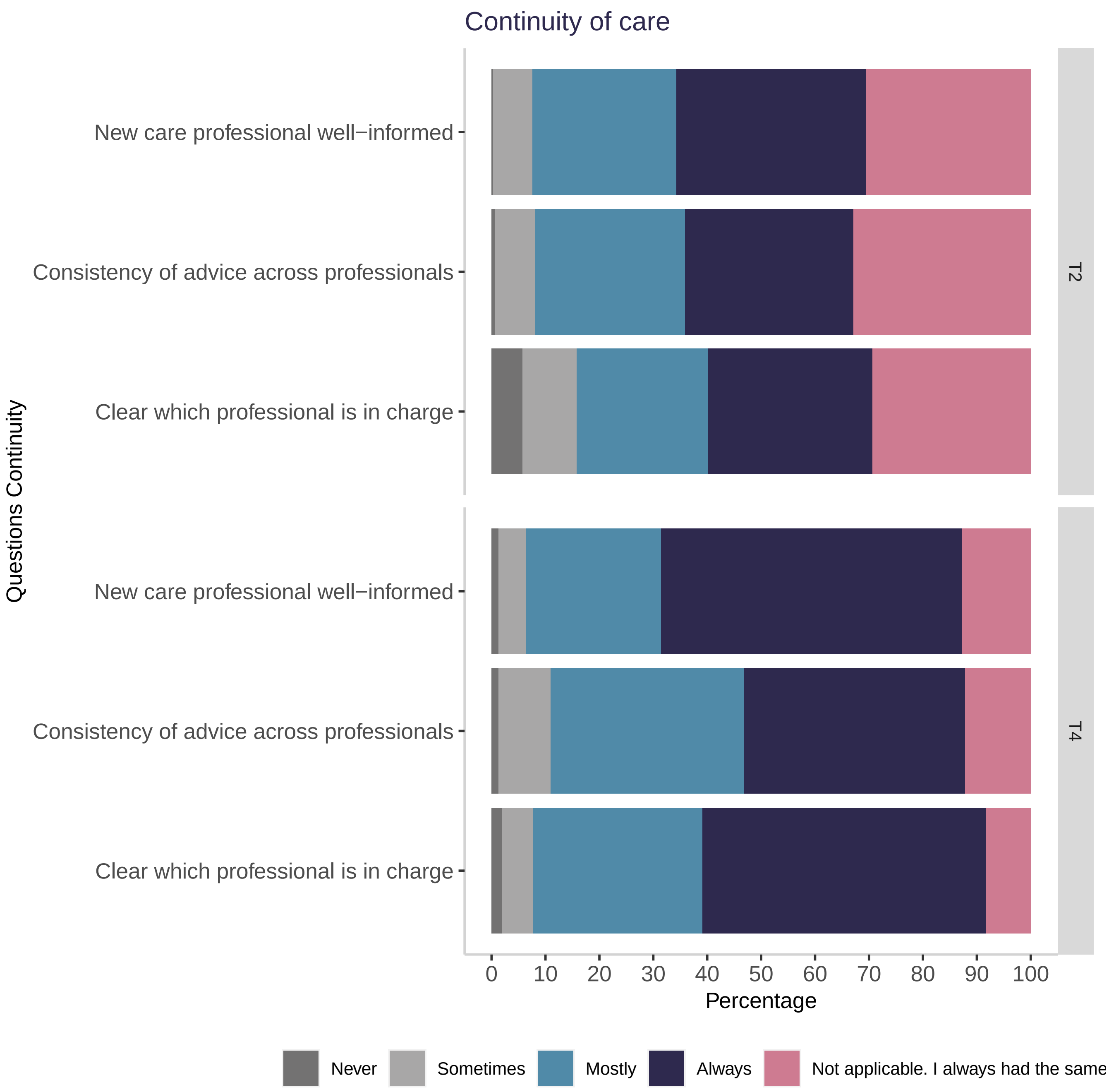
Continuity of care
New care professional well−informed
Consistency of advice across professionals
Clear which professional is in charge
T2
T4
Questions Continuity
0
10
20
30
40
50
60
70
80
90
100
Percentage
Never
Sometimes
Mostly
Always
Not applicable. I always had the same
