# Supplementary material for: Patient-Reported Outcome and Experience Measures in Perinatal Care to Guide Clinical Practice: Prospective Observational Study
Source: J Med Internet Res. 2022 Jul 5;24(7):e37725. doi: 10.2196/37725 (PMC9297146; doi:10.2196/37725)
Supplement: Multimedia Appendix 4 [file jmir_v24i7e37725_app4.pdf]

Quality of life (PROMIS−GH SF)

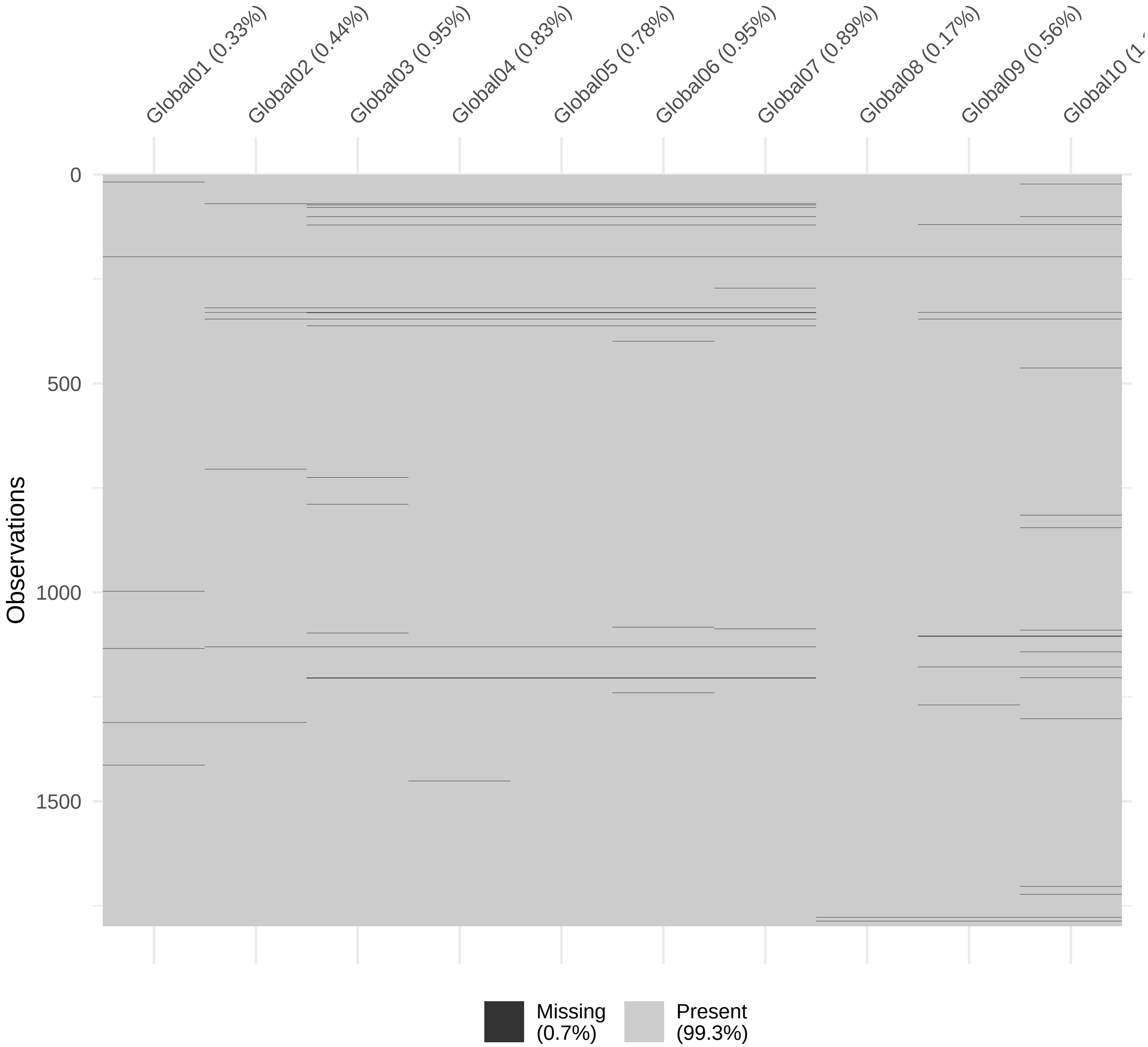

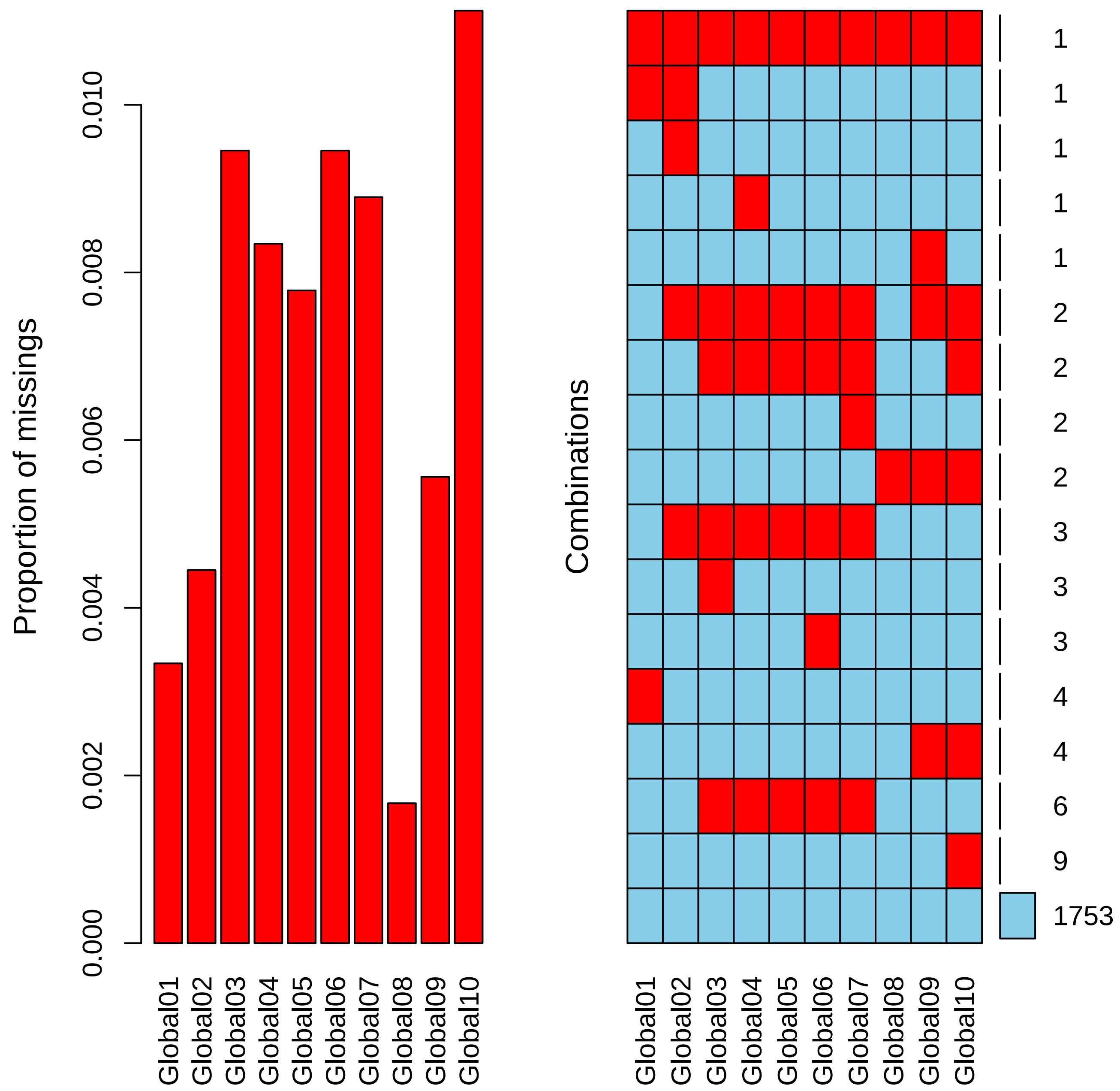

Proportion of missings
0.000
0.002
0.004
0.006
0.008
0.010
Global01
Global02
Global03
Global04
Global05
Global06
Global07
Global08
Global09
Global10
Combinations
1
1
1
1
1
2
2
2
2
3
3
3
4
4
6
9
1753

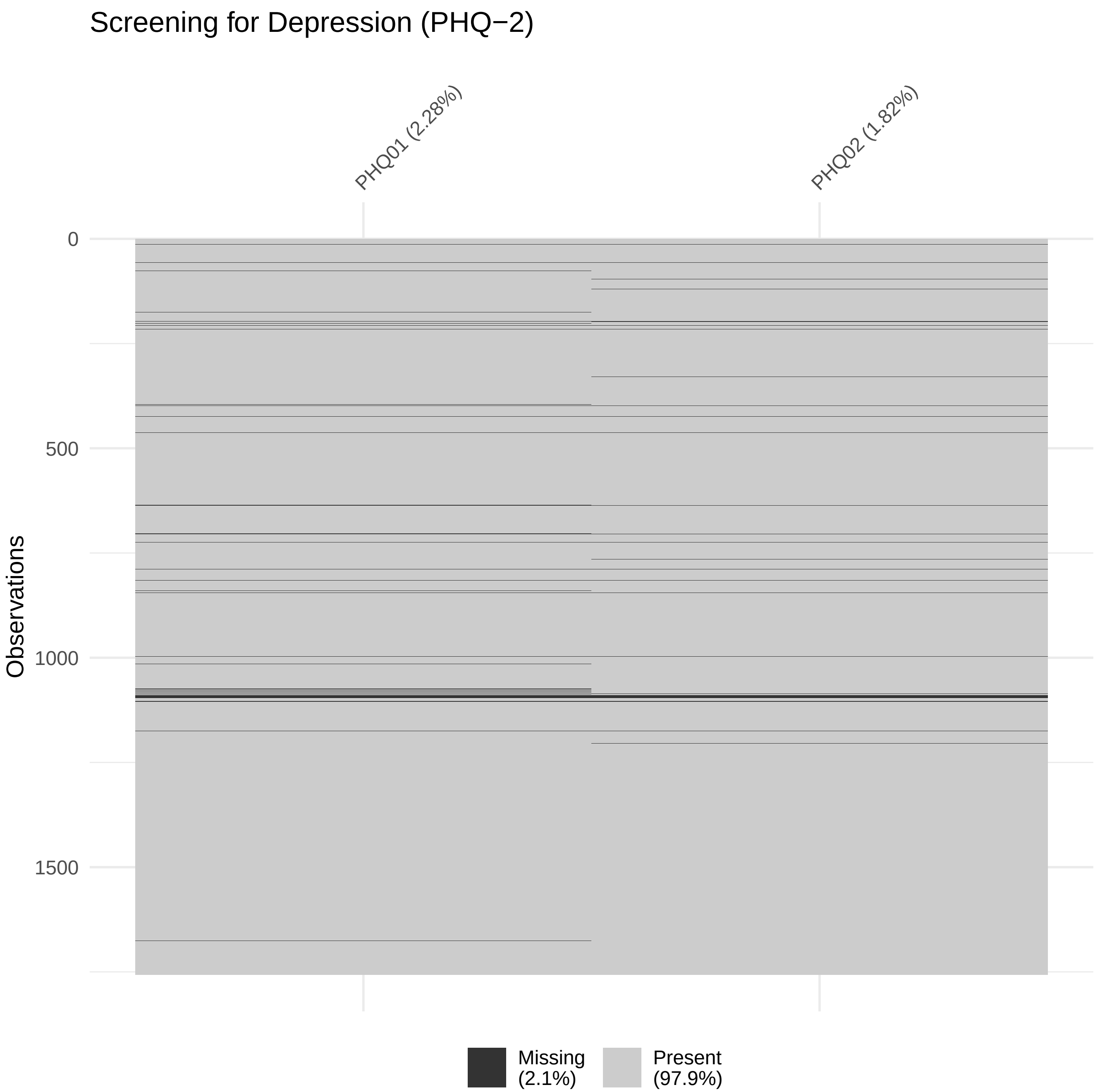

Screening for Depression (PHQ−2)
PHQ01 (2.28%)
PHQ02 (1.82%)
Observations
0
500
1000
1500
Missing (2.1%)
Present (97.9%)

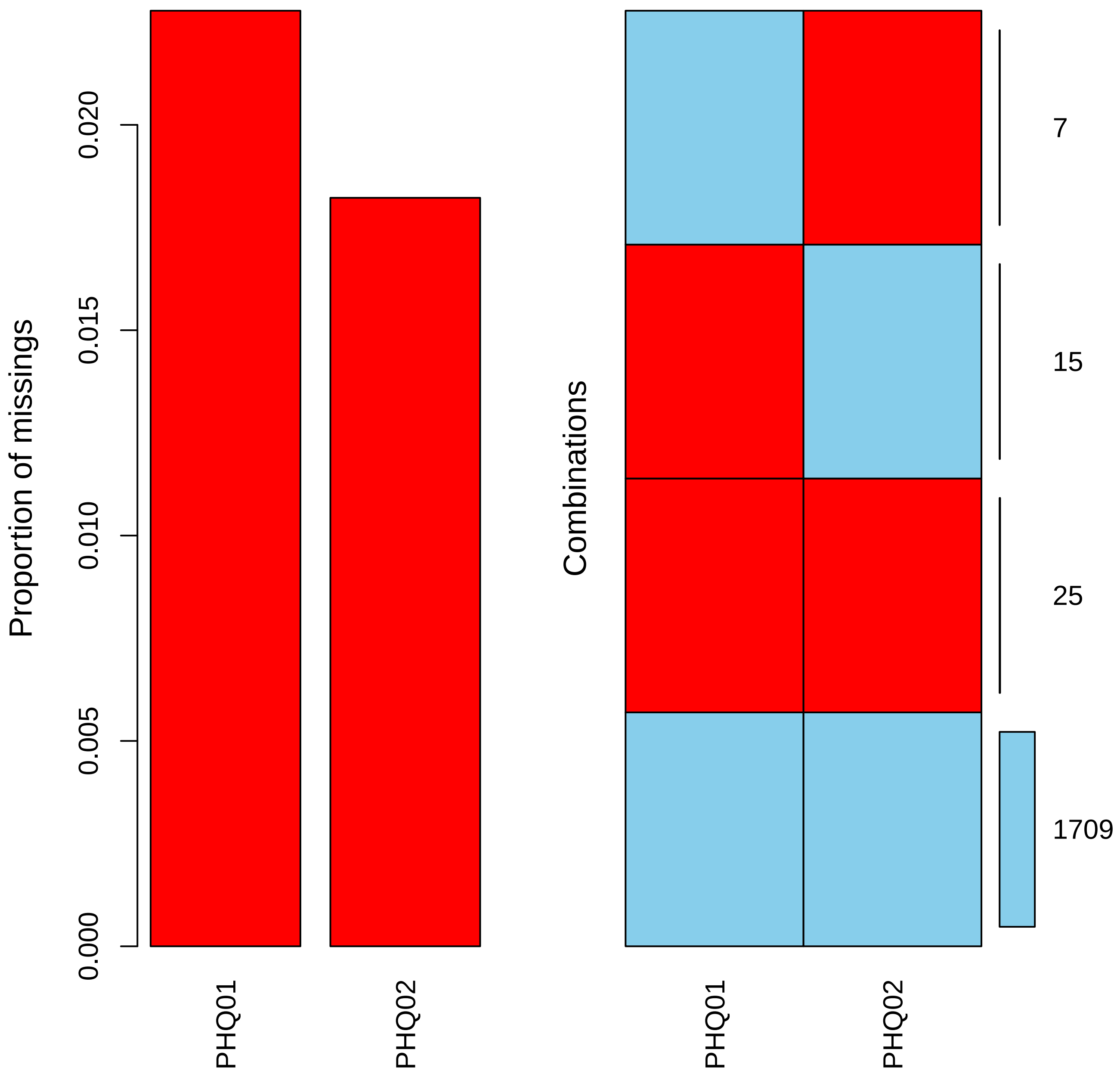

Proportion of missings
0.000
0.005
0.010
0.015
0.020
PHQ01
PHQ02
Combinations
7
15
25
1709
PHQ01
PHQ02

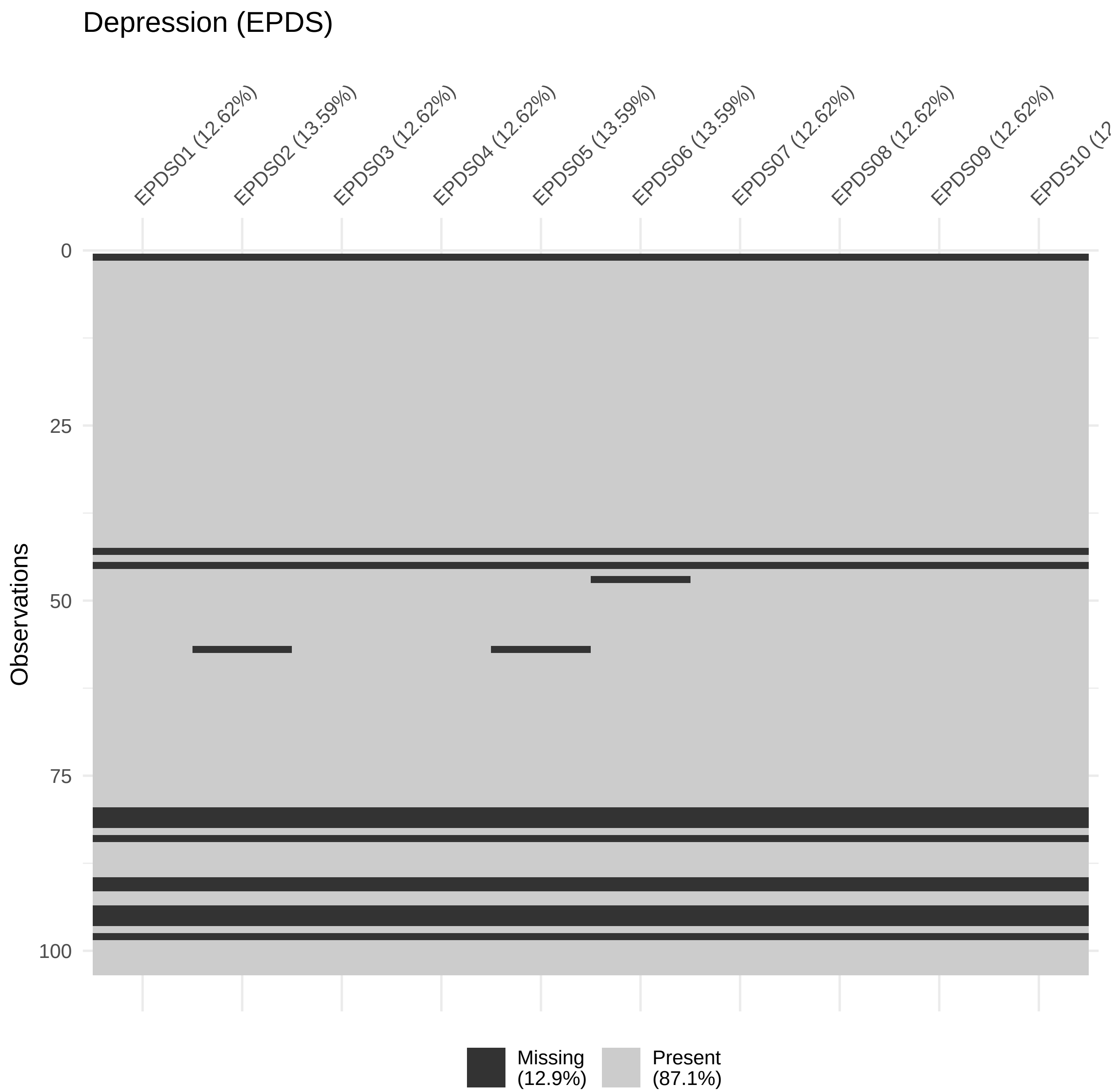
Depression (EPDS)
EPDS01 (12.62%)
EPDS02 (13.59%)
EPDS03 (12.62%)
EPDS04 (12.62%)
EPDS05 (13.59%)
EPDS06 (13.59%)
EPDS07 (12.62%)
EPDS08 (12.62%)
EPDS09 (12.62%)
EPDS10 (12
Observations
0
25
50
75
100
Missing
(12.9%)
Present
(87.1%)

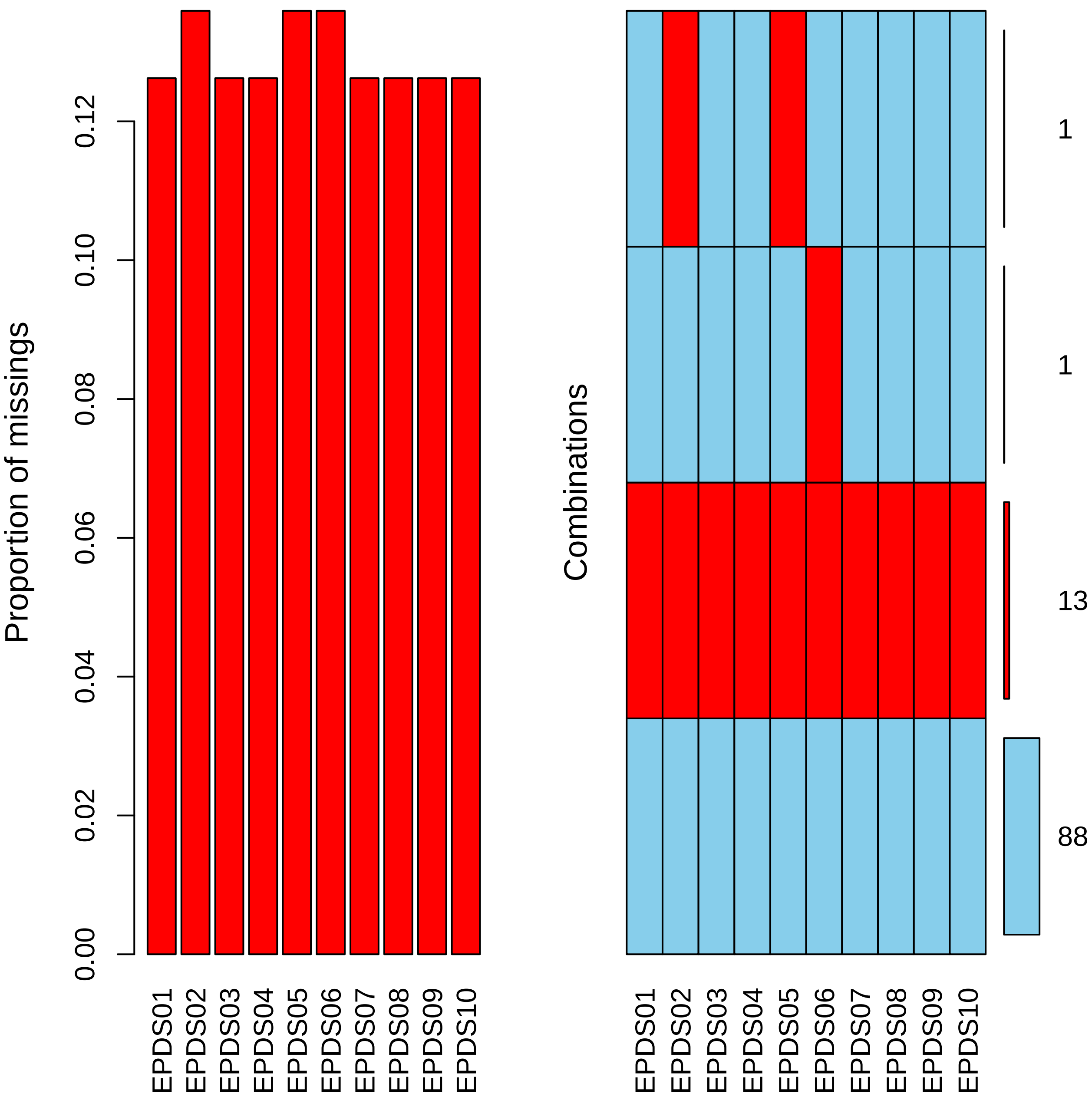

Proportion of missings
0.00
0.02
0.04
0.06
0.08
0.10
0.12
EPDS01
EPDS02
EPDS03
EPDS04
EPDS05
EPDS06
EPDS07
EPDS08
EPDS09
EPDS10
Combinations
1
1
13
88

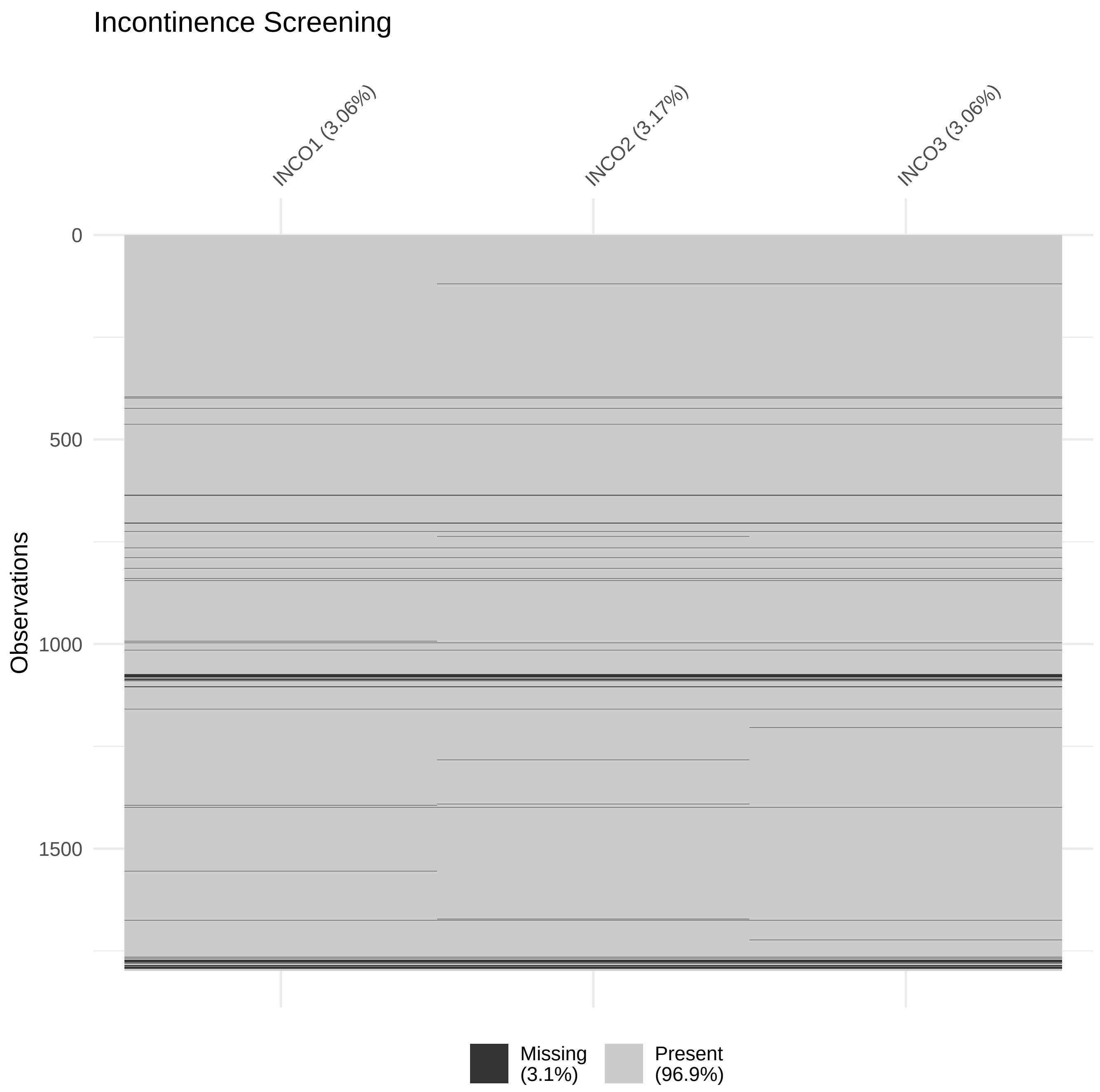
Incontinence Screening
INCO1 (3.06%)
INCO2 (3.17%)
INCO3 (3.06%)
0
500
1000
1500
Observations
Missing
(3.1%)
Present
(96.9%)

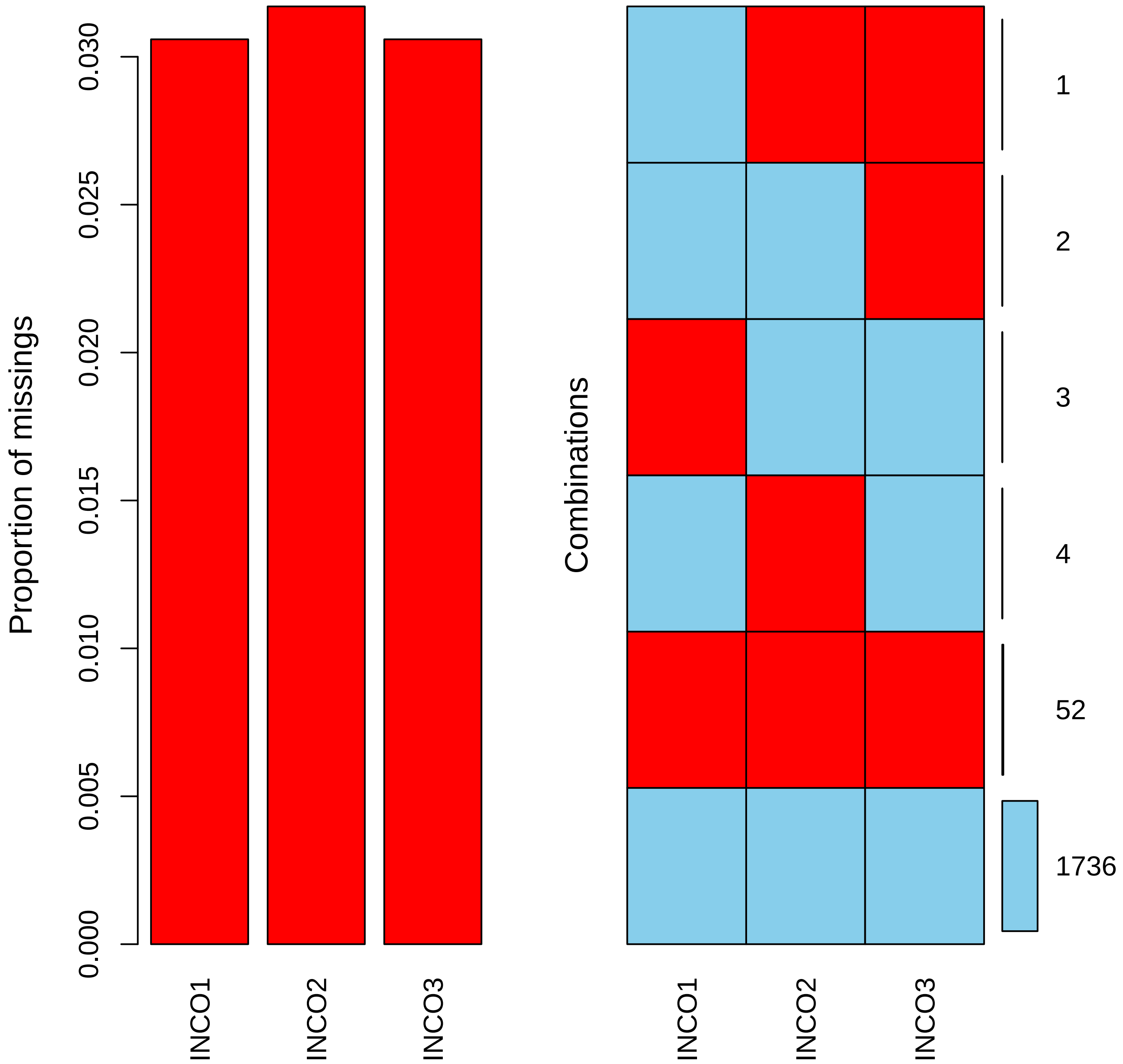
Proportion of missings
0.000
0.005
0.010
0.015
0.020
0.025
0.030
INCO1
INCO2
INCO3
Combinations
1
2
3
4
52
1736
INCO1
INCO2
INCO3

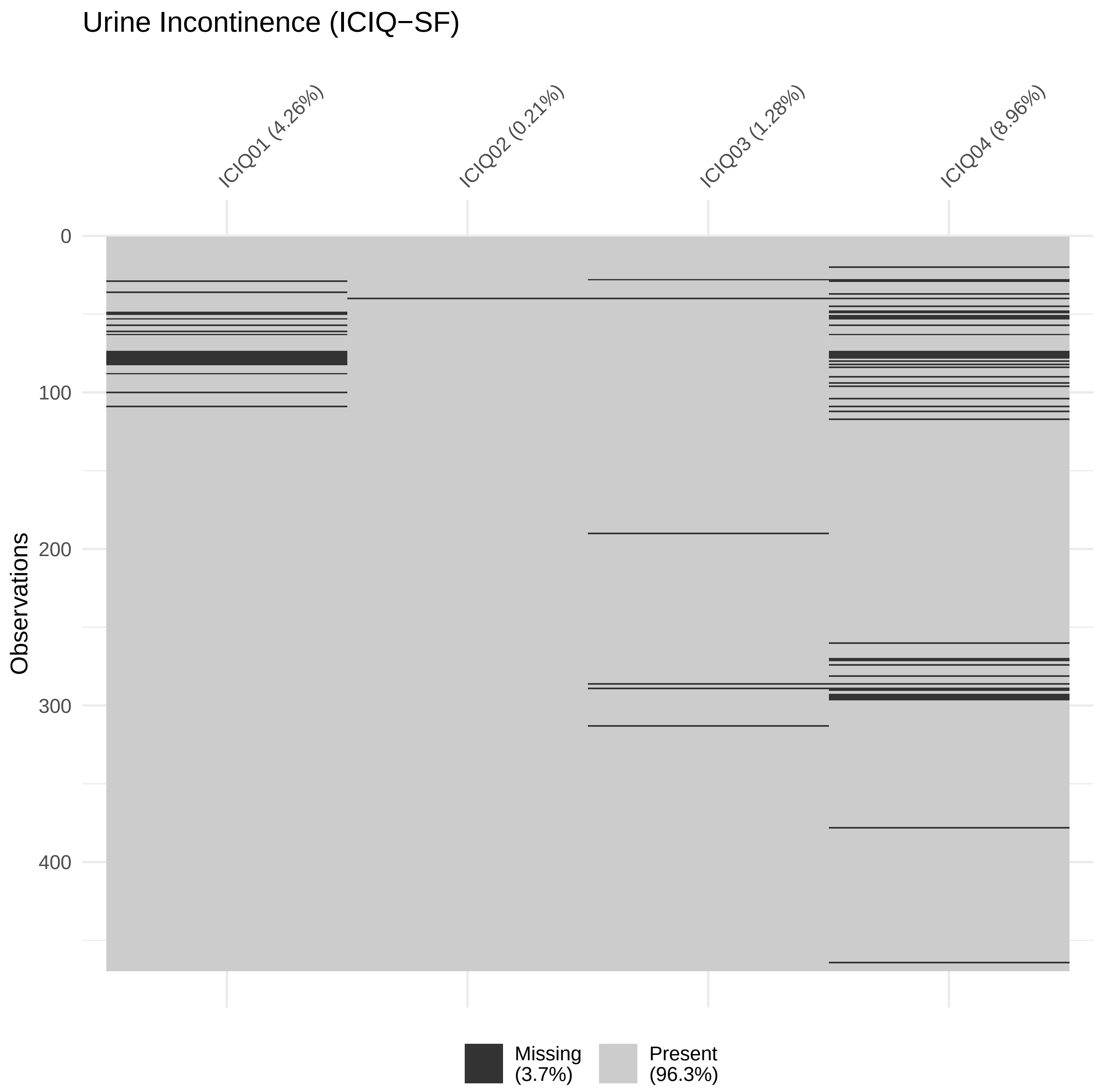

Urine Incontinence (ICIQ−SF)
ICIQ01 (4.26%)
ICIQ02 (0.21%)
ICIQ03 (1.28%)
ICIQ04 (8.96%)
Observations
0
100
200
300
400
Missing (3.7%)
Present (96.3%)

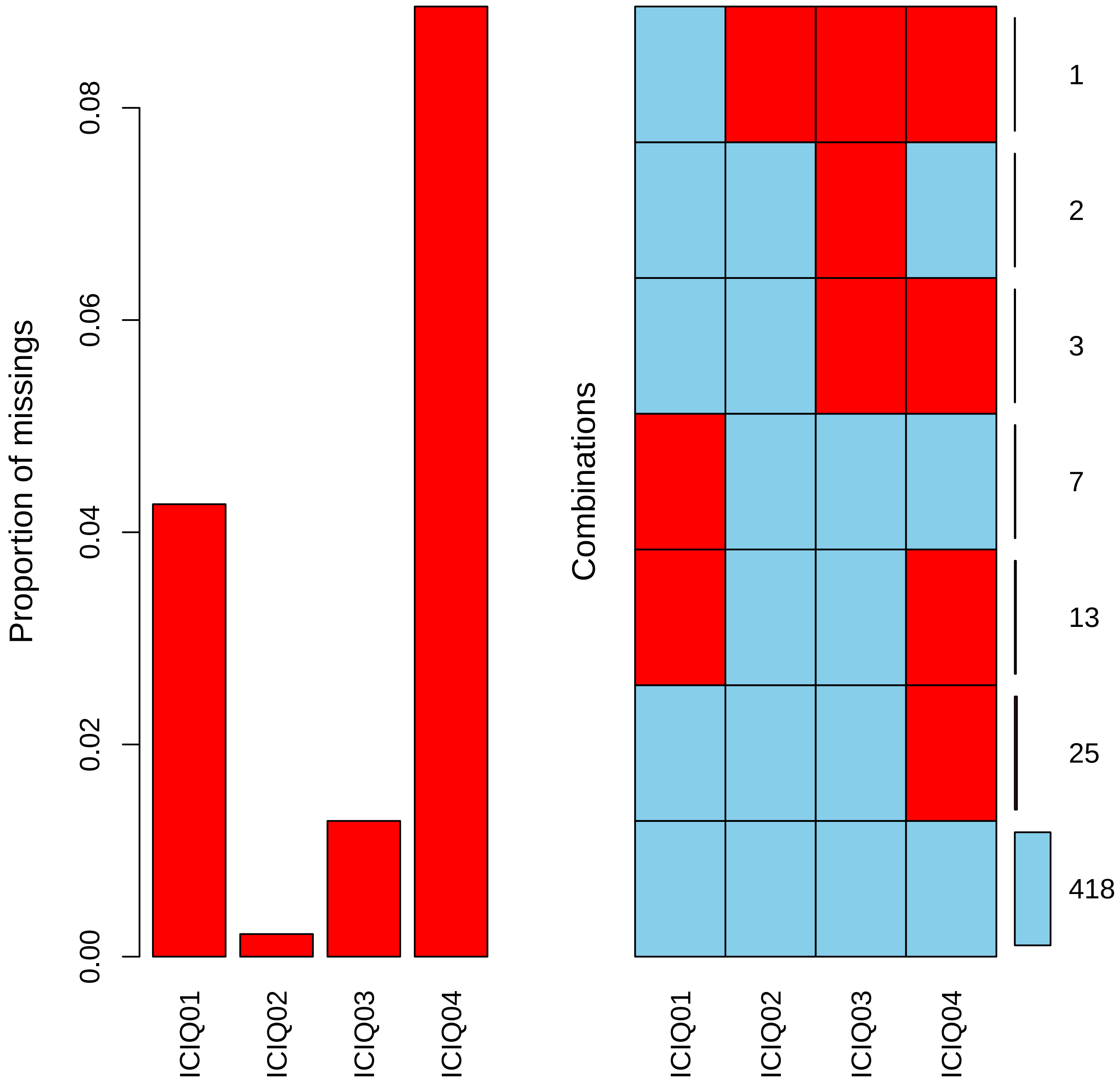

Proportion of missings
0.00
0.02
0.04
0.06
0.08
ICIQ01
ICIQ02
ICIQ03
ICIQ04
Combinations
ICIQ01
ICIQ02
ICIQ03
ICIQ04
1
2
3
7
13
25
418

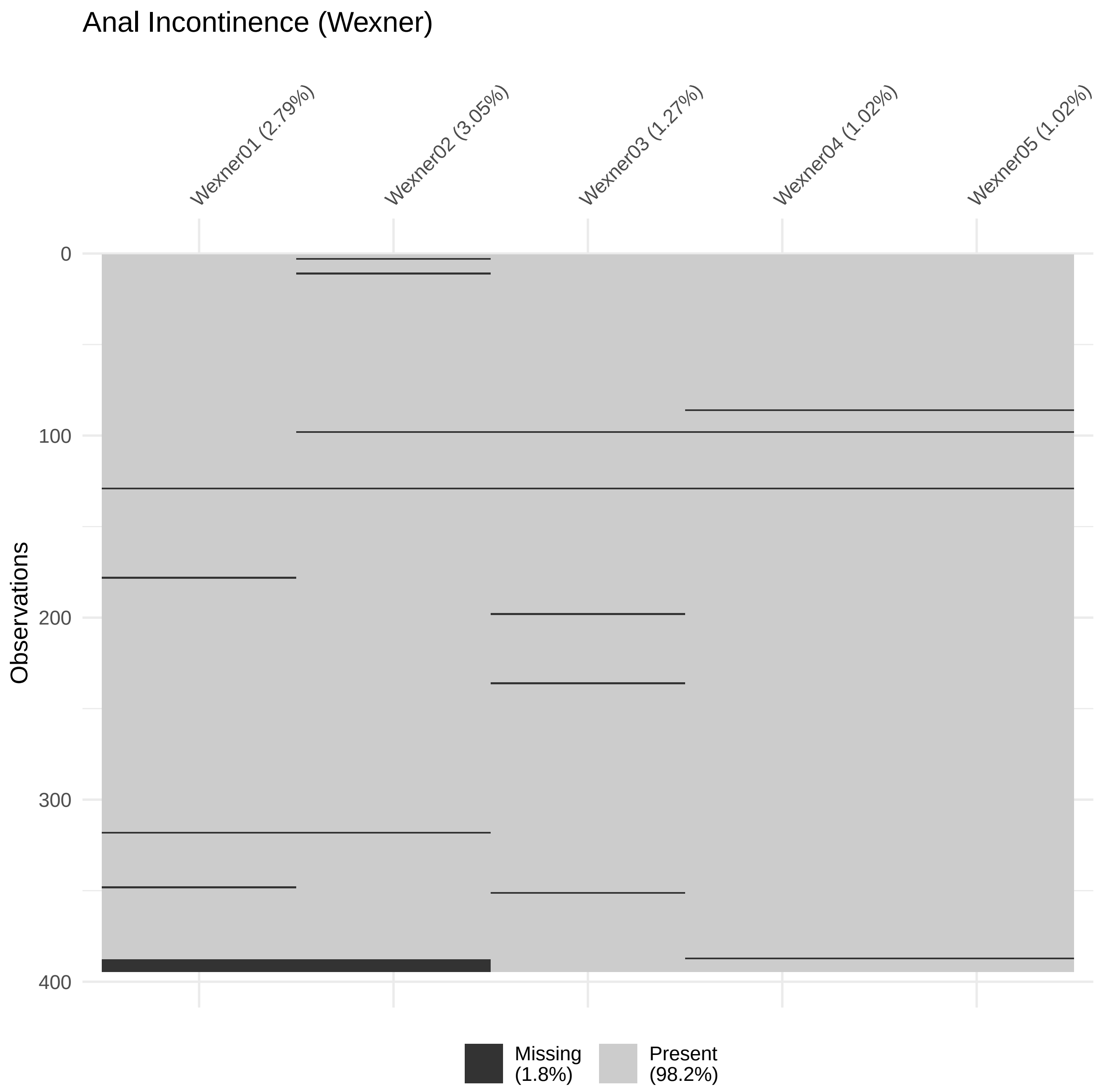
Anal Incontinence (Wexner)
Wexner01 (2.79%)
Wexner02 (3.05%)
Wexner03 (1.27%)
Wexner04 (1.02%)
Wexner05 (1.02%)
Observations
0
100
200
300
400
Missing (1.8%)
Present (98.2%)

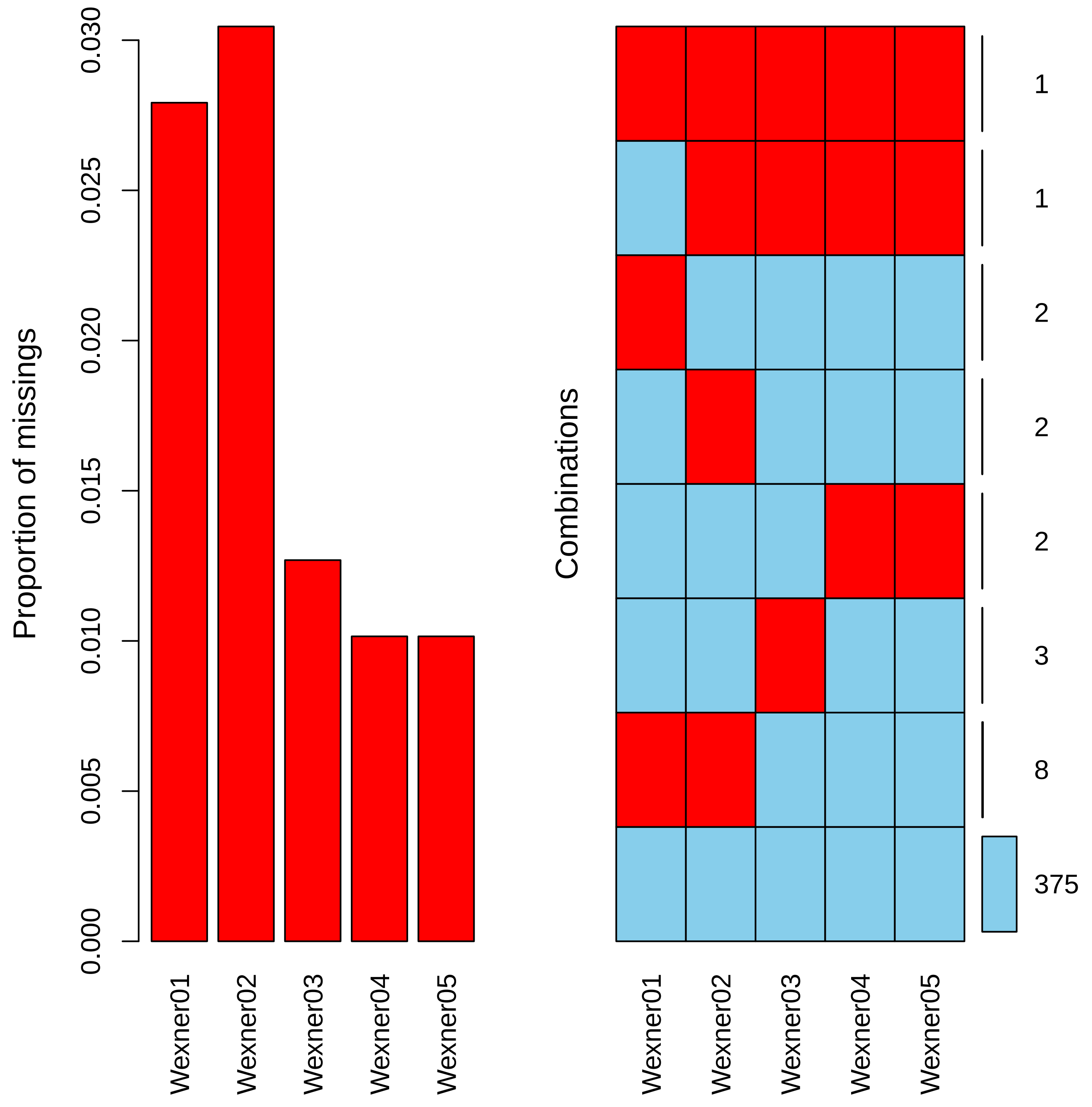
Proportion of missings
0.000
0.005
0.010
0.015
0.020
0.025
0.030
Wexner01
Wexner02
Wexner03
Wexner04
Wexner05
Combinations
1
1
2
2
2
3
8
375
Wexner01
Wexner02
Wexner03
Wexner04
Wexner05

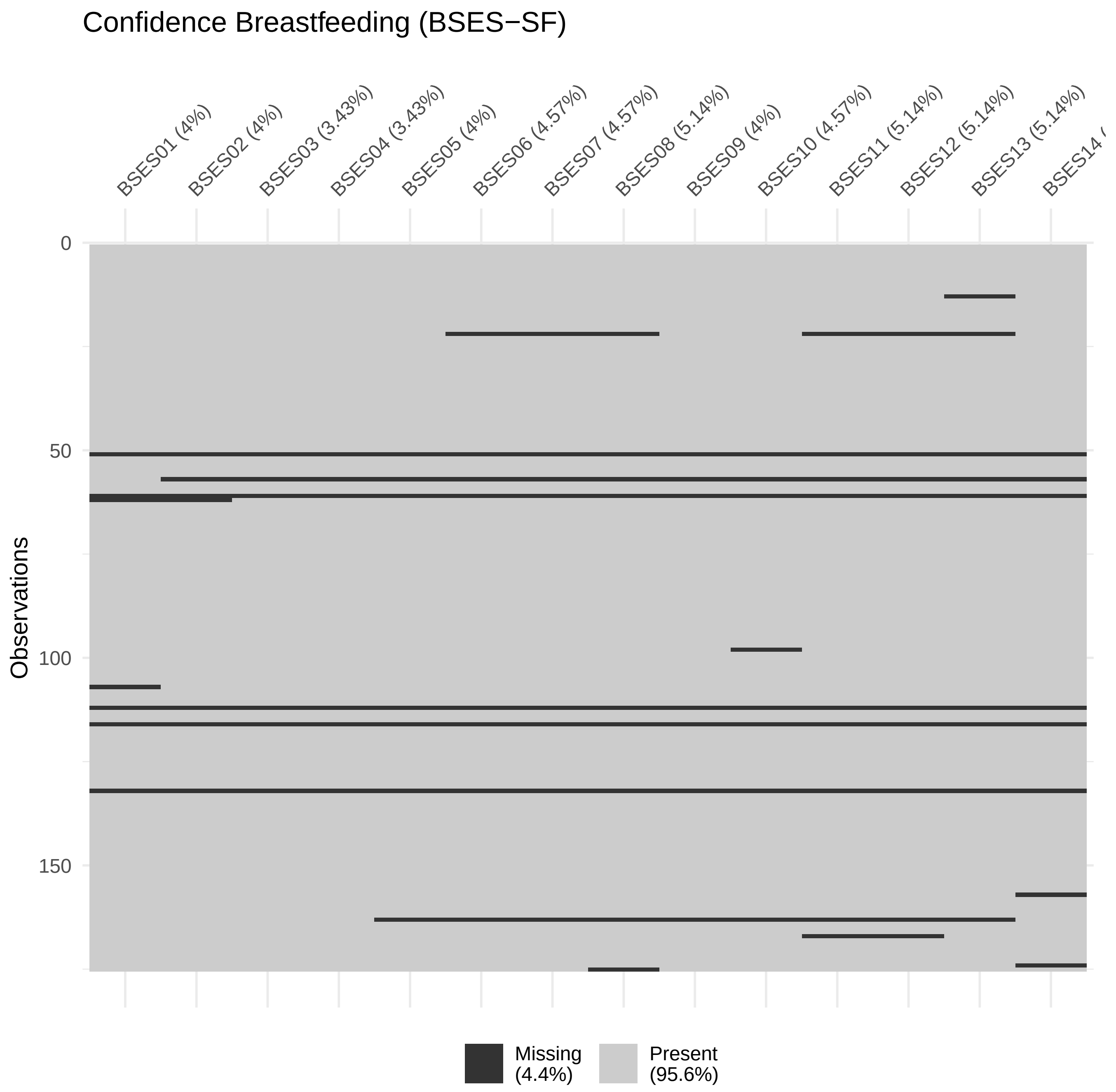

Confidence Breastfeeding (BSES−SF)
BSES01 (4%)
BSES02 (4%)
BSES03 (3.43%)
BSES04 (3.43%)
BSES05 (4%)
BSES06 (4.57%)
BSES07 (4.57%)
BSES08 (5.14%)
BSES09 (4%)
BSES10 (4.57%)
BSES11 (5.14%)
BSES12 (5.14%)
BSES13 (5.14%)
BSES14
Observations
0
50
100
150
Missing (4.4%)
Present (95.6%)

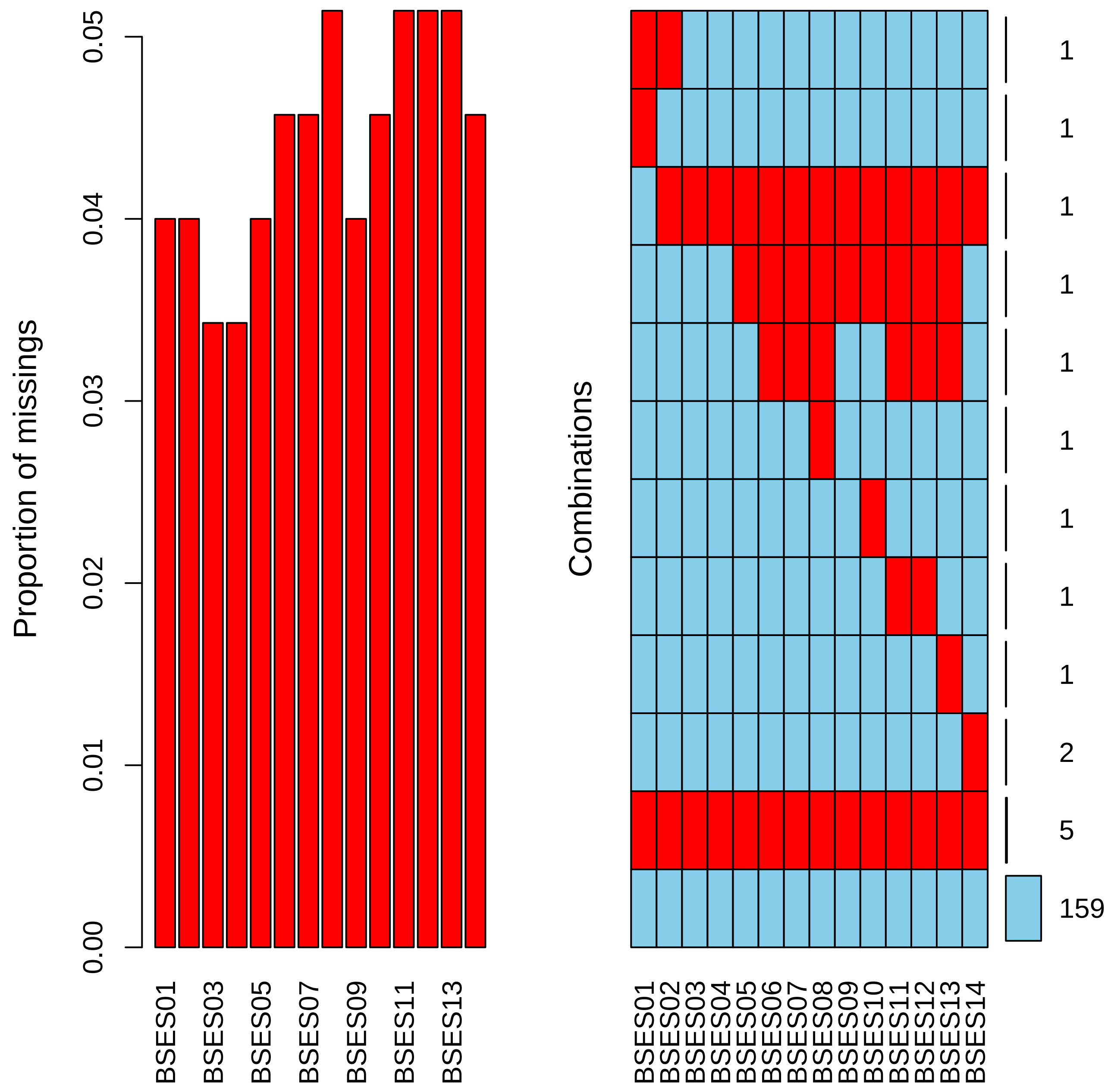

Proportion of missings
0.00
0.01
0.02
0.03
0.04
0.05
BSES01
BSES03
BSES05
BSES07
BSES09
BSES11
BSES13
Combinations
BSES01
BSES02
BSES03
BSES04
BSES05
BSES06
BSES07
BSES08
BSES09
BSES10
BSES11
BSES12
BSES13
BSES14
1
1
1
1
1
1
1
1
1
2
5
159

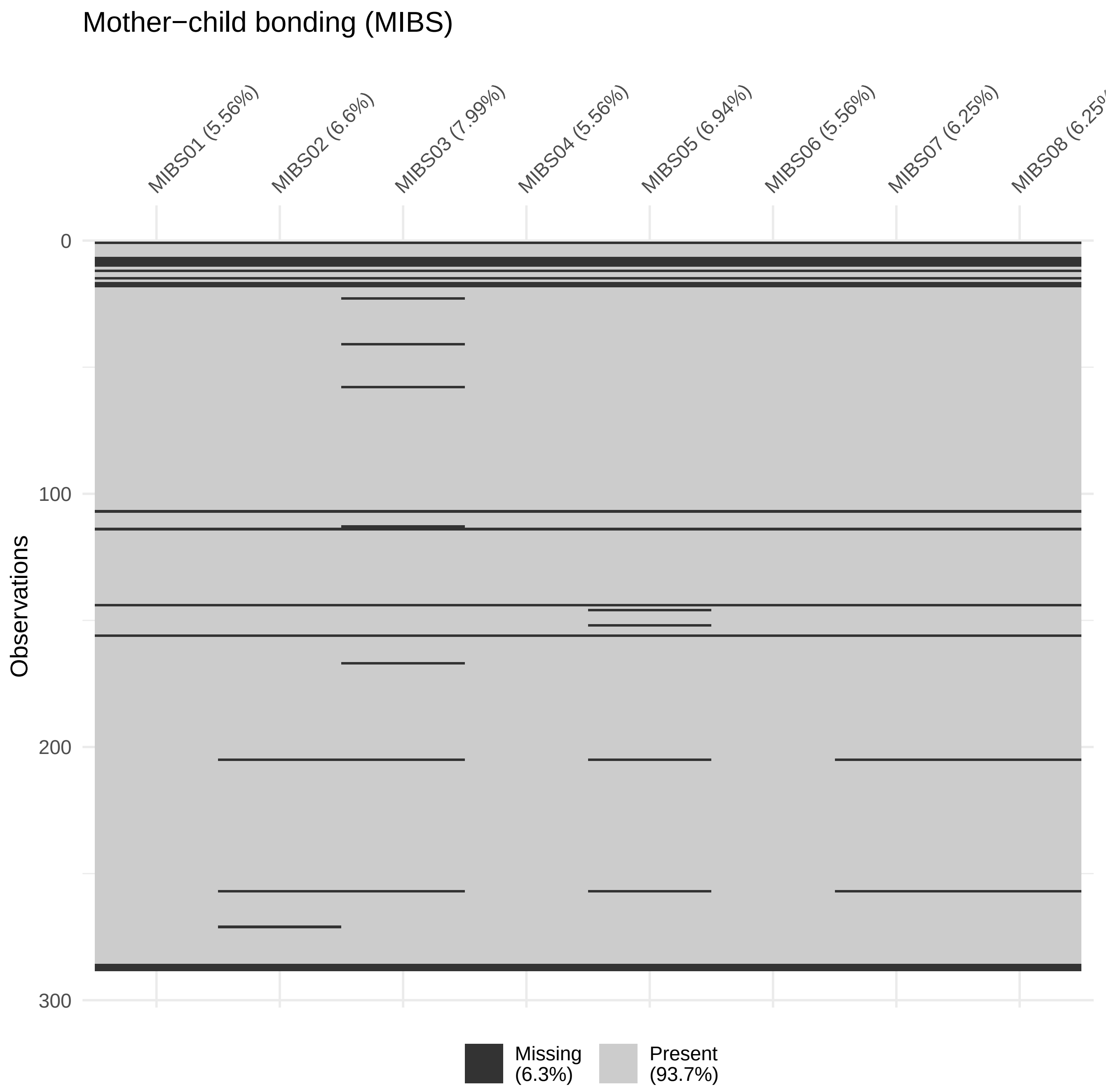

Mother−child bonding (MIBS)
MIBS01 (5.56%)
MIBS02 (6.6%)
MIBS03 (7.99%)
MIBS04 (5.56%)
MIBS05 (6.94%)
MIBS06 (5.56%)
MIBS07 (6.25%)
0
100
200
300
Observations
Missing
(6.3%)
Present
(93.7%)

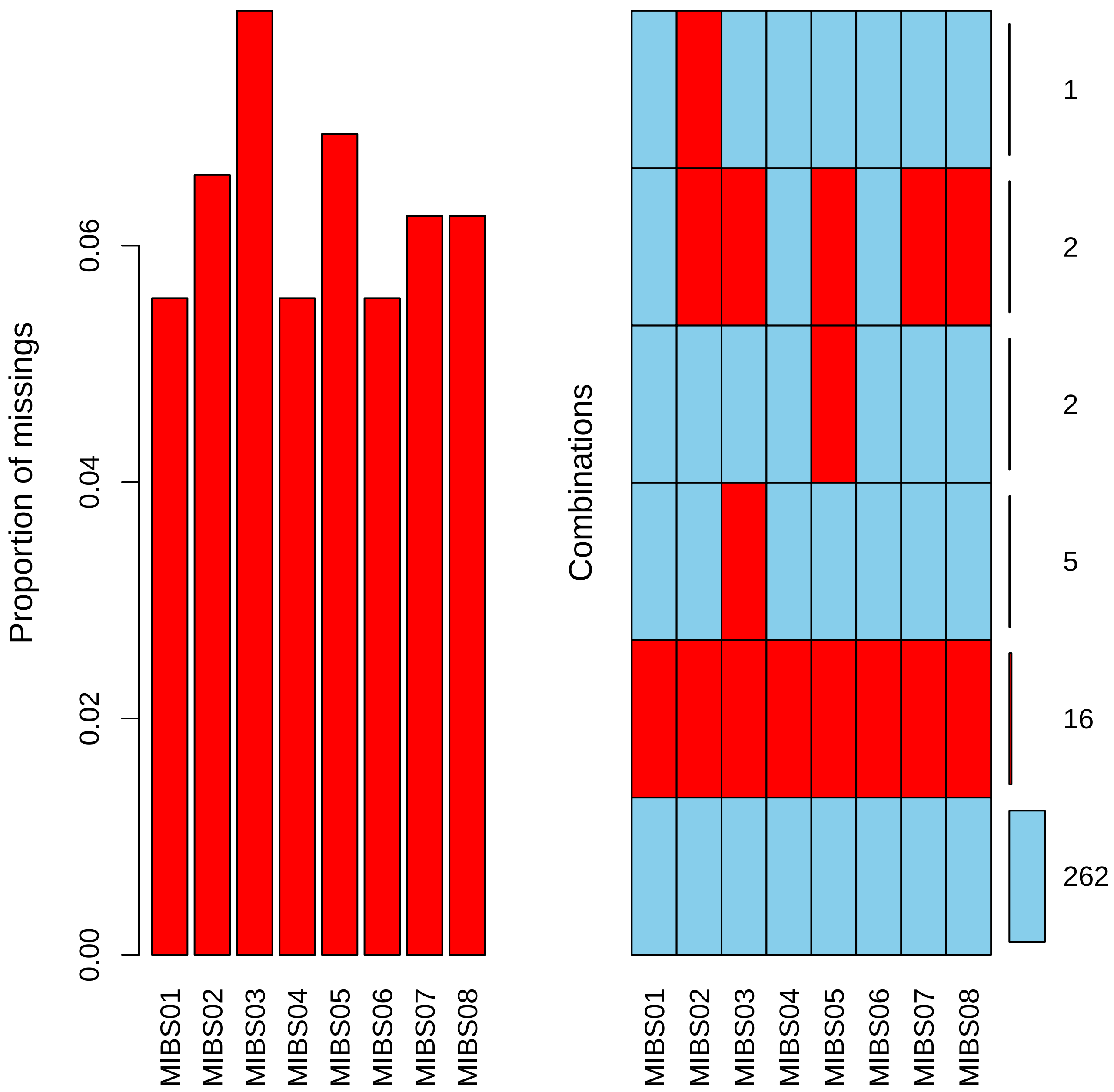
Proportion of missings
0.00
0.02
0.04
0.06
MIBS01
MIBS02
MIBS03
MIBS04
MIBS05
MIBS06
MIBS07
MIBS08
Combinations
1
2
2
5
16
262
MIBS01
MIBS02
MIBS03
MIBS04
MIBS05
MIBS06
MIBS07
MIBS08

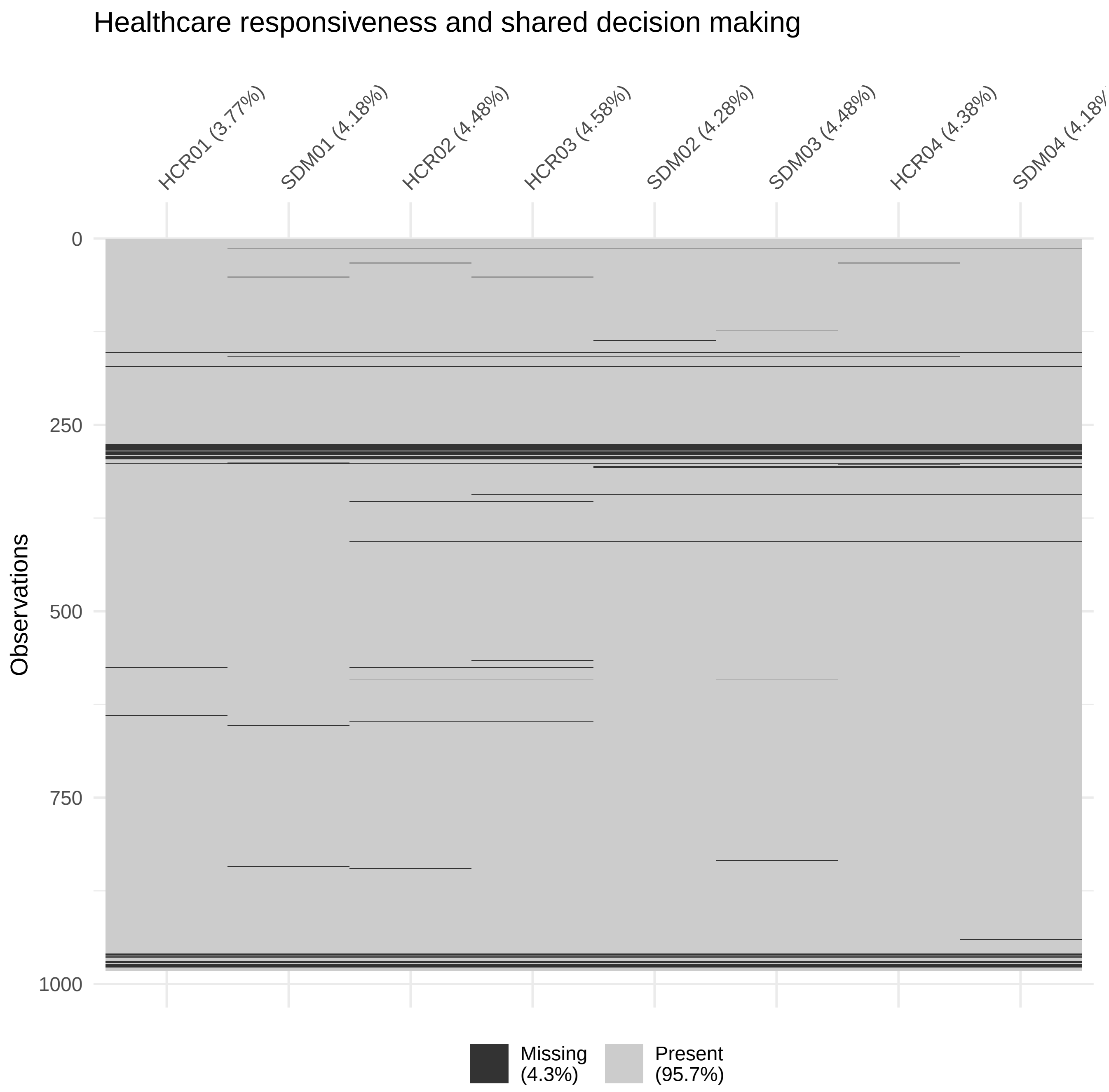
Healthcare responsiveness and shared decision making
HCR01 (3.77%)
SDM01 (4.18%)
HCR02 (4.48%)
HCR03 (4.58%)
SDM02 (4.28%)
SDM03 (4.48%)
HCR04 (4.38%)
SDM04 (4.18%
0
250
500
750
1000
Observations
Missing
(4.3%)
Present
(95.7%)

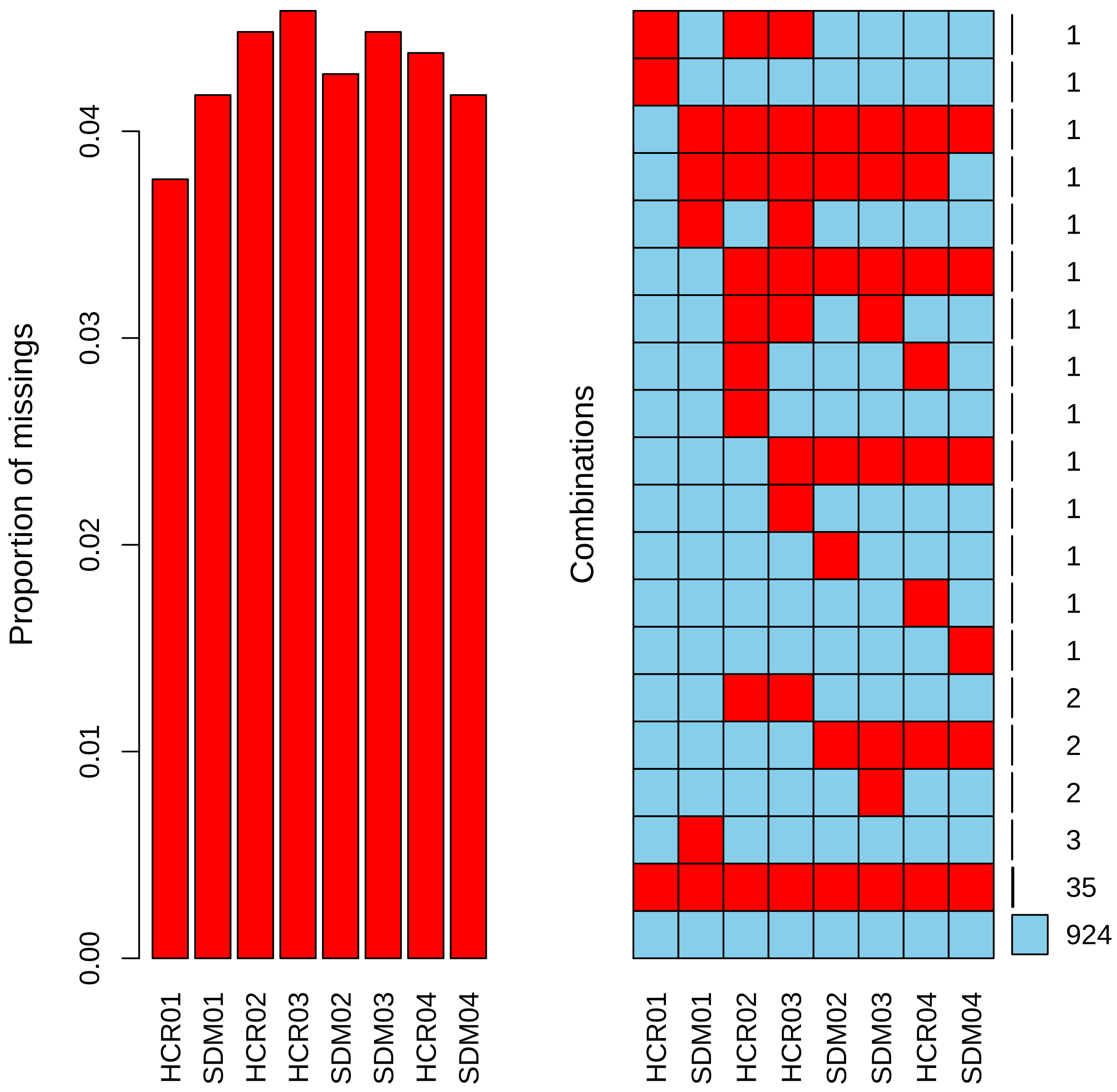

Proportion of missings
0.00
0.01
0.02
0.03
0.04
HCR01
SDM01
HCR02
HCR03
SDM02
SDM03
HCR04
SDM04
Combinations
1
1
1
1
1
1
1
1
1
1
1
1
1
1
2
2
2
3
35
924
HCR01
SDM01
HCR02
HCR03
SDM02
SDM03
HCR04
SDM04

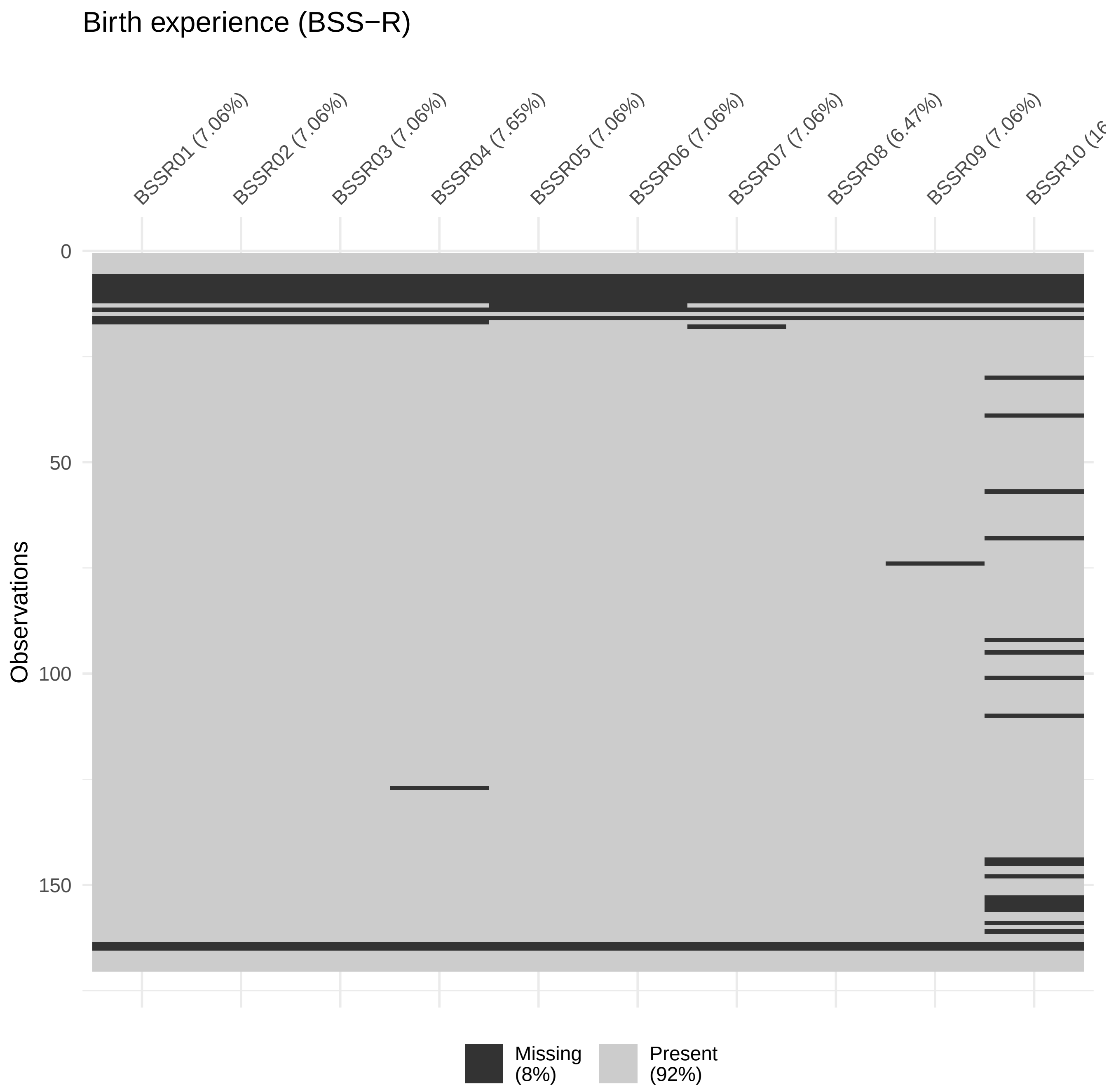
Birth experience (BSS−R)
BSSR01 (7.06%)
BSSR02 (7.06%)
BSSR03 (7.06%)
BSSR04 (7.65%)
BSSR05 (7.06%)
BSSR06 (7.06%)
BSSR07 (7.06%)
BSSR08 (6.47%)
BSSR09 (7.06%)
BSSR10 (16
Observations
0
50
100
150
Missing
(8%)
Present
(92%)

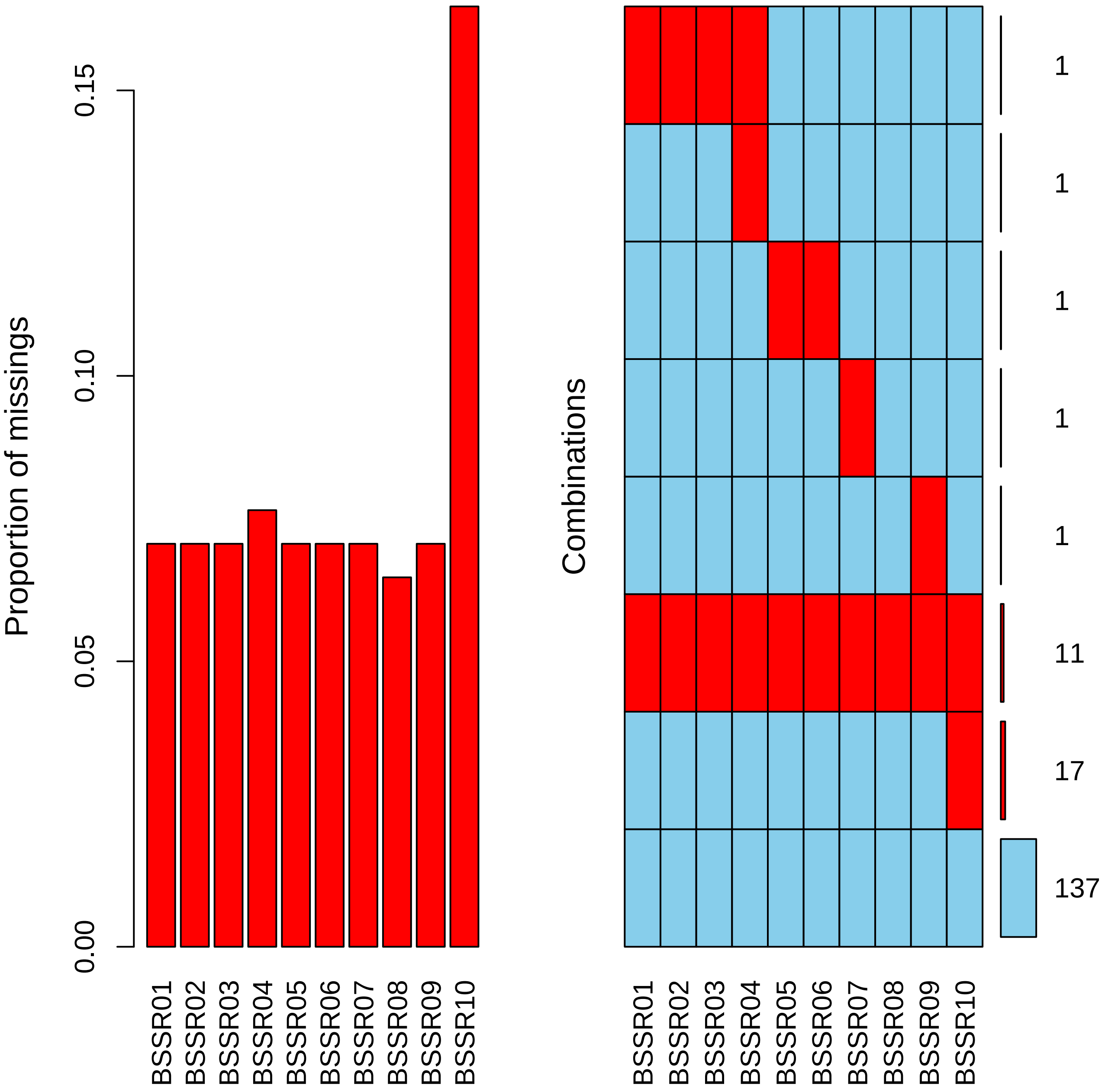

Proportion of missings
0.00
0.05
0.10
0.15
BSSR01
BSSR02
BSSR03
BSSR04
BSSR05
BSSR06
BSSR07
BSSR08
BSSR09
BSSR10
Combinations
BSSR01
BSSR02
BSSR03
BSSR04
BSSR05
BSSR06
BSSR07
BSSR08
BSSR09
BSSR10
1
1
1
1
1
11
17
137

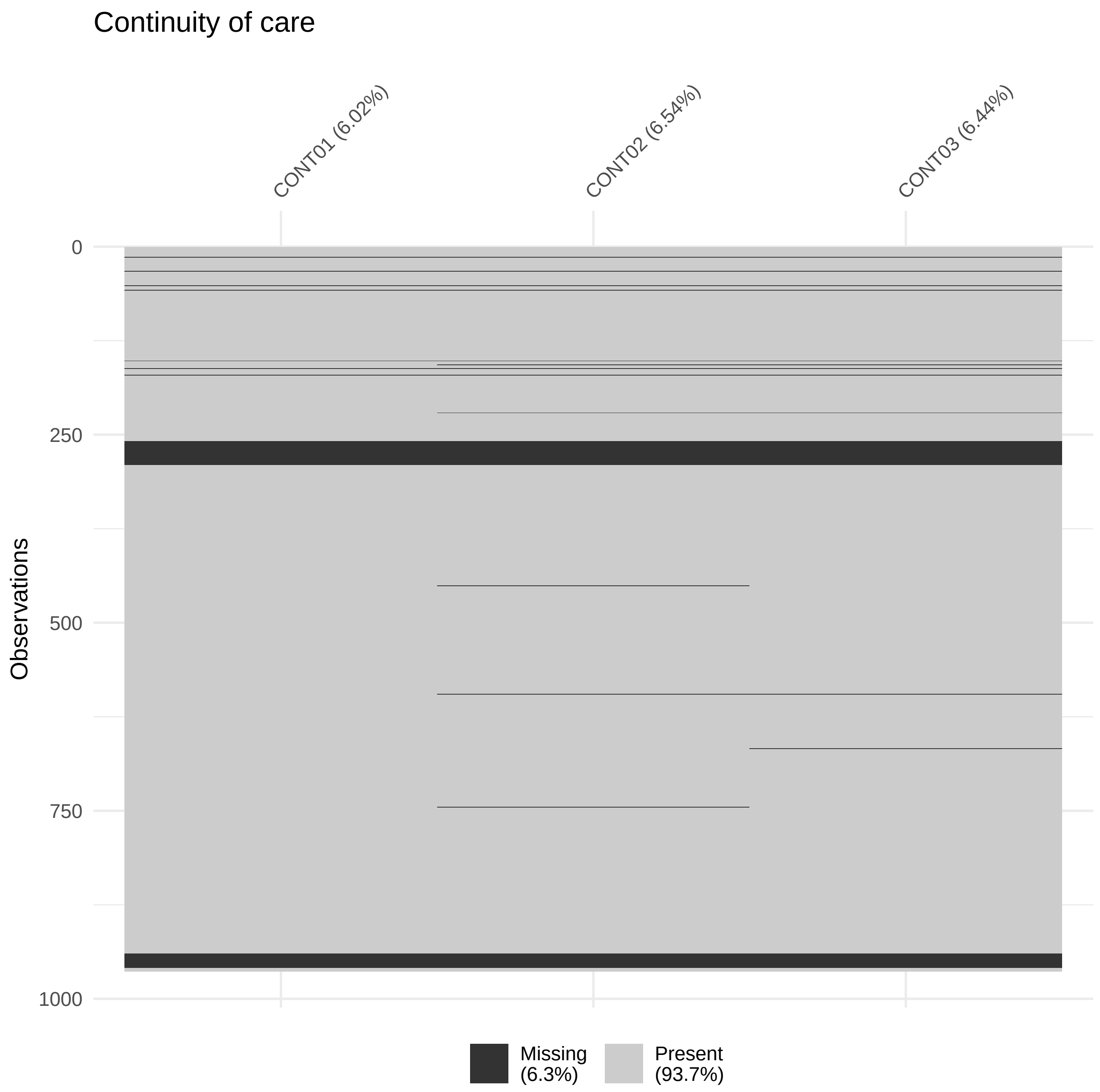

Continuity of care
CONT01 (6.02%)
CONT02 (6.54%)
CONT03 (6.44%)
0
250
500
750
1000
Observations
Missing
(6.3%)
Present
(93.7%)

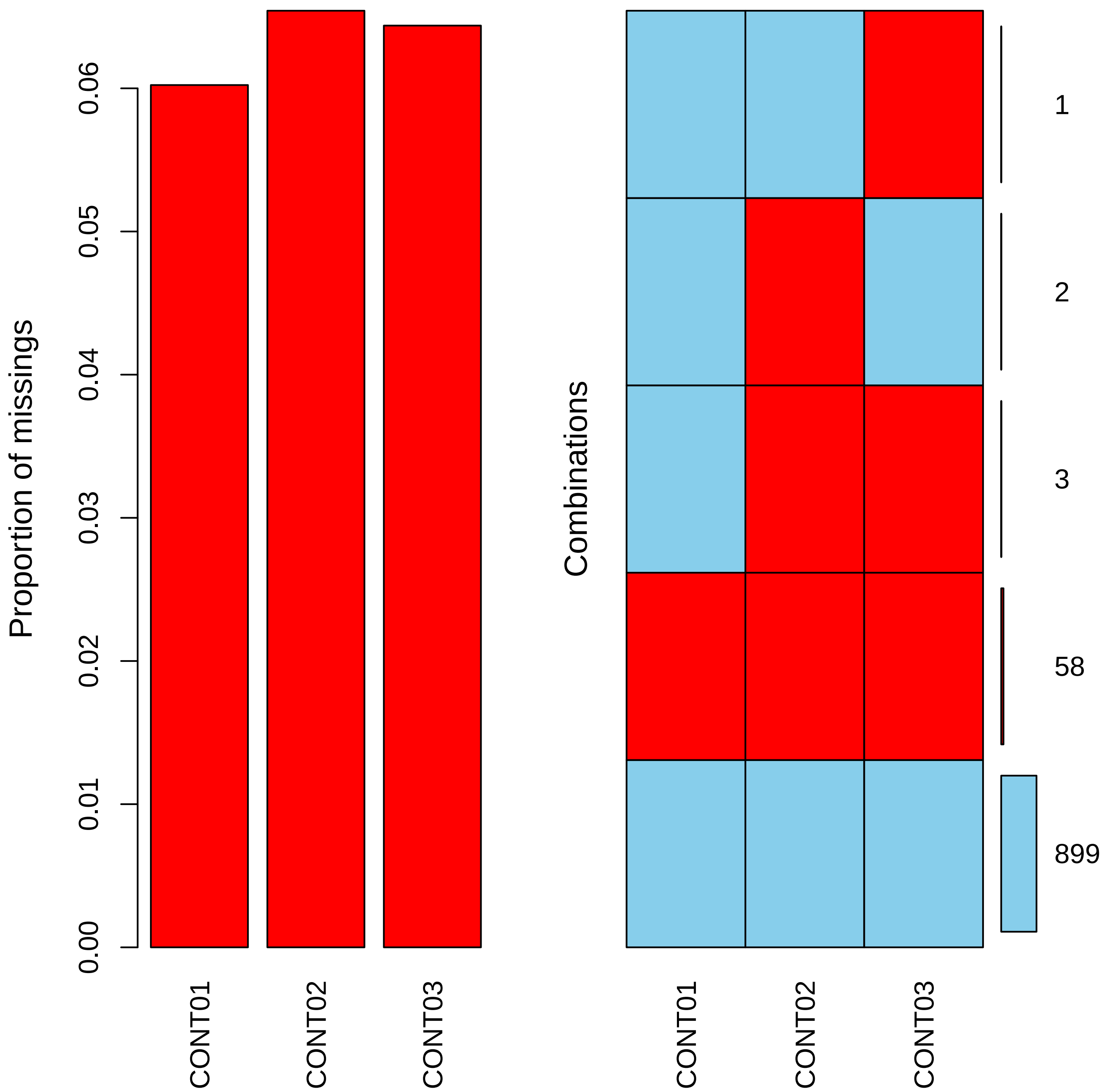

Proportion of missings
0.00
0.01
0.02
0.03
0.04
0.05
0.06
CONT01
CONT02
CONT03
Combinations
CONT01
CONT02
CONT03
1
2
3
58
899
